# Exploiting the GTEx resources to decipher the mechanisms at GWAS loci

**DOI:** 10.1186/s13059-020-02252-4

**Published:** 2021-01-26

**Authors:** Alvaro N. Barbeira, Rodrigo Bonazzola, Eric R. Gamazon, Yanyu Liang, YoSon Park, Sarah Kim-Hellmuth, Gao Wang, Zhuoxun Jiang, Dan Zhou, Farhad Hormozdiari, Boxiang Liu, Abhiram Rao, Andrew R. Hamel, Milton D. Pividori, François Aguet, GTEx GWAS Working Group, Lisa Bastarache, Daniel M. Jordan, Marie Verbanck, Ron Do, GTEx Consortium, Matthew Stephens, Kristin Ardlie, Mark McCarthy, Stephen B. Montgomery, Ayellet V. Segrè, Christopher D. Brown, Tuuli Lappalainen, Xiaoquan Wen, Hae Kyung Im

**Affiliations:** 1 Section of Genetic Medicine, Department of Medicine, The University of Chicago, Chicago, IL USA; 2 Division of Genetic Medicine, Department of Medicine, Vanderbilt University Medical Center, Nashville, TN USA; 3 Data Science Institute, Vanderbilt University, Nashville, TN USA; 4 Clare Hall, University of Cambridge, Cambridge, UK; 5 MRC Epidemiology Unit, University of Cambridge, Cambridge, UK; 6 Department of Genetics, University of Pennsylvania, Perelman School of Medicine, Philadelphia, PA USA; 7 Department of Systems Pharmacology and Translational Therapeutics, University of Pennsylvania, Perelman School of Medicine, Philadelphia, PA USA; 8 Statistical Genetics, Max Planck Institute of Psychiatry, Munich, Germany; 9 New York Genome Center, New York, NY USA; 10 Department of Systems Biology, Columbia University, New York, NY USA; 11 Department of Human Genetics, University of Chicago, Chicago, IL USA; 12 The Broad Institute of MIT and Harvard, Cambridge, MA USA; 13 Department of Epidemiology, Harvard T.H. Chan School of Public Health, Boston, MA USA; 14 Department of Biology, Stanford University, Stanford, 94305 CA USA; 15 Ocular Genomics Institute, Massachusetts Eye and Ear, Harvard Medical School, Boston, MA USA; 16 Department of Biomedical Informatics, Department of Medicine, Vanderbilt University, Nashville, TN USA; 17 Center for Human Genetics Research, Department of Molecular Physiology and Biophysics, Vanderbilt University School of Medicine, Nashville, TN USA; 18 Department of Genetics and Genomic Sciences, Icahn School of Medicine at Mount Sinai, New York, NY USA; 19 Institute for Genomics and Multiscale Biology, Icahn School of Medicine at Mount Sinai, New York, NY USA; 20 The Charles Bronfman Institute for Personalized Medicine, Icahn School of Medicine at Mount Sinai, New York, NY USA; 21 Université de Paris - EA 7537 BIOSTM, Paris, France; 22 University of Oxford, Oxford, UK; 23 Department of Genetics, Stanford University, Stanford, CA USA; 24 Department of Pathology, Stanford University, Stanford, CA USA; 25 Department of Biostatistics, University of Michigan, Ann Arbor, MI USA

## Abstract

The resources generated by the GTEx consortium offer unprecedented opportunities to advance our understanding of the biology of human diseases. Here, we present an in-depth examination of the phenotypic consequences of transcriptome regulation and a blueprint for the functional interpretation of genome-wide association study-discovered loci. Across a broad set of complex traits and diseases, we demonstrate widespread dose-dependent effects of RNA expression and splicing. We develop a data-driven framework to benchmark methods that prioritize causal genes and find no single approach outperforms the combination of multiple approaches. Using colocalization and association approaches that take into account the observed allelic heterogeneity of gene expression, we propose potential target genes for 47% (2519 out of 5385) of the GWAS loci examined.

**Supplementary Information:**

The online version contains supplementary material available at (10.1186/s13059-020-02252-4).

## Introduction

In the last decade, the number of reproducible genetic associations with complex human traits that have emerged from genome-wide association studies (GWAS) has substantially grown. Many of the identified associations lie in non-coding regions of the genome, suggesting that they influence disease pathophysiology and complex traits via gene regulatory changes. Integrative studies of molecular quantitative trait loci (QTL) [1] have established gene expression as a key intermediate molecular phenotype, and improved functional interpretation of GWAS findings, spanning immunological diseases [2], various cancers [3, 4], lipid traits [5, 6], and a broad array of other complex traits.

Large-scale international efforts such as the Genotype-Tissue Expression (GTEx) Consortium have provided an atlas of the regulatory landscape of gene expression and splicing variation in a broad collection of primary human tissues [7–9]. Nearly all protein-coding genes in the genome now have at least one local variant associated with expression changes and the majority also have common variants affecting alternative splicing (FDR < 5%) [9]. In parallel, there has been an explosive growth in the number of genetic discoveries across a large number of traits, prompting the development of integrative approaches to characterize the function of GWAS findings [10–14]. Nevertheless, our understanding of underlying biological mechanisms for most complex traits substantially lags behind the improved efficiency of the discovery of genetic associations, made possible by large-scale biobanks and GWAS meta-analyses.

One of the primary tools for the functional interpretation of GWAS associations has been the integrative analysis of molecular QTLs. Colocalization approaches that seek to establish shared causal variants (e.g., eCaviar [15], *enloc* [16], and *coloc* [17]), enrichment analysis (S-LDSC [18] and QTLEnrich [11]), or mediation and association methods (SMR [12], TWAS [13], and PrediXcan [19]) have provided important insights, but they are often used in isolation, and there have been limited prior assessments of power and error rates associated with each [20]. Their applications often fail to provide a comprehensive, biologically interpretable view across multiple methods, traits, and tissues or offer guidelines that are generalizable to other contexts. Thus, a comprehensive assessment of expression and splicing QTLs for their contributions to disease susceptibility and other complex traits requires the development of novel methodologies with improved resolution and interpretability.

Here, we present methods and resources that help elucidate how genetic variants associated with gene expression (cis-eQTLs) or splicing (cis-sQTLs) contribute to, or mediate, the functional mechanisms underlying a wide array of complex diseases and quantitative traits. Since splicing QTLs have largely been understudied, we perform a comprehensive integrative study of this class of QTLs, in a broad collection of tissues, and disease associations. We provide predictions of functional mechanisms for 74 distinct complex traits from 87 GWA study results and demonstrate independent validation and evaluation of findings using likely causal gene-disease relationships in the Online Mendelian Inheritance of Man (OMIM) database. Notably, we find widespread dose-dependent effects of cis-QTLs on traits through multiple lines of evidence. We examine the importance of considering, or correcting for, false functional links attributed to GWAS loci due to neighboring but distinct causal variants. We call this confounding LD contamination for the remainder of the paper. To identify predicted causal effects among the complex trait-associated QTLs, we conduct systematic evaluation across different methods. Furthermore, we provide guidelines for employing complementary methods to map the regulatory mechanisms underlying genetic associations with complex traits.

## Mapping the regulatory landscape of complex traits

**Fig. 1:**
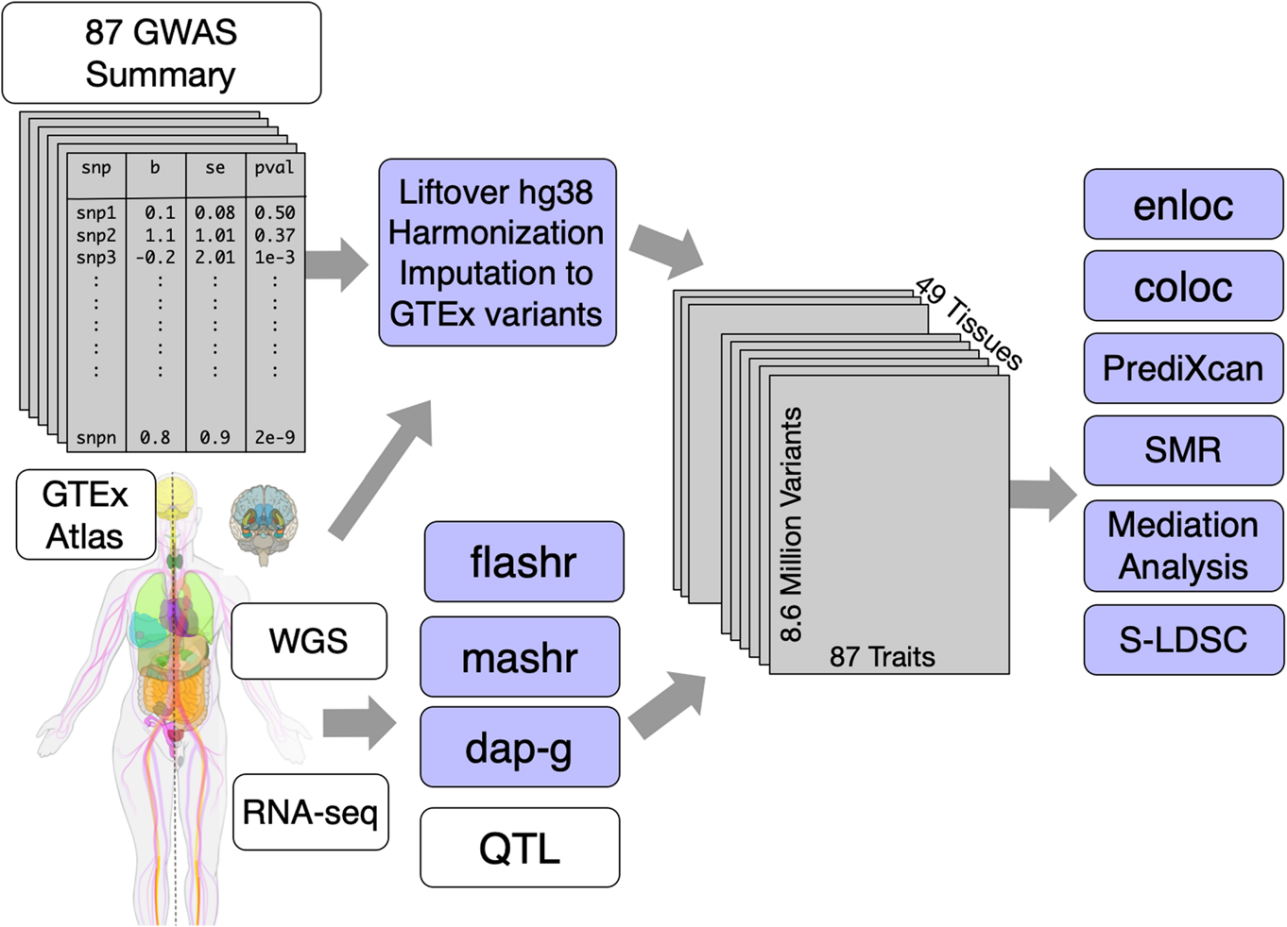
Overview of workflow for mapping complex trait-associated QTLs. Full variant association summary statistics results from 114 GWAS were downloaded, standardized, and imputed to the GTEx v8 WGS variant calls (maf > 0.01) for analyses. A total of 8.87 million imputed and genotyped variants were investigated to identify trait-associated QTLs. A total of 49 tissues, 87 studies (74 distinct traits), and 23,268 protein-coding genes and lncRNAs remained after stringent quality assurance protocols and selection criteria. A wide array of complex trait classes, including cardiometabolic, anthropometric, and psychiatric traits, were included

The final GTEx data release (v8) included 54 primary human tissues, 49 of which included at least 70 samples with both whole genome sequencing (WGS) and tissue-specific RNA-seq data. A total of 15,253 samples from 838 individuals were used for cis-QTL mapping (Fig. 1) [9]. In addition to the expression quantitative trait loci (eQTL) mapping, we also evaluated genetic variation associated with alternative splicing (sQTL) and their impact on complex traits.

We downloaded and processed 114 publicly available GWAS datasets with genome-wide variant association summary statistics (here onwards, summary statistics). After data harmonization, format standardization, missing data imputation, and other quality assurance steps (Additional file 1: Fig. S1, Fig. S2, and Fig. S3), we retained 87 datasets representing 74 distinct complex traits including cardiometabolic, hematologic, neuropsychiatric, and anthropometric traits (Additional file 1: Fig. S4). We provide the full list of datasets used in our study and all processing scripts as a resource to the community (Additional file 2: Table S1 and Additional file 1: Table S2).

Using these resources, we sought to identify likely causal associations among these gene- and alternatively spliced transcript-associated variants (eVariants and sVariants, respectively). For this purpose, we applied colocalization, enrichment, and association analyses, and provide a resource to enable investigations into gene prioritization approaches for disease associations.

**Fig. 2:**
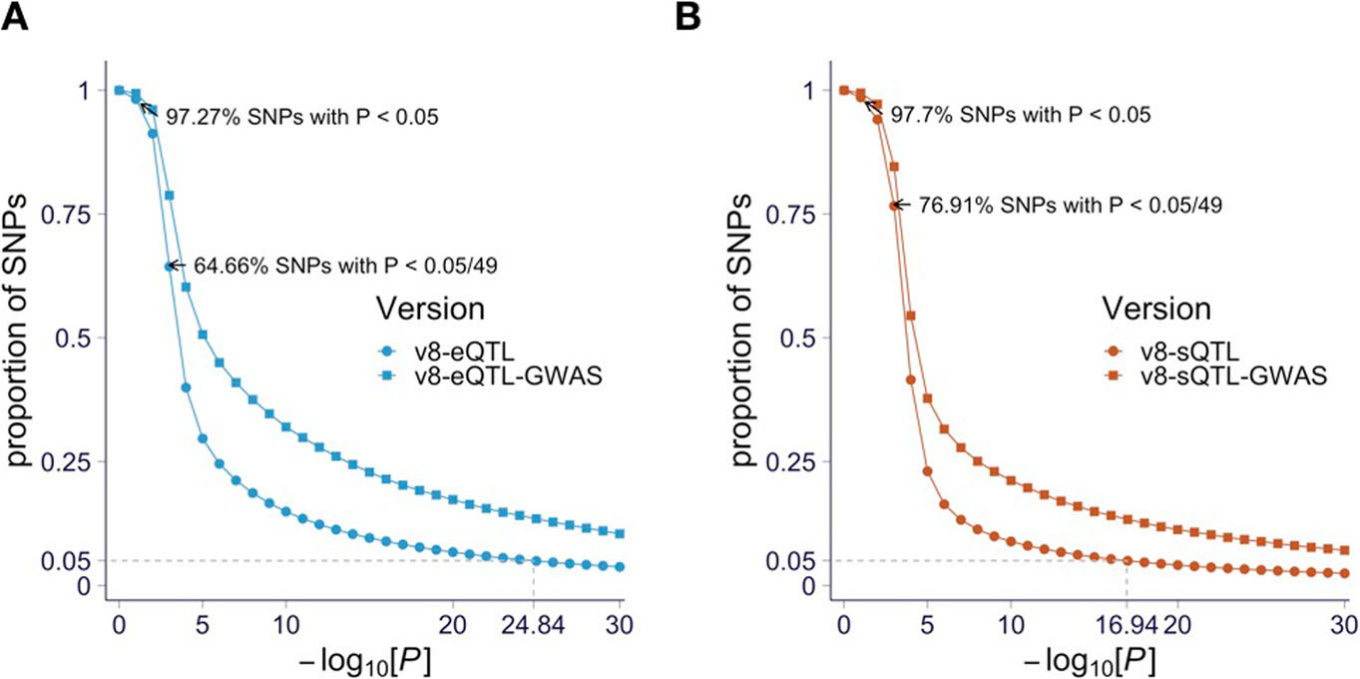
Expression and splicing QTL enrichment among GWAS variants. The proportion of genetic variants associated with gene expression (**a**) and splicing (**b**) of at least one gene in at least one tissue for each *p* value cutoff (on *x*-axis in − log10(*p*) scale) is shown. The proportions for all tested variants are shown as circles, and the proportions for the GWAS catalog variants are shown as squares

Gene expression and alternative splicing dysregulations have been proposed as the underlying mechanism of the association signals in many diseases [5, 11, 21–24]. Similar to previous reports [8], we observed robust and widespread enrichment of eQTLs and sQTLs among disease-associated variants (Fig. 2). This observation suggests a causal role for expression and splicing regulation in complex traits. Figure 2 also illustrates the dangers of using a naive approach to assigning causal genes to GWAS variants that are associated with expression or splicing, especially when using loose *p* value thresholds. For example, with a *p* value threshold of 0.05, over 97% of common variants will be assigned some gene in some tissue associated at that level.

## Dose-dependent regulatory effects of expression and alternative splicing on complex traits

**Fig. 3:**
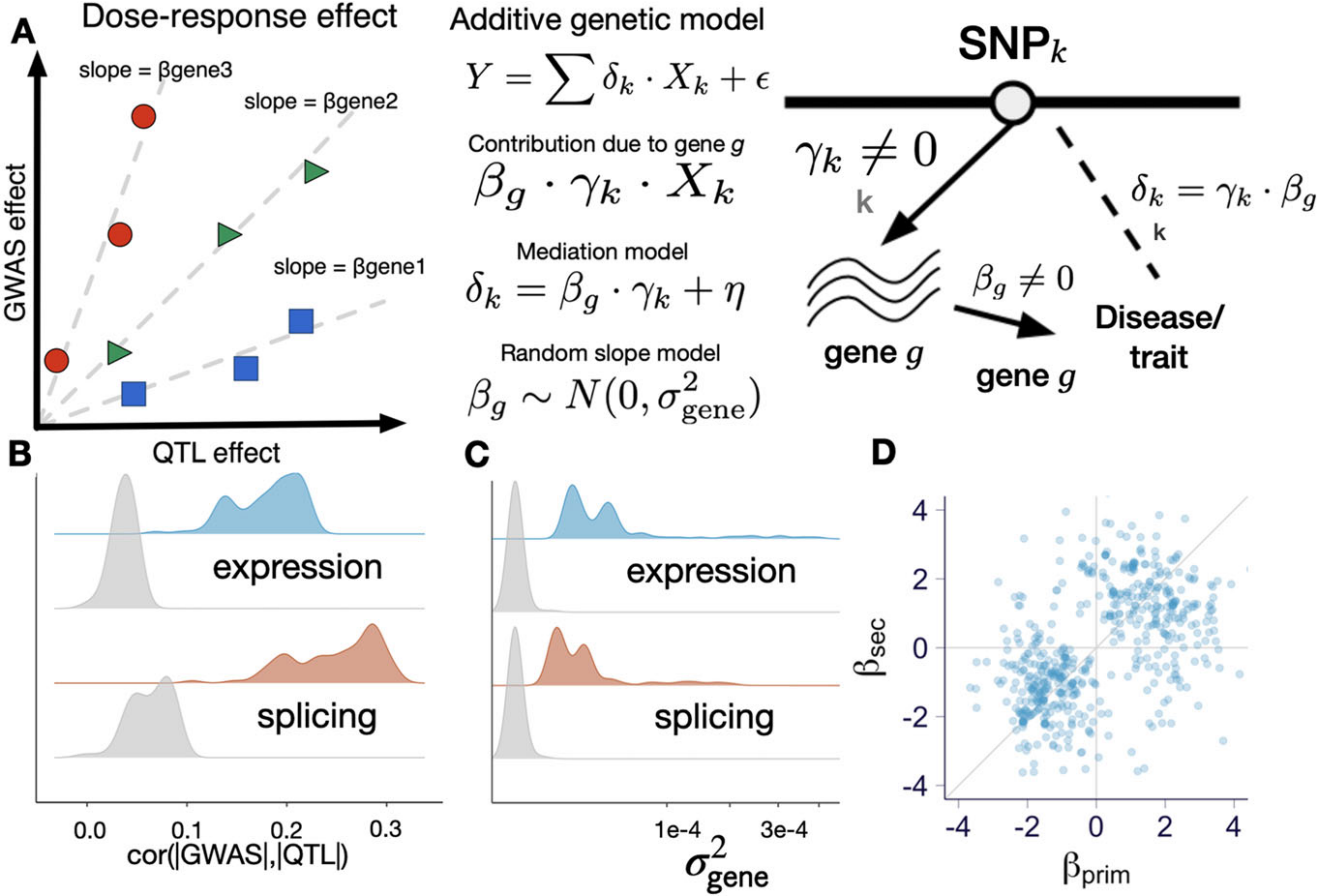
Dose-dependent effects of QTLs on complex traits. Here, all analyses were performed with fine-mapped variants (QTL with highest posterior inclusion probability). **a** Schematic representation of dose-response model. **b** Correlation between QTL and GWAS effects, $\text {Cor}(|\hat \delta |, |\hat \gamma |)$. Gray distribution represents permuted null with matched local LD. Each data point corresponds to the median correlation for the trait across 49 tissues. **c** Average mediated effects from mediation model ($\sigma ^{2}_{\text {gene}}$, median across tissues). Gray distribution represents permuted null with matched local LD. **e** Mediated effects of secondary vs. primary eQTLs of genes with colocalization probability (rcp) >0.10. in whole blood, genes for all 87 traits are shown

Nevertheless, enrichment studies can be confounded by many unknown factors. Therefore, we sought to gather stronger evidence for a causal link by testing whether there is a dose-dependent effect of expression and splicing QTLs on complex traits. Figure 3a illustrates schematically our approach. We examined whether expression or splicing associated variants (referred to as e/sVariants for the remainder of the paper) with higher impact on gene expression or splicing lead to higher impact on a complex trait, i.e., a larger GWAS effect (Fig. 3a). The impact of the regulation of a gene on a trait is quantified by the slope *β*_gene_. That is, a null hypothesis of no dose-dependent effect is equivalent to *β*_gene_=0.

To reduce unnecessary noise in the analysis, we included only the most likely causal e/sVariant within each credible set as determined by the e/sQTL fine-mapping (denoted “fine-mapped variants” throughout the remainder of the paper; see Methods on QTL fine-mapping).

First, we quantified dose-dependent effect of expression and splicing regulation on the trait as the average mediating effect size, $\bar \beta $. We calculated this average effect using the Pearson correlation between the absolute values of the molecular and complex trait effect sizes (cor(|*γ*|,|*δ*|)) across all fine-mapped variants (for any gene) for each trait-tissue pair. As hypothesized, we found, consistently across all tissue-trait pairs, a positive correlation between the GWAS and QTL effects, which was significantly larger than the permuted null with matched local LD. The average correlations were 0.18 (s.e. = 0.004, *p*<1×10^−30^) and 0.25 (s.e. = 0.006, *p*<1×10^−30^) for expression and splicing, respectively with the distribution of the median correlation across tissues for each trait shown in Fig. 3b. Averages and standard errors were calculated taking into account correlation between tissues, and *p* values were calculated against permuted null with matched local LD (Supplementary Text). The non-negative permuted correlation values indicate that local LD contributed to inflate the estimated mediation effect. These results provide the first line of evidence of the dose-response effect.

To test and account for mediation effect heterogeneity (different slope/dosage sensitivity for different genes), we modeled the gene-specific mediation effect, *β*_*g*_, as a random variable following a normal distribution $\beta _{g} \sim \mathcal {N}(0, \sigma _{\text {gene}}^{2})$. Under this random-effects model, the null hypothesis can be stated as $\sigma _{\text {gene}}^{2}=0$ (Supplementary Text; Fig. 3c). As shown in Fig. 3c, these effects were significantly larger than expected from the permuted null (expression *p*=1.8×10^−9^; splicing *p*=2.5×10^−7^). These results indicate that strong genetic effects on expression or splicing are more likely to have a strong association to complex traits, adding strong support to a dose-dependent relationship between gene regulation and downstream traits.

Importantly, by averaging across all genes, the estimates, from both the average and the random-effects approach, of the mediating effect are robust to confounding due to LD, as discussed in the Supplementary Text.

Another way to account for mediation effect heterogeneity is to make use of the allelic series of independent eQTLs identified for over half of the eGenes [9]. We examined whether the mediating effect (*β*=*δ*/*γ*) inferred from the primary eQTL (*β*_prim_) was consistent with the one inferred from the secondary eQTL (*β*_sec_). Among the independent eQTLs for a given gene, we called primary the one with the larger effect size. We considered only fine-mapped eQTLs given the low power to detect multiple independent sQTLs. We confirmed this concordance, as reported by the GTEx consortium [9], demonstrating that the correlation between the primary and secondary mediating effects is larger than expected given the LD between them. To better visualize this concordance, we plotted the estimated mediating effects of primary against the secondary eQTLs (whole blood shown here but other tissues look similar) in Fig. 3d and showed that they cluster in the first and third quadrants. All gene-trait pairs with relatively high regional colocalization probability (rcp > 0.10, see colocalization details below) are shown here to facilitate visualization, but the clustering around the diagonal line was observed even without the filtering. This provides a third confirmatory evidence for the widespread dose-dependent effects of eQTLs on complex traits.

Note that genes with discordant effects within the allelic series would be harder to detect and suggest more complex causal relationship or context specificity.

## Causal gene prediction and prioritization

**Fig. 4:**
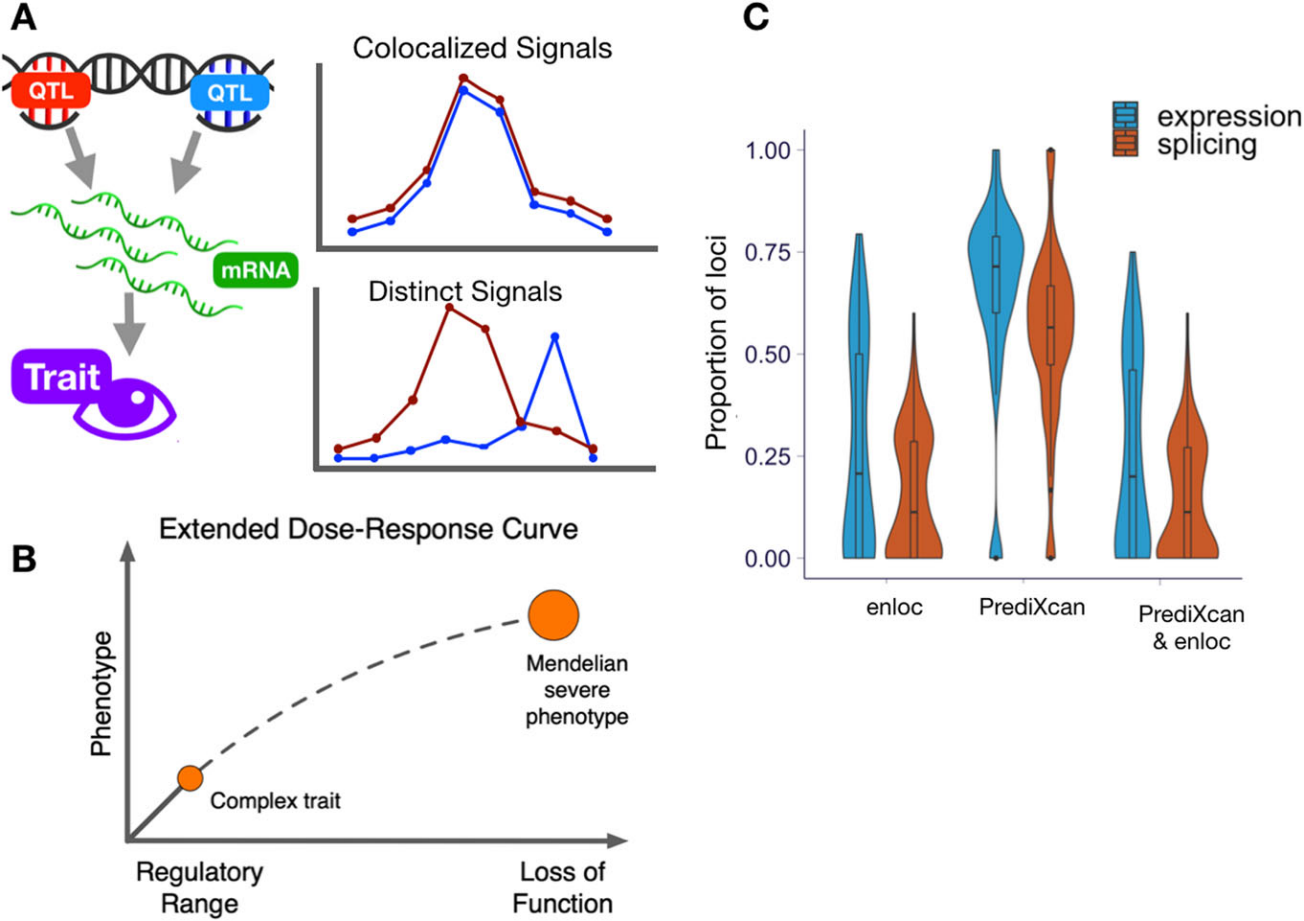
Identifying and validating predicted causal genes. **a** Schematic representation of association and colocalization approaches. **b** Schematic representation of extrapolating the dose-response curve to the Mendelian end of phenotypic variation spectrum [37]. **c** Proportion of GWAS-associated loci per trait that contain colocalized and PrediXcan-associated signals for expression and splicing

In addition to genome-wide analyses that shed light on the molecular architecture of complex traits, QTL analysis of GWAS data can identify potential causal genes and molecular changes in individual GWAS loci. Towards this end, we performed association analysis with genetically predicted regulation and colocalization (Fig. 4a). After evaluating the performance of *coloc* and *enloc* [16, 17], we chose *enloc* as our primary approach, due to its use of hierarchical models to estimate colocalization priors [16] and its ability to account for multiple causal variants. The *coloc* assumption of a single causal variant drastically reduces performance especially in large QTL datasets such as GTEx with widespread allelic heterogeneity. For a more extensive discussion on the benefits of Bayesian colocalization methods and comparison of *enloc* to other colocalization approaches including SMR-HEIDI, see [25]. We estimated the posterior regional colocalization probability (rcp), using *enloc*, for 12,072,964 tissue-gene-GWAS locus-trait tuples and 67,943,800 tissue-splicing event-GWAS locus-trait tuples. For the tally of colocalized genes, we used rcp > 0.5 as a stringent cutoff as demonstrated below with the low colocalization probabilities of height loci using two different datasets.

In total, we identified 3477 (15% of 23,963) unique genes colocalizing with GWAS hits (rcp > 0.5) across all traits and tissues analyzed. Similarly, 3157 splicing events (1% out of 310,042) colocalized with GWAS hits, corresponding to 1226 genes with at least one colocalized splicing event (5% of 23,963).

Colocalization of e/sQTLs with GWAS variants provides important causal support for molecular traits. However, we found their estimates to be overly conservative. To illustrate this point, we tested the colocalization of height with itself, using two large-scale studies of individuals of European-ancestry individuals: GIANT [26] and UK Biobank. We started by performing fine-mapping of both GWAS results using *susier* [27]. Notably, only 416 (39%) of GIANT’s fine-mapped credible sets overlapped with the corresponding UK Biobank credible sets. We estimated the colocalization probability as the sum of the product of posterior inclusion probabilities of variants for each of the 1069 independent credible sets in GIANT, which is similar to the approach used by eCAVIAR [15]. Two thirds of the GIANT credible sets (66.2%) had a colocalization probability below 0.01, and about half (48.9%) had a colocalization probability below 0.001. In other words, two thirds of the loci found by GIANT would be considered not to be colocalized with UK Biobank’s loci when using a seemingly very loose colocalization probability cutoff of 0.01. Given the larger sample size of the UK Biobank GWAS (*n* = 337,119 UKB GWAS vs. *n* = 253,288 for GIANT), the low colocalization cannot be attributed to lack of power. This result is likely due in part to the sensitivity to small LD differences between different EUR populations that make up large GWAS meta-analysis cohorts such as GIANT. Our analysis illustrates the fact that colocalization probability estimates are highly conservative and may miss many causal genes, and low colocalization probability should not be interpreted as evidence of lack of a causal link between the molecular phenotype and the GWAS trait. Notice that this limitation is not inherent to the colocalization method itself but the limitation of currently available large-scale GWAS meta-analysis results.

A complementary approach to colocalization is to estimate the GWAS trait association with genetically predicted gene expression or splicing [19]. The GTEx v8 data provides an important expansion of these analyses, allowing generation of prediction models in 49 tissues with whole genome sequencing data to impute gene expression and splicing variation. We trained prediction models using a variety of approaches and selected the top performing one based on precision, recall, and other metrics [28]. Briefly, the optimal model uses fine-mapping probabilities for feature selection and exploits global patterns of tissue sharing of regulation (Supplementary Text) to improve prediction. In-depth comparison of these fine-mapped models with Elastic Net-based and CTIMP [29] models is described in [28]. The analysis presented here uses these improved models (fine-mapped-*mashr*) instead of Elastic Net as reported in the main GTEx publication [9]. Multi-SNP prediction models were generated for a total of 686,241 gene-tissue and 1,816,703 splicing event-tissue pairs. The larger sample size and improved models led to an increase in the number of expression models to a median across tissues of 14,062, from a median of 4776 GTEx v7 Elastic Net models (median increase at 191%, Additional file 1: Fig. S5). Splicing models are available only for the v8 release.

Next, we computed the association between an imputed molecular phenotype (expression or splicing) and a trait to estimate the genic effect on the trait, using the summary statistics-based PrediXcan [24]. Given the widespread tissue sharing of regulatory variation [8], we also computed MultiXcan scores to integrate patterns of associations from multiple tissues and increase statistical power [10]. Out of the 22,518 genes tested with PrediXcan, 6407 (28%) showed a significant association with at least one of the 87 traits at Bonferroni-corrected *p* value threshold (*p*<0.05/686,241, where the denominator is the number of gene-tissue pairs tested; Additional file 1: Fig. S6). For splicing, about 15% (20,364 of 138,890) of tested splicing events showed a significant association (*p*<0.05/1,816,703, where the denominator is the number of intron-tissue pairs tested). Nearly all traits (94%; 82 out of 87) showed at least one significant gene-level PrediXcan association in at least one tissue (Additional file 1: Figs. S7 and S8); the median number of associated genes across traits was 974. This resource of PrediXcan associations can be used to prioritize a list of putatively causal genes for follow-up studies.

To replicate the PrediXcan expression associations in an independent dataset, BioVU, which is a large-scale biobank tied to Electronic Health Records [30, 31], we selected seven traits with predicted high statistical power. Out of 947 gene-tissue-trait discoveries tested, 458 unique gene-tissue-trait triplets (48%) showed replication in this independent biobank (PrediXcan association *p*<0.05; see Supplementary Text). Further confirming this statistical replication in BioVU, we used the PheWAS [32] catalog as the silver standard and found an AUC curve of 0.62. [33].

Altogether, these results provide abundant links between gene regulation and GWAS loci. To further quantify this, we split the genome into approximately LD-independent blocks [34] and identified blocks with a significant GWAS variant for each trait (at Bonferroni threshold adjusted for number of variants 0.05/8.8×10^6^∼5.7×10^−9^); we refer to any such region-trait pair by “GWAS locus.” We calculated the proportion of GWAS loci that contain a significantly associated gene via PrediXcan or a colocalized gene via *enloc* (rcp > 0.5). Briefly, the LD blocks are defined by analyzing empirical patterns of LD observed in 1000 Genomes [35] and variants in different regions are unlikely to be correlated, thus providing us with a data-driven criterion to distinguish independent genomic signals.

Across the traits, 72% (3899/5385) of GWAS loci had a PrediXcan expression association in the same LD block, of which 55% (2125/3899) had evidence of colocalization with an eQTL; for splicing, 62% (3345/5385) had a PrediXcan association of which 34% (1135/3345) colocalized with an sQTL (Additional file 1: Table S3). From the combined list of eGenes and sGenes, 47% of loci have a gene with both *enloc* and PrediXcan support. The distribution of the proportion of associated and colocalized GWAS loci across 87 traits is summarized in Fig. 4c; for a typical complex trait, about 20% of GWAS loci contained a colocalized, significantly associated gene while 11% contained a colocalized, significantly associated splicing event. These results propose function for a large number of GWAS loci, but most loci remain without candidate genes, highlighting the need to expand the resolution of transcriptome studies.

A recent report estimates that the proportion of trait variance explained by the assayed transcriptome is on average 11% [36]. Even though this number is not directly comparable with the proportion of loci with support from PrediXcan and *enloc*, some discussion is warranted. Differences may arise with our analysis from the fact that (1) GTEx v8 doubles the number of samples with both genotype and RNA-seq relative to v7, (2) we include links based on splicing in addition to expression, (3) a variant may act through both regulation of expression levels and other undetected mechanisms (pleiotropy), and (4) attenuation bias may reduce the estimates given the error in eQTL effect sizes.

Of note, two members of the sterolin family, *ABCG5* and *ABCG8*, showed highly significant predicted causal associations using both PrediXcan and *enloc* for LDL-C levels and self-reported high cholesterol levels. *ABCG8* showed more significant associations in both datasets (chr2: 43838964–43878466; UKB self-reported high cholesterol: −log10(*p*_PrediXcan_) = 38.43, rcp = 0.985; GLGC LDL-C: −log10(*p*_PrediXcan_) = 71.40, rcp = 0.789), compared to *ABCG5* (chr2: 43812472–43838865; −log10(*p*_PrediXcan_) = 36.85, rcp = 0.941; −log10(*p*_PrediXcan_) = 80.80, rcp = 0.705). Mutations in either of the two ATP-binding cassette (ABC) half-transporters, *ABCG5* and *ABCG8*, lead to reduced secretion of sterols into bile and, ultimately, obstruct cholesterol and other sterols exiting the body [38]. In mice with disrupted *Abcg5* and *Abcg8* (G5G8-/-), a 2- to 3-fold increase in the fractional absorption of dietary plan sterols and extremely low biliary cholesterol levels was observed, indicating that disrupting these genes contributes greatly to plasma cholesterol levels [39]. The overexpression of human *ABCG5* and *ABCG8* in transgenic Ldlr-/- mice resulted in 30% reduction in hepatic cholesterol levels and 70% reduced atherosclerotic legion in the aortic root and arch [40] after 6 months on a Western diet.

Several other lipid-associated loci were also consistently predicted as causal across OMIM, the rare variant derived set, PrediXcan and *enloc*. Rare protein-truncating variants in *APOB* have been previously associated with reduced LDL-C and triglyceride levels and reduced coronary heart disease risk [41]. Interestingly, *APOB* has been predicted as a causal gene in four related traits, coronary artery disease, LDL-C levels, triglyceride levels, and self-reported high cholesterol levels. Among the four traits, PrediXcan showed the highest association to LDL-C levels (−log10(*p*_PrediXcan_) = 130.89; rcp = 0.485) while self-reported high cholesterol showed the strongest evidence using *enloc* at nearly maximum posterior probability (−log10(*p*_PrediXcan_) = 93.66; rcp = 0.969). Although *APOB* has been suggested as a better molecular indicator of predicted cardiac events in place of LDL-C levels [42, 43], its translation has been surprisingly slow in clinical practice [44]. Here, we provide an additional support for the crucial role *APOB* may play in predicting lipid traits.

## Performance for identifying “ground truth” genes

To compare the ability of different approaches to identify the causal gene that mediates the association between GWAS loci and the traits, we sought to curate sets of “ground truth” genes using information that is independent of GWAS results (Additional file 1: Fig. S9). We call these sets “silver standards” as a reminder of their imperfect nature. The first silver standard was based on the OMIM (Online Mendelian Inheritance in Man) database [45], and the second one was based on publicly available rare variant tests from exome-wide association studies [46–48], resulting in 1592 OMIM gene-trait pairs and 101 rare variant-based gene-trait pairs (Additional file 3: Table S4, Additional file 4: Table S5).

The rationale behind the choice of the OMIM database is the comorbidity among Mendelian and complex diseases suggesting that genes whose loss of function cause Mendelian diseases also manifest in milder phenotypic variation when modified to a lesser degree by regulatory variation [49, 50]. In other words, that the dose-response curve at the regulatory range may be extrapolated to the rare, loss-of-function end (Fig. 4b). The rationale behind the use of the rare variant association study results is the excess of deleterious rare variants associated with complex traits in genes that are in the vicinity of common variants associated with the same trait [46, 51, 52]. Note that rare variant associations are nearly independent of common variants due to the allele frequency difference between them.

For the analysis, we partitioned the genome into approximately independent LD blocks [34] and considered all the blocks where a silver standard gene was available for the trait. Since only genes in the vicinity of an index gene can be discovered with cis-regulatory information, we only considered the LD blocks with a GWAS significant variant (Additional file 1: Fig. S10). This selection resulted in 228 OMIM gene-trait pairs (28 distinct traits) and 80 rare variant-associated gene-trait pairs (5 distinct traits) that are located within the same LD block as the GWAS locus for a matched trait.

**Fig. 5:**
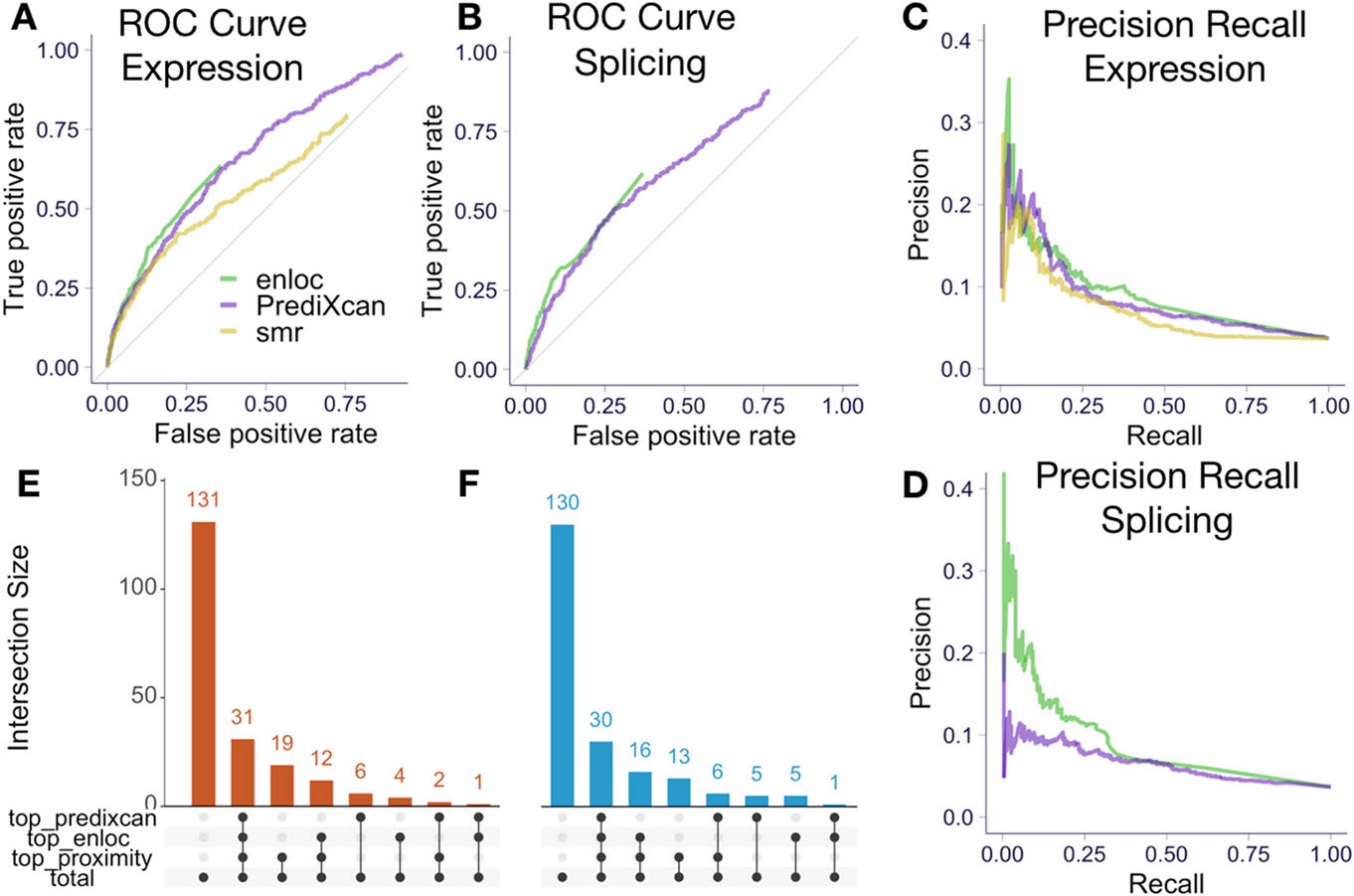
Causal gene identification performance. ROC curves of *enloc* and PrediXcan statistics to identify the “causal” genes (OMIM silver standard) using expression (**a**) and splicing (**b**) are shown. Precision recall curves of *enloc* and PrediXcan to identify silver standard genes using expression (**c**) and splicing (**d**) (we show the precision in the range 0 to 0.4 to improve visualization). The number of GWAS loci (LD block-trait pairs) where the OMIM gene was ranked at the top by proximity, *enloc*, and PrediXcan using expression (**e**) and splicing (**f**). In 131 loci out of 206, the OMIM gene was not ranked at the top by either proximity, significance, or colocalization. In thirty one of the loci, the OMIM gene was ranked first by all three criteria. In nineteen loci, the OMIM gene was closest gene (to the top GWAS variant) but not the top gene by PrediXcan significance nor *enloc*’s colocalization probability

Both PrediXcan and *enloc* based on expression and splicing showed good sensitivity and specificity for identifying the silver standard genes as demonstrated by the ROC curves in Fig. 5a, b. These are well above the gray random guess lines indicating the predictive ability of these methods to find causal genes (see comparison with permuted null in Additional file 1: Fig. S11).

For applications such as target selection for drug development or follow-up experiments, another relevant metric is the precision or, equivalently, positive predictive value (PPV)—the probability that the gene-trait link is causal given that it is called significant or colocalized. Precision recall curves for expression- and splicing-based predictions are shown in Fig. 5c, d. With more stringent threshold (towards the left in the recall axis), higher precision is obtained.

For example, 8.7% of genes with PrediXcan significant genes (*p*<0.05/49 × number of gene/trait pairs) were OMIM genes and 14.8% of genes with high colocalization probability (rcp > 0.5) were also OMIM genes for matched traits.

Multiple factors contribute to the rather low precision. One of them is the widespread molecular pleiotropy [9], i.e., multiple genes affected by the same trait-associated variants. Another factor reducing the overall causal gene detection performance is the inherent bias of the OMIM gene list. Our current understanding of gene function is biased towards protein-coding variants with very large effects, as reflected in the list of OMIM genes. Genes associated to rare severe disease tend to be depleted of regulatory variation [53, 54], which will decrease the performance of a QTL-based method [54].

Among the 206 loci with at least one OMIM gene (a few loci contained multiple OMIM genes), an OMIM gene was the closest to the top GWAS SNP in 31.6% of the loci, it was the most colocalized in 24.8% of the loci, and it was the most significant in 20.4% of the loci (Fig. 5e, f).

To further investigate whether this predictive power could be improved by combining multiple criteria, we performed a joint logistic regression of OMIM gene status on (1) the proximity of the top GWAS variant to the nearest gene (distance to the gene body), (2) posterior probability of colocalization, and (3) PrediXcan association significance between QTL and GWAS variants. To make the scale of the three features more comparable, we used their respective ranking. When genes did not have an enloc or PrediXcan score, they were assigned to the last position in the ranking. All three features were significant predictors of OMIM gene status, with better ranked genes more likely to be OMIM genes (proximity *p*=2.0×10^−2^, *enloc* *p*=6.1×10^−3^, PrediXcan *p*=2.5×10^−4^), indicating that each method provides an additional source of causal evidence even after conditioning on the others. Similar results were obtained using splicing colocalization and association scores and the rare variant-based silver standard, as shown in Additional file 1: Table S6. These results provide further empirical evidence that a combination of colocalization and association methods will perform better than individual ones. The significance of the proximity score even after accounting for significance and colocalization indicates missing regulatory events, i.e., mechanisms that may be uncovered by assaying other tissue or cell type contexts, larger samples, and other molecular traits, underscoring the need to expand the size and breadth of QTL studies. Proximity criterion also helps resolve cases when QTL data indicates multiple genes with similar significance.

Predicted OMIM genes included well-known findings such as *PCSK9* for LDLR, with *PCSK9* significant and colocalized for relevant GWAS traits (LDL-C levels, coronary artery disease, and self-reported high cholesterol), and *Interleukins* and *HLA* subunits for asthma, both significant and colocalized for related immunological traits. Significantly associated and colocalized genes that predicted OMIM genes also included *FLG* (eczema), *TPO* (hypothyroidism), and *NOD2* (inflammatory bowel disease) (see Additional file 1: Table S4 for complete list). Analysis with rare variant-based silver standard yielded similar conclusions (Supplementary Text; Additional file 1: Fig. S12).

## Tissue enrichment of GWAS signals

The broad sharing of regulatory variation across tissues and the reduced significance of tissue-specific eQTLs make causal tissue identification challenging. To address this problem, we devised a novel approach to identify tissues of relevance for the etiology of complex traits. We investigated the patterns of tissue specificity and tissue sharing of PrediXcan association results across 49 tissues. For each trait-gene pair, the PrediXcan *z*-score can be represented as a 49×1 vector with each entry being the gene-level *z*-score in the corresponding tissue (if the prediction model of the gene is not available in that tissue, we filled in zero). To explore the tissue specificity of the PrediXcan *z*-score vector, we proceeded by assigning the *z*-score vector to a tissue-pattern category and tested whether certain tissue-pattern categories were over-represented among colocalized PrediXcan genes as compared to non-colocalized genes. We used the FLASH factors identified from matrix factorization applied to the cis-eQTL effect size matrix, as PrediXcan and cis-eQTL shared similar tissue-sharing pattern (Supplementary Text). To obtain a set of detailed and biologically interpretable tissue-pattern categories from the 31 FLASH factors, we manually merged them into 18 categories as shown in Additional file 1: Fig. S13. For each trait, we projected the *z*-score vector of each gene to one of the 31 FLASH factors (as described in Section 9 of Additional file 1) so that the gene was assigned to the corresponding tissue-pattern category. We defined a “positive” set of genes as the ones with PrediXcan *p* value that meets Bonferroni significance at *α*=0.05 in at least one tissue and *enloc* rcp > 0.01 in at least one tissue, which could be thought as a set of candidate genes affecting the trait through expression level. We chose a rather low threshold used for the rcp due to the stringent conservative nature of colocalization probabilities. We also constructed a “negative” set of genes with *enloc* rcp = 0, which could be thought as a set of genes whose expressions were unlikely to affect the trait. We proceeded to test whether certain tissue-pattern categories were enriched in “positive” set as compared to “negative” set. Since the main focus of this analysis was tissue-specific patterns, we excluded *Factor1* (the cross-tissue factor) and *Factor25* (likely to be a tissue-shared factor capturing tissues with large sample size). Additionally, we excluded *Factor7* (testis), as it was unlikely to be the mediating tissue but might introduce false positives. We tested the enrichment of each tissue-pattern category by Fisher’s exact test (“positive”/“negative” sets and in/not in tissue-pattern category). Among 87 traits, 82 traits had *enloc* signal and the enrichment of these was calculated accordingly.

**Fig. 6:**
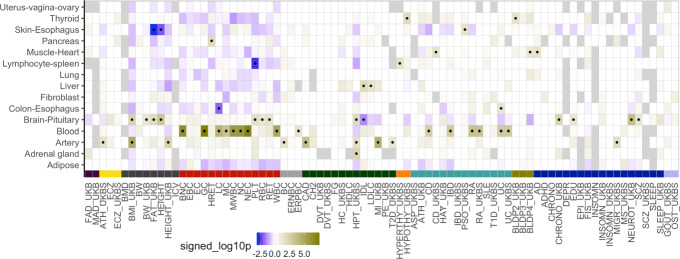
Identifying trait-relevant tissues using tissue-specific enrichment. Enrichment of tissue-specific association and colocalization compared to the pattern of tissue specificity of non-colocalized genes. Over-representation of the tissue class for PrediXcan-significant and colocalized genes is indicated by dark yellow while depletion is indicated by blue. Black dots label the tissue class-trait pairs passing the nominal *p* value significance threshold of 0.05. Abbreviation: Table S2. Trait category colors: Fig. S4

Using the pattern of tissue classes of non-colocalized genes (rcp = 0) as the expected null, we assessed whether significantly associated and colocalized genes (PrediXcan significant and rcp > 0.01) were over-represented in certain tissue classes (Fig. 6). Consistent with previous reports [11, 55], we identified several instances in which the most significant tissue is supported by current biological knowledge. For example, blood cell count traits were enriched in whole blood, neuroticism and fluid intelligence in brain/pituitary, hypothyrodism in thyroid, coronary artery disease in artery, and cholesterol-related traits in liver. Taken together, these results show the potential of leveraging regulatory variation to help identify tissues of relevance for complex traits.

## Discussion

We performed in-depth examination of the phenotypic consequences of the genetic regulation of the transcriptome and provide data-driven analytical approaches to benchmark methods that assign function to GWAS loci and best-practice guidelines for using the GTEx resources to interpret GWAS results. We provide a systematic empirical demonstration of the widespread dose-dependent effect of expression and splicing on complex traits, i.e., variants with larger impact at the molecular level have larger impact at the trait level. Furthermore, we found that target genes in GWAS loci identified by *enloc* and PrediXcan were predictive of OMIM genes for matched traits, implying that for a proportion of the genes, the dose-response curve can be extrapolated to the rare and more severe end of the genotype-trait spectrum. The observation that common regulatory variants target genes also implicated by rare coding variants underscores the extent to which these different types of genetic variants converge to mediate a spectrum of similar pathophysiological effects and may provide a powerful approach to drug target discovery.

We implemented association and colocalization methods that leverage the observed allelic heterogeneity of expression traits. After extensive comparison using two independent sets of silver standard gene-trait pairs, we conclude that combining *enloc*, PrediXcan, and proximity ranking outperforms the individual approaches. The significance of the proximity ranking is a sign of the “missing regulability” emphasizing the need to expand the resolution, sample size, and range of contexts of transcriptome studies as well as to examine other molecular mechanisms.

We caution that the increased power offered by this release of the GTEx resources also brings higher risk of false links due to LD contamination and that naive use of eQTL or sQTL association *p* values to assign function to a GWAS locus can be misleading. Colocalization approaches can be used to weed out LD contamination, but given the lack of LD references from source studies, they can also be overtly conservative. General purpose reference LD from publicly available sources are not sufficient for fine-mapping and colocalization approaches, which can be highly sensitive to LD misspecification when only summary results are used [56]. The GWAS community has made great progress in recognizing the need to share summary results, but to take full advantage of these data, improved sharing of LD information from the source study as well as from large sequencing reference datasets is also required.

Finally, we generated several resources that can open the door for addressing key questions in complex trait genomics. We present a catalog of gene-level associations, including potential target genes for nearly half of the GWAS loci investigated here that provides a rich basis for studies on the functional mechanisms of complex diseases and traits. We provide a database of optimal gene expression imputation models that were built on the fine-mapping probabilities for feature selection and that leverage the global patterns of tissue sharing of regulation to improve the weights. These imputation models of expression and splicing, which to date has been challenging to study, provide a foundation for transcriptome-wide association studies of the human phenome—the collection of all human diseases and traits—to further accelerate discovery of trait-associated genes. Collectively, these data thus represent a valuable resource, enabling novel biological insights and facilitating follow-up studies of causal mechanisms.

## Authors

^∗^ alphabetic order **Lead Analysts**^**∗**^*Equal contribution* Alvaro N Barbeira, Rodrigo Bonazzola, Eric R Gamazon, Yanyu Liang, YoSon Park

**Analysts**^**∗**^ François Aguet, Lisa Bastarache, Ron Do, Gao Wang, Andrew R Hamel, Farhad Hormozdiari, Zhuoxun Jiang, Daniel Jordan, Sarah Kim-Hellmuth, Boxiang Liu, Milton D Pividori, Abhiram Rao, Marie Verbanck, Dan Zhou

**GTEx GWAS Working Group**^**∗**^ François Aguet, Kristin Ardlie, Alvaro N Barbeira, Rodrigo Bonazzola, Christopher D Brown, Lin Chen, Eric R Gamazon, Kevin Gleason, Andrew R Hamel, Farhad Hormozdiari, Hae Kyung Im, Sarah Kim-Hellmuth, Tuuli Lappalainen, Yanyu Liang, Boxiang Liu, Dan L Nicolae, Yoson Park, Milton D Pividori, Abhiram Rao, John M. Rouhana, Ayellet V Segrè, Xiaoquan Wen

**Senior Leadership**^**∗**^ Kristin Ardlie, Christopher D. Brown, Hae Kyung Im, Tuuli Lappalainen, Mark McCarthy, Stephen Montgomery, Ayellet V Segrè, Matthew Stephens, Xiaoquan Wen

**Manuscript Writing Group**^**∗**^ Eric R Gamazon, Hae Kyung Im, Tuuli Lappalainen, Yanyu Liang, YoSon Park

**Corresponding Author**^**∗**^ Hae Kyung Im

## GTEx Consortium

**Laboratory and Data Analysis Coordinating Center (LDACC):** François Aguet^1^, Shankara Anand^1^, Kristin G Ardlie^1^, Stacey Gabriel^1^, Gad Getz^1,2^, Aaron Graubert^1^, Kane Hadley^1^, Robert E Handsaker^3,4,5^, Katherine H Huang^1^, Seva Kashin^3,4,5^, Xiao Li^1^, Daniel G MacArthur^4,6^, Samuel R Meier^1^, Jared L Nedzel^1^, Duyen Y Nguyen^1^, Ayellet V Segrè^1,7^, Ellen Todres^1^

**Analysis Working Group (funded by GTEx project grants):** François Aguet^1^, Shankara Anand^1^, Kristin G Ardlie^1^, Brunilda Balliu^8^, Alvaro N Barbeira^9^, Alexis Battle^10,11^, Rodrigo Bonazzola^9^, Andrew Brown^12,13^, Christopher D Brown^14^, Stephane E Castel^15,16^, Don Conrad^17,18^, Daniel J Cotter^19^, Nancy Cox^20^, Sayantan Das^21^, Olivia M de Goede^19^, Emmanouil T Dermitzakis^12,22,23^, Barbara E Engelhardt^24,25^, Eleazar Eskin^26^, Tiffany Y Eulalio^27^, Nicole M Ferraro^27^, Elise Flynn^15,16^, Laure Fresard^28^, Eric R Gamazon^29,30,31,20^, Diego Garrido-Martín^32^, Nicole R Gay^19^, Gad Getz^1,2^, Aaron Graubert^1^, Roderic Guigó^32,33^, Kane Hadley^1^, Andrew R Hamel^7,1^, Robert E Handsaker^3,4,5^, Yuan He^10^, Paul J Hoffman^15^, Farhad Hormozdiari^34,1^, Lei Hou^35,1^, Katherine H Huang^1^, Hae Kyung Im^9^, Brian Jo^24,25^, Silva Kasela^15,16^, Seva Kashin^3,4,5^, Manolis Kellis^35,1^, Sarah Kim-Hellmuth^15,16,36^, Alan Kwong^21^, Tuuli Lappalainen^15,16^, Xiao Li^1^, Xin Li^28^, Yanyu Liang^9^, Daniel G MacArthur^4,6^, Serghei Mangul^26,37^, Samuel R Meier^1^, Pejman Mohammadi^15,16,38,39^, Stephen B Montgomery^28,19^, Manuel Muñoz-Aguirre^32,40^, Daniel C Nachun^28^, Jared L Nedzel^1^, Duyen Y Nguyen^1^, Andrew B Nobel^41^, Meritxell Oliva^9,42^, YoSon Park^14,43^, Yongjin Park^35,1^, Princy Parsana^11^, Ferran Reverter^44^, John M Rouhana^7,1^, Chiara Sabatti^45^, Ashis Saha^11^, Ayellet V Segrè^1,7^, Andrew D Skol^9,46^, Matthew Stephens^47^, Barbara E Stranger^9,48^, Benjamin J Strober^10^, Nicole A Teran^28^, Ellen Todres^1^, Ana Viñuela^49,12,22,23^, Gao Wang^47^, Xiaoquan Wen^21^, Fred Wright^50^, Valentin Wucher^32^, Yuxin Zou^51^

**Analysis Working Group (not funded by GTEx project grants):** Pedro G Ferreira^52,53,54^, Gen Li^55^, Marta Melé^56^, Esti Yeger-Lotem^57,58^

**Leidos Biomedical - Project Management:** Mary E Barcus^59^, Debra Bradbury^60^, Tanya Krubit^60^, Jeffrey A McLean^60^, Liqun Qi^60^, Karna Robinson^60^, Nancy V Roche^60^, Anna M Smith^60^, Leslie Sobin^60^, David E Tabor^60^, Anita Undale^60^

**Biospecimen collection source sites:** Jason Bridge^61^, Lori E Brigham^62^, Barbara A Foster^63^, Bryan M Gillard^63^, Richard Hasz^64^, Marcus Hunter^65^, Christopher Johns^66^, Mark Johnson^67^, Ellen Karasik^63^, Gene Kopen^68^, William F Leinweber^68^, Alisa McDonald^68^, Michael T Moser^63^, Kevin Myer^65^, Kimberley D Ramsey^63^, Brian Roe^65^, Saboor Shad^68^, Jeffrey A Thomas^68,67^, Gary Walters^67^, Michael Washington^67^, Joseph Wheeler^66^

**Biospecimen core resource:** Scott D Jewell^69^, Daniel C Rohrer^69^, Dana R Valley^69^

**Brain bank repository:** David A Davis^70^, Deborah C Mash^70^

**Pathology** Mary E Barcus^59^, Philip A Branton^71^, Leslie Sobin^60^

**ELSI study:** Laura K Barker^72^, Heather M Gardiner^72^, Maghboeba Mosavel^73^, Laura A Siminoff^72^

**Genome Browser Data Integration & Visualization:** Paul Flicek^74^, Maximilian Haeussler^75^, Thomas Juettemann^74^, W James Kentv^75^, Christopher M Lee^75^, Conner C Powell^75^, Kate R Rosenbloom^75^, Magali Ruffier^74^, Dan Sheppard^74^, Kieron Taylor^74^, Stephen J Trevanion^74^, Daniel R Zerbino^74^

**eGTEx groups:** Nathan S Abell^19^, Joshua Akey^76^, Lin Chen^42^, Kathryn Demanelis^42^, Jennifer A Doherty^77^, Andrew P Feinberg^78^, Kasper D Hansen^79^, Peter F Hickey^80^, Lei Hou^35,1^, Farzana Jasmine^42^, Lihua Jiang^19^, Rajinder Kaul^81,82^, Manolis Kellis^35,1^, Muhammad G Kibriya^42^, Jin Billy Li^19^, Qin Li^19^, Shin Lin^83^, Sandra E Linder^19^, Stephen B Montgomery^28,19^, Meritxell Oliva^9,42^, Yongjin Park^35,1^, Brandon L Pierce^42^, Lindsay F Rizzardi^84^, Andrew D Skol^9,46^, Kevin S Smith^28^, Michael Snyder^19^, John Stamatoyannopoulos^81,85^, Barbara E Stranger^9,48^, Hua Tang^19^, Meng Wang^19^

**NIH program management:** Philip A Branton^71^, Latarsha J Carithers^71,86^, Ping Guan^71^, Susan E Koester^87^, A. Roger Little^88^, Helen M Moore^71^, Concepcion R Nierras^89^, Abhi K Rao^71^, Jimmie B Vaught^71^, Simona Volpi^90^

**Affiliations**

1. The Broad Institute of MIT and Harvard, Cambridge, MA, USA 2. Cancer Center and Department of Pathology, Massachusetts General Hospital, Boston, MA, USA 3. Department of Genetics, Harvard Medical School, Boston, MA, USA 4. Program in Medical and Population Genetics, The Broad Institute of Massachusetts Institute of Technology and Harvard University, Cambridge, MA, USA 5. Stanley Center for Psychiatric Research, Broad Institute, Cambridge, MA, USA 6. Analytic and Translational Genetics Unit, Massachusetts General Hospital, Boston, MA, USA 7. Ocular Genomics Institute, Massachusetts Eye and Ear, Harvard Medical School, Boston, MA, USA 8. Department of Biomathematics, University of California, Los Angeles, Los Angeles, CA, USA 9. Section of Genetic Medicine, Department of Medicine, The University of Chicago, Chicago, IL, USA v 10. Department of Biomedical Engineering, Johns Hopkins University, Baltimore, MD, USA 11. Department of Computer Science, Johns Hopkins University, Baltimore, MD, USA 12. Department of Genetic Medicine and Development, University of Geneva Medical School, Geneva, Switzerland 13. Population Health and Genomics, University of Dundee, Dundee, Scotland, UK 14. Department of Genetics, University of Pennsylvania, Perelman School of Medicine, Philadelphia, PA, USA 15. New York Genome Center, New York, NY, USA 16. Department of Systems Biology, Columbia University, New York, NY, USA 17. Department of Genetics, Washington University School of Medicine, St. Louis, MO, USA 18. Department of Pathology & Immunology, Washington University School of Medicine, St. Louis, MO, USA 19. Department of Genetics, Stanford University, Stanford, CA, USA 20. Division of Genetic Medicine, Department of Medicine, Vanderbilt University Medical Center, Nashville, TN, USA 21. Department of Biostatistics, University of Michigan, Ann Arbor, MI, USA 22. Institute for Genetics and Genomics in Geneva (iGE3), University of Geneva, Geneva, Switzerland 23. Swiss Institute of Bioinformatics, Geneva, Switzerland 24. Department of Computer Science, Princeton University, Princeton, NJ, USA 25. Center for Statistics and Machine Learning, Princeton University, Princeton, NJ, USA 26. Department of Computer Science, University of California, Los Angeles, Los Angeles, CA, USA 27. Program in Biomedical Informatics, Stanford University School of Medicine, Stanford, CA, USA 28. Department of Pathology, Stanford University, Stanford, CA, USA 29. Data Science Institute, Vanderbilt University, Nashville, TN, USA 30. Clare Hall, University of Cambridge, Cambridge, UK 31. MRC Epidemiology Unit, University of Cambridge, Cambridge, UK 32. Centre for Genomic Regulation (CRG), The Barcelona Institute for Science and Technology, Barcelona, Catalonia, Spain 33. Universitat Pompeu Fabra (UPF), Barcelona, Catalonia, Spain 34. Department of Epidemiology, Harvard T.H. Chan School of Public Health, Boston, MA, USA 35. Computer Science and Artificial Intelligence Laboratory, Massachusetts Institute of Technology, Cambridge, MA, USA 36. Statistical Genetics, Max Planck Institute of Psychiatry, Munich, Germany 37. Department of Clinical Pharmacy, School of Pharmacy, University of Southern California, Los Angeles, CA, USA 38. Scripps Research Translational Institute, La Jolla, CA, USA 39. Department of Integrative Structural and Computational Biology, The Scripps Research Institute, La Jolla, CA, USA 40. Department of Statistics and Operations Research, Universitat Politècnica de Catalunya (UPC), Barcelona, Catalonia, Spain 41. Department of Statistics and Operations Research and Department of Biostatistics, University of North Carolina, Chapel Hill, NC, USA 42. Department of Public Health Sciences, The University of Chicago, Chicago, IL, USA 43. Department of Systems Pharmacology and Translational Therapeutics, University of Pennsylvania, Perelman School of Medicine, Philadelphia, PA, USA 44. Department of Genetics, Microbiology and Statistics, University of Barcelona, Barcelona, Spain 45. Departments of Biomedical Data Science and Statistics, Stanford University, Stanford, CA, USA 46. Department of Pathology and Laboratory Medicine, Ann & Robert H. Lurie Children’s Hospital of Chicago, Chicago, IL, USA 47. Department of Human Genetics, University of Chicago, Chicago, IL, USA 48. Center for Genetic Medicine, Department of Pharmacology, Northwestern University, Feinberg School of Medicine, Chicago, IL, USA 49. Department of Twin Research and Genetic Epidemiology, King’s College London, London, UK 50. Bioinformatics Research Center and Departments of Statistics and Biological Sciences, North Carolina State University, Raleigh, NC, USA 51. Department of Statistics, University of Chicago, Chicago, IL, USA 52. Department of Computer Sciences, Faculty of Sciences, University of Porto, Porto, Portugal 53. Instituto de Investigação e Inovação em Sauúde, Universidade do Porto, Porto, Portugal 54. Institute of Molecular Pathology and Immunology, University of Porto, Porto, Portugal 55. Columbia University Mailman School of Public Health, New York, NY, USA 56. Life Sciences Department, Barcelona Supercomputing Center, Barcelona, Spain 57. Department of Clinical Biochemistry and Pharmacology, Ben-Gurion University of the Negev, Beer-Sheva, Israel 58 National Institute for Biotechnology in the Negev, Beer-Sheva, Israel 59. Leidos Biomedical, Frederick, MD, USA 60. Leidos Biomedical, Rockville, MD, USA 61. UNYTS, Buffalo, NY, USA 62. Washington Regional Transplant Community, Annandale, VA, USA 63. Therapeutics, Roswell Park Comprehensive Cancer Center, Buffalo, NY, USA 64. Gift of Life Donor Program, Philadelphia, PA, USA 65. LifeGift, Houston, TX, USA 66. Center for Organ Recovery and Education, Pittsburgh, PA, USA 67. LifeNet Health, Virginia Beach, VA. USA v 68. National Disease Research Interchange, Philadelphia, PA, USA v 69. Van Andel Research Institute, Grand Rapids, MI, USA 70. Department of Neurology, University of Miami Miller School of Medicine, Miami, FL, USA 71. Biorepositories and Biospecimen Research Branch, Division of Cancer Treatment and Diagnosis, National Cancer Institute, Bethesda, MD, USA 72. Temple University, Philadelphia, PA, USA 73. Virgina Commonwealth University, Richmond, VA, USA 74. European Molecular Biology Laboratory, European Bioinformatics Institute, Hinxton, UK v 75. Genomics Institute, UC Santa Cruz, Santa Cruz, CA, USA 76. Carl Icahn Laboratory, Princeton University, Princeton, NJ, USA 77. Department of Population Health Sciences, The University of Utah, Salt Lake City, UT, USA 78. Schools of Medicine, Engineering, and Public Health, Johns Hopkins University, Baltimore, MD, USA 79. Department of Biostatistics, Bloomberg School of Public Health, Johns Hopkins University, Baltimore, MD, USA 80. Department of Medical Biology, The Walter and Eliza Hall Institute of Medical Research, Parkville, Victoria, Australia 81. Altius Institute for Biomedical Sciences, Seattle, WA, USA v 82. Division of Genetics, University of Washington, Seattle, WA, USA 83. Department of Cardiology, University of Washington, Seattle, WA, USA 84. HudsonAlpha Institute for Biotechnology, Huntsville, AL, USA 85. Genome Sciences, University of Washington, Seattle, WA, USA 86. National Institute of Dental and Craniofacial Research, Bethesda, MD, USA 87. Division of Neuroscience and Basic Behavioral Science, National Institute of Mental Health, National Institutes of Health, Bethesda, MD, USA 88. National Institute on Drug Abuse, Bethesda, MD, USA 89. Office of Strategic Coordination, Division of Program Coordination, Planning and Strategic Initiatives, Office of the Director, National Institutes of Health, Rockville, MD, USA 90. Division of Genomic Medicine, National Human Genome Research Institute, Bethesda, MD, USA

## Supplementary Information

**Additional file 1** Supplementary Materials including detailed methods, tables, and figures

**Additional file 2** The metadata of the full list of 114 GWASs

**Additional file 3** Presumed causal genes included in the OMIM database

**Additional file 4** Genes suggested as causal by rare variant association studies

**Additional file 5** BioVU table

**Additional file 6** OMIM genes included in the analysis

**Additional file 7** Rare variant silver standard genes included in the analysis

**Additional file 8** PrediXcan and enloc results for predicted causal genes selected based on OMIM

**Additional file 9** PrediXcan and enloc results for presumed causal genes in the rare variant based silver standard

**Additional file 10** Review history

## Data Availability

Genotype-Tissue Expression (GTEx) project’s raw whole transcriptome and genome sequencing data are available via dbGaP accession number phs000424.v8.p2 [57]. All processed GTEx data are available via GTEx portal (http://gtexportal.org/). All the code used for the reproducible analysis is available, under MIT license, on Zenodo with the access code DOI 10.5281/zenodo.4321149[58] and GitHub https://github.com/hakyimlab/gtex-gwas-analysis[59]. The softwares for imputed summary results, *enloc*, *coloc*, PrediXcan, MultiXcan, *dap-g*, prediction models are available at links there in. 1000 Genomes Project Reference for LDSC, https://data.broadinstitute.org/alkesgroup/LDSCORE/1000G_Phase3_plinkfiles.tgz; 1000 Genomes Project Reference with regression weights for LDSC, https://data.broadinstitute.org/alkesgroup/LDSCORE/1000G_Phase3_weights_hm3_no_ MHC.tgz; BioVU, https://victr.vanderbilt.edu/pub/biovu/?sid=194; eCAVIAR, https://github.com/fhormoz/caviar; QTLEnrich, https://github.com/segrelabgenomics/eQTLEnrich; flashr, https://gaow.github.io/mnm-gtex-v8/analysis/mashr_flashr_workflow.html# flashr-prior-covariances; Gencode, https://www.gencodegenes.org/releases/26.html; GTEx GWAS subgroup repository, https://github.com/broadinstitute/gtex-v8; GTEx portal, http://gtexportal.org; Hail, https://github.com/hail-is/hail; HapMap Reference for LDSC, https://data.broadinstitute.org/alkesgroup/LDSCORE/w_hm3.snplist.bz2; LD score regression (LDSD regression), https://github.com/bulik/ldsc; MetaXcan, https://github.com/hakyimlab/MetaXcan; Mouse Phenotype Ontology, http://www.informatics.jax.org/vocab/mp_ontology; NHGRI-EBI GWAS catalog, https://www.ebi.ac.uk/gwas/; picard, http://picard.sourceforge.net/; PLINK 1.90, https://www.cog-genomics.org/plink2; PrediXcan, https://github.com/hakyim/PrediXcan; pyliftover, https://pypi.org/project/pyliftover/; Storeyś qvalue R package, https://github.com/StoreyLab/qvalue; Summary GWAS imputation, https://github.com/hakyimlab/summary-gwas-imputation; TORUS, https://github.com/xqwen/torus; UK Biobank GWAS, http://www.nealelab.is/uk-biobank/; UK Biobank, http://www.ukbiobank.ac.uk/
