# Supplementary material for: Exploiting the GTEx resources to decipher the mechanisms at GWAS loci
Source: Genome Biol. 2021 Jan 26;22:49. doi: 10.1186/s13059-020-02252-4 (PMC7836161; doi:10.1186/s13059-020-02252-4)
Supplement: Supplementary file 1 — Additional file 1 Supplementary Materials including detailed methods, tables, and figures [file 13059_2020_2252_MOESM1_ESM.pdf]

Supplementary Materials: Exploiting the GTEx resources to decipher the mechanisms at GWAS loci

Alvaro N Barbeira[1,†], Rodrigo Bonazzola[1,†], Eric R Gamazon[2,3,4,5,†], Yanyu Liang[1,†], YoSon Park[6,7,†], Sarah Kim-Hellmuth[8,9,10], Gao Wang[11], Zhuoxun Jiang[1], Dan Zhou[2], Farhad Hormozdiari[12, 13], Boxiang Liu[14], Abhiram Rao[14], Andrew R Hamel[12,15], Milton D Pividori[1], François Aguet[12], GTEx GWAS Working Group, Lisa Bastarache[16,17], Daniel M Jordan[18, 19, 20], Marie Verbanck[18, 19, 20, 21], Ron Do[18,19,20], GTEx Consortium, Matthew Stephens[11], Kristin Ardlie[12], Mark McCarthy[22], Stephen B Montgomery[23,24], Ayellet V Segrè[12, 15], Christopher D. Brown[6], Tuuli Lappalainen[9,10], Xiaoquan Wen[25], Hae Kyung Im[1,*]

1 Section of Genetic Medicine, Department of Medicine, The University of Chicago, Chicago, IL, USA
2 Division of Genetic Medicine, Department of Medicine, Vanderbilt University Medical Center, Nashville, TN, USA
3 Data Science Institute, Vanderbilt University, Nashville, TN, USA
4 Clare Hall, University of Cambridge, Cambridge, UK
5 MRC Epidemiology Unit, University of Cambridge, Cambridge, UK
6 Department of Genetics, University of Pennsylvania, Perelman School of Medicine, Philadelphia, PA, USA
7 Department of Systems Pharmacology and Translational Therapeutics, University of Pennsylvania, Perelman School of Medicine, Philadelphia, PA, USA
8 Statistical Genetics, Max Planck Institute of Psychiatry, Munich, Germany
9 New York Genome Center, New York, NY, USA
10 Department of Systems Biology, Columbia University, New York, NY, USA
11 Department of Human Genetics, University of Chicago, Chicago, IL, USA
12 The Broad Institute of MIT and Harvard, Cambridge, MA, USA
13 Department of Epidemiology, Harvard T.H. Chan School of Public Health, Boston, MA, USA
14 Department of Biology, Stanford University, Stanford, California 94305, USA
15 Ocular Genomics Institute, Massachusetts Eye and Ear, Harvard Medical School, Boston, MA, USA
16 Department of Biomedical Informatics, Department of Medicine, Vanderbilt University, Nashville, TN, USA
17 Center for Human Genetics Research, Department of Molecular Physiology and Biophysics, Vanderbilt University School of Medicine, Nashville, TN, USA
18 Department of Genetics and Genomic Sciences, Icahn School of Medicine at Mount Sinai, New York, New York, USA
19 Institute for Genomics and Multiscale Biology, Icahn School of Medicine at Mount Sinai, New York, New York, USA
20 The Charles Bronfman Institute for Personalized Medicine, Icahn School of Medicine at Mount sinai, New York, New York, USA
21 Université de Paris - EA 7537 BIOSTM, France
22 University of Oxford, United Kingdom
23 Department of Genetics, Stanford University, Stanford, CA, USA
24 Department of Pathology, Stanford University, Stanford, CA, USA
25 Department of Biostatistics, University of Michigan, Ann Arbor, MI, USA

†: These authors contributed equally to this work, alphabetic order;

∗: Correspondence to haky@uchicago.edu

List of Figures

S1 GWAS Summary Processing 10
S2 GWAS imputation quality 11
S3 GWAS Summary Imputation Deflation 11
S4 GWAS trait categories 12
S5 Number of models available in v8 fine-mapped-*mashr* family of models, compared to v7 Elastic Net family. 23
S6 Proportion of genes with a colocalized or associated signal using expression or splicing event. 24
S7 S-MultiXcan expression associations 31
S8 S-MultiXcan splicing associations 32
S9 Workflow of OMIM-based curation 33
S10 Selection of genes for testing silver standard 36
S11 ROC curves under permuted data 41
S12 Precision-recall curves of colocalization/association based methods on rare variant-based silver standard. 43
S13 Factor analysis using flashr to identify causal tissues. 46

List of Tables

S1 GWAS Metadata 9
S2 GWAS dataset list 13
S3 GWAS loci with colocalized or significant genes assigned. 28
S4 Presumed causal genes included in the OMIM database. 35
S5 Genes suggested as causal by rare variant association studies. 35
S6 Predictive value of different per-locus prioritization methods. 45

List of Additional Figures

A1 Schematic representation of LD contamination 18
A2 Diagram representation of mediation model. 19
A3 Causal gene prioritization using PrediXcan and *enloc*. 26
A4 Colocalization of expression QTLs for the 87 GWAS traits aggregated across the 49 tissues. 29
A5 Colocalization of splicing QTLs for each of the 87 GWAS traits aggregated across the 49 tissues. 30
A6 Data table for classification problem 36
A7 Distribution of the number of tested genes per GWAS locus overlapping OMIM- and rare variant-based silver standard. 38
A8 Precision-recall curves of colocalization/association based methods on OMIM silver standard. 40
A9 ROC curves under permuted data 42
A10 Precision-recall curves of enloc vs coloc 44

List of Additional Tables

A1 TAB:BIOVU 25
A2 Expression and splicing prediction models 27
A3 GWAS traits used for OMIM-based curation. 34

A4 OMIM genes included in the analysis 37
A5 Rare variant silver standard genes included in the analysis. 37
A6 Count of GWAS loci with predicted causal effects overlapping likely functional genes. 37
A7 PrediXcan and enloc results for predicted causal genes selected based on OMIM. 38
A8 PrediXcan and enloc results for presumed causal genes in the rare variant based silver standard. 39
A9 Enrichment and AUC for *coloc*, *enloc*, SMR, and PrediXcan 39

Contents

1 Terminology 6

2 Genotype-Tissue Expression (GTEx) Project 7
2.1 Whole-genome sequence data processing and quality control . . . 7
2.2 RNA-Seq data processing and quality control . . . 7

3 Genome-wide association studies (GWAS) data 8
3.1 Harmonization of GWAS summary statistics . . . 8
3.2 Imputation of GWAS summary statistics . . . 8
3.3 IGAP GWAS . . . 14
3.4 NHGRI-EBI GWAS catalog . . . 14

4 Correlated t-test to summarize across traits and tissues 14

5 Enrichment of QTLs among trait-associated variants 15

6 Cis-region and covariates used in fine-mapping and prediction of expression and splicing traits 15

7 Fine-mapping expression and splicing QTLs 15

8 Mediation analysis to quantify the dose-dependent effects of expression and splicing on traits 16
8.1 Selection of fine-mapped variants as instrumental variables and their effect sizes . . . 16
8.2 Correlation between GWAS and QTL effect sizes . . . 16
8.3 Modeling effect mediated by regulatory process . . . 16
8.4 Transcriptome-wide estimation of mediated effects . . . 17
8.5 Robustness of the estimation of the mediating effect to LD contamination . . . 17
8.6 Concordance of mediated effects for allelic series of independent eQTLs . . . 19

9 Identifying patterns of regulation of expression across tissues 20

10 Causal gene prioritization 20
10.1 Colocalization . . . 21
10.2 enloc . . . 21
10.3 coloc . . . 21

11 Fine-mapping of height GWAS using summary statistics 22

12 Association to predicted expression or splicing 22
12.1 Predicting the genetically regulated components of expression and splicing . . . 22
12.2 PrediXcan . . . 23
12.3 Colocalized and significantly associated genes . . . 23
12.4 S-MultiXcan . . . 24
12.5 PrediXcan replication in BioVU . . . 25
12.6 Summary-data-based Mendelian Randomization (SMR) and HEIDI . . . 28

13 Assessing the performance of association and colocalization methods to identify causal genes 32
13.1 OMIM-based curation of causal genes . . . 33
13.2 Rare variant association-based curation of causal genes . . . 35
13.3 Setting up the classification problem to quantify performance for identifying causal genes . . . 36

13.4 AUC of the ROC curves 39
13.5 Precision-recall curves of PrediXcan and *enloc* on the OMIM-based silver standard gene set 40
13.6 Precision-recall curves of PrediXcan and *enloc* on the rare variant association-based silver standard gene set 43
13.7 Comparing precision-recall curves of *coloc* and *enloc* 44
13.8 Assessing the contribution of proximity, colocalization, and association significance 44

14 Causal tissue analysis **45**

1 Terminology

For clarity and to reduce ambiguities, we provide the definition of some of the key terms used in the manuscript.

Trait: Here, trait (or complex trait) is used for observable, quantitative trait of individuals, such as presence of a disease or an anthropometric measurement. When speaking about traits, we do not include molecular phenotypes like gene expression or intron splicing quantification.

LD block/LD region: Region of the genome containing variants in LD among themselves, as determined from empirical LD patterns observed in 1000 Genomes [Berisa and Pickrell, 2016]. Variants in different LD blocks are unlikely to be correlated.

GWAS locus: This term is, in general, used somewhat loosely to refer to a region with a significantly associated variant which may span from tens to hundreds of kilobases depending on the LD of the region. However, here for quantification, we define it as one of the approximately independent LD blocks from [Berisa and Pickrell, 2016] that harbor a GWAS significant association. If multiple traits exist for a GWAS significant association in the block, we count them as distinct.

eQTL, eVariant: Here an eVariant is a genetic variant that is associated (FDR< 0.05) with the expression of a gene. eQTL refers to the variant-gene pair, in which the variant is an eVariant for the gene.

sQTL, sVariant: An sVariant is a genetic variant that is associated (FDR< 0.05) with the splicing (quantified as intron excision ratio) of a gene. sQTL refers to the variant-gene pair, in which the variant is an sVariant for the gene.

Fine-mapped variant: We call fine-mapped variant to the proxy for causal variant which we selected using *dap-g*'s posterior inclusion probabilities. These variants that are within credible sets with total posterior inclusion probability of at least 0.25 and have variant-level pip> 0.01. Within each credible set, one such variant is selected for our analysis.

LD contamination: This phenomenon occurs when the variant that alters the expression or splicing is distinct from the one that alters the complex trait, but they are in LD. In these circumstances, the QTL will be associated with the GWAS trait and the GWAS variant will be associated with the molecular trait, but there is no causal relationship between the gene and the complex trait.

Posterior inclusion probability (pip): This is the probability that a variant has a causal effect on a trait. These probabilities are calculated by Bayesian fine-mapping approaches such as *dapg* and *susier*.

PrediXcan: This term refers to the family of methods that seeks to identify causal genes by correlating the genetic component of gene expression (mRNA level and splicing) with the trait. This family includes S-PrediXcan (which uses GWAS summary statistics rather than individual level data) and MultiXcan (which aggregates evidence of associations across all tissues leveraging the fact that e/sQTLs are shared across tissues). We use PrediXcan as a generic term to refer to this family of methods.

Silver standard genes: To test the ability of colocalization and association methods to identify true causal genes, we curated a set of 'causal' genes. To emphasize the imperfect nature, we use the term silver standard genes. In this context, the term OMIM gene is used as the causal gene for the trait.

2 Genotype-Tissue Expression (GTEx) Project

All processed Genotype-Tissue Expression (GTEx) Project v8 data have been made available on dbGAP (accession ID: phs000424.v8). Primary and extended results generated by consortium members are available on the Google Cloud Platform storage accessible via the GTEx Portal (see URLs).The GTEx Project v8 data, based on 17,382 RNA-sequencing samples from 54 tissues of 948 post-mortem subjects, has established the most comprehensive map of regulatory variation to date. In addition to the larger sample size and greater tissue coverage compared to v6, v8 data also included whole-genome sequencing data, facilitating high resolution QTL map of 838 subjects for 49 tissues with at least 70 samples. The GTEx consortium mapped complex trait associations for 23,268 cis-eGenes and 14,424 cis-sGenes [The GTEx Consortium, 2020]. We did not include trans QTLs in our analyses due to limited power after correcting for confounders and potential pleiotropic effect in complex trait associations. Below, we briefly describe the whole-genome sequencing, RNA-sequencing and QTL data processing protocols. Detailed description of subject ascertainment, sample procurement, and sequencing data processing are available elsewhere [The GTEx Consortium, 2020].

2.1 Whole-genome sequence data processing and quality control

Out of 899 WGS samples sequenced at an average coverage of 30x on HiSeq200 (68 samples) and HiSeqX (all other samples), variant call files (VCF) for 866 GTEx donors were included in downstream analyses after excluding one each from 30 duplicate samples and three donors. Of these, 838 subjects with RNA-seq data were included for QTL mapping and subsequent complex trait association analyses in our study. All whole-genome sequencing data were mapped to GRCh38/hg38 reference.

2.2 RNA-Seq data processing and quality control

Whole transcriptome RNA-Seq data were aligned using STAR (v2.5.3.a; [Dobin et al., 2013]). For STAR index, GENCODE v26 (GRCh38; see URLs) was used with the sjdbOverhang 75 for 76-bp paired-end sequencing protocol. Default parameters were used for RSEM (see URLs; [Li and Dewey, 2011]) index generation. GTEx utilized Picard (see URLs) to mark and remove potential PCR duplicates and RNA-SeQC [DeLuca et al., 2012] to process post-alignment quality control. RSEM was then used for per-sample transcript quantification. Subsequently, read counts were normalized between samples using TMM [Robinson and Oshlack, 2010]. For eQTL analyses, latent factor covariates were calculated using PEER as follows: 15 factors for N<150 per tissue; 30 factors for 150<=N<250; 45 factors for 250<=N<350; and 60 factors for N>=350. Finally, fastQTL [Ongen et al., 2016] was used for cis-eQTL mapping in each tissue. Only protein-coding, lincRNA, and antisense biotypes as defined by Gencode v26 were considered for further analyses. To study alternative splicing, GTEx applied LeafCutter (version 0.2.8; [Li et al., 2018]) using default parameters to quantify splicing QTLs in cis with intron excision ratios [The GTEx Consortium, 2020].

3 Genome-wide association studies (GWAS) data

3.1 Harmonization of GWAS summary statistics

The process followed for the harmonization and imputation are depicted in Fig. S1. For each standardized GWAS summary statistics, we mapped all variants to hg38 (GRCh38) references using *pyliftover* (see URLs). For missing chromosome or genomic position information in the original GWAS summary statistics file, we queried dbSNP build 125 (hg17), dbSNP build 130 (hg18/GRCh36), and dbSNP build 150 (hg19/GRCh37) using the provided variant rsID information and the original reference build of the GWAS summary statistics file. Variants with missing chromosome, genomic position, and rsID information were excluded from further analyses. Only autosomal variants were included in our analyses. Missing allele frequency information was filled using the allele frequencies estimated in the GTEx (v8) individuals of genotype-based European genetic ancestry (here onwards, GTEx-EUR) whenever possible. We excluded variants with discordant reference and alternate allele information between GTEx and the GWAS study. We included only the alleles with the highest MAF among multiple alternate alleles if the variant was reported as multiallelic in GTEx. When more than one GWAS variant mapped to a given GTEx variant (i.e., the same chromosomal location in hg38), only the one with the highest significance was retained. For binary traits, if the sample size was present but the number of cases was missing, we filled the missing count with the sample size and number of cases reported in the paper. For continuous traits, if the file contained the sample size for each variant, the reported number was used. If not, we filled this value using the number reported in the corresponding publication. If only some variants were missing sample size information, we filled the missing value with the median of all reported values.

3.2 Imputation of GWAS summary statistics

To standardize the number of variants across tissue-trait pairs, all processed GWAS results were imputed. We implemented the Best Linear Unbiased Prediction (BLUP) approach [Lee et al., 2013; Pasaniuc et al., 2014] in-house (`https://github.com/hakyimlab/summary-gwas-imputation`) to impute z-scores for those variants reported in GTEx without matching data in the GWAS summary statistics. This algorithm does not impute raw effect sizes (β coefficients). The imputation was performed in specific regions assumed to have sufficiently low correlations between them, defined by approximately independent linkage disequilibrium (LD) blocks [Berisa and Pickrell, 2016] lifted over to hg38/GRCh38.

Only GTEx variants with MAF > 0.01 in GTEx-EUR subjects were used in downstream analyses. Covariance matrices (reference LD information) were estimated on these GTEx-EUR subjects. The corresponding (pseudo-)inverse matrices for covariances C were calculated via Singular Value Decomposition (SVD) using ridge-like regularization $C + 0.1I$. To avoid ambiguous strand issues homogeneously, palindromic variants (i.e. CG) were excluded from the imputation input. Thus, an imputed z-score was generated for palindromic variants available in the original GWAS; for them, we report the absolute value of the original entry with the sign from the imputed z-score. The sample size that we report for the imputed variants is the same as the sample size for the observed ones if it is reported as constant across variants, or their median if it changes across the observed variants, which occurs in the case of meta-analyses.

We initially considered publicly available GWAS summary statistics for 114 complex traits provided by large-scale consortia and the UK Biobank [Bycroft et al., 2018] (Additional Table S1). Of these, 27 studies with a relatively small intersection of variants with the GTEx panel (number of variants$< 2 \times 10^6$, compared to almost 9×10^6 variants available in GTEx) exhibited significant deflation of their association p-values (Fig. S3). Thus, all analyses focused on 87 traits where missing variants could be properly imputed unless otherwise stated explicitly (Table S2). We observed noteworthy association prediction performance across the selected 87 traits (e.g., with a median $r^2 = 0.90$ (IQR = 0.0268) between the original and imputed zscores on chromosome 1). The median slope was 0.94 (IQR = 0.0164), as the imputed zscore values tend to be more conservative than the original ones. Imputation quality was consistent across traits, depending

Table S1: The metadata of the full list of 114 GWASs. (See Additional file 2) contains relevant information concerning each GWAS study used. Full table available in Supplementary Material. Analyses used the 87 traits with deflation=0 unless explictly said otherwise. Columns are: **Tag**: Internal name to identify the study, **Deflation**: Deflation status after imputation (0 for no deflation, 1 for moderate deflation, 2 for extreme deflation), **PUBMED_Paper_Link**: PUBMED entry, **Pheno_File**: name of downloaded file, **Source_File**: actual name of GWAS summary statistics (i.e. downloaded files might contain several traits), **Portal**: URL to GWAS study portal, **Consortium**: Name of Consortium if any, **Link**: download link for the file, **Notes**: any special comment on the GWAS trait, **Header**: GWAS summary statistics header in case the file is malformed, **EFO**: Experimental Factor Ontology [Malone et al., 2010] entry if applicable, **HPO**: Human Phenotype Ontology [Köhler et al., 2013] entry if applicable, **Description**: optional description of the study, **Trait**: trait name, **Sample_Size**: number of individuals included in the study, **Population**: types of populations present (EUR for European, AFR for African, EAS for East Asian, etc), **Date**: Date the file was downloaded, **Declared_Effect_Allele**: column specifying effect allele, **Genome_Reference**: Human Genome release used as reference (i.e. hg19, hg38), **Binary**: wether the trait is dichotomous, **Cases**: number of cases if binary trait, **abbreviation**: short string for figure and table display, **new_abbreviation**: additional abbreviation, **new_Trait**: additional trait name, **Category**: type of trait, **Color**: Hexadecimal color code for display

strongly on the number of input available variants (Fig. S2). The main reason to drop the 27 traits were to keep the consortium's multiple papers consistent. None of the conclusions in this paper changed when including all 114 traits.

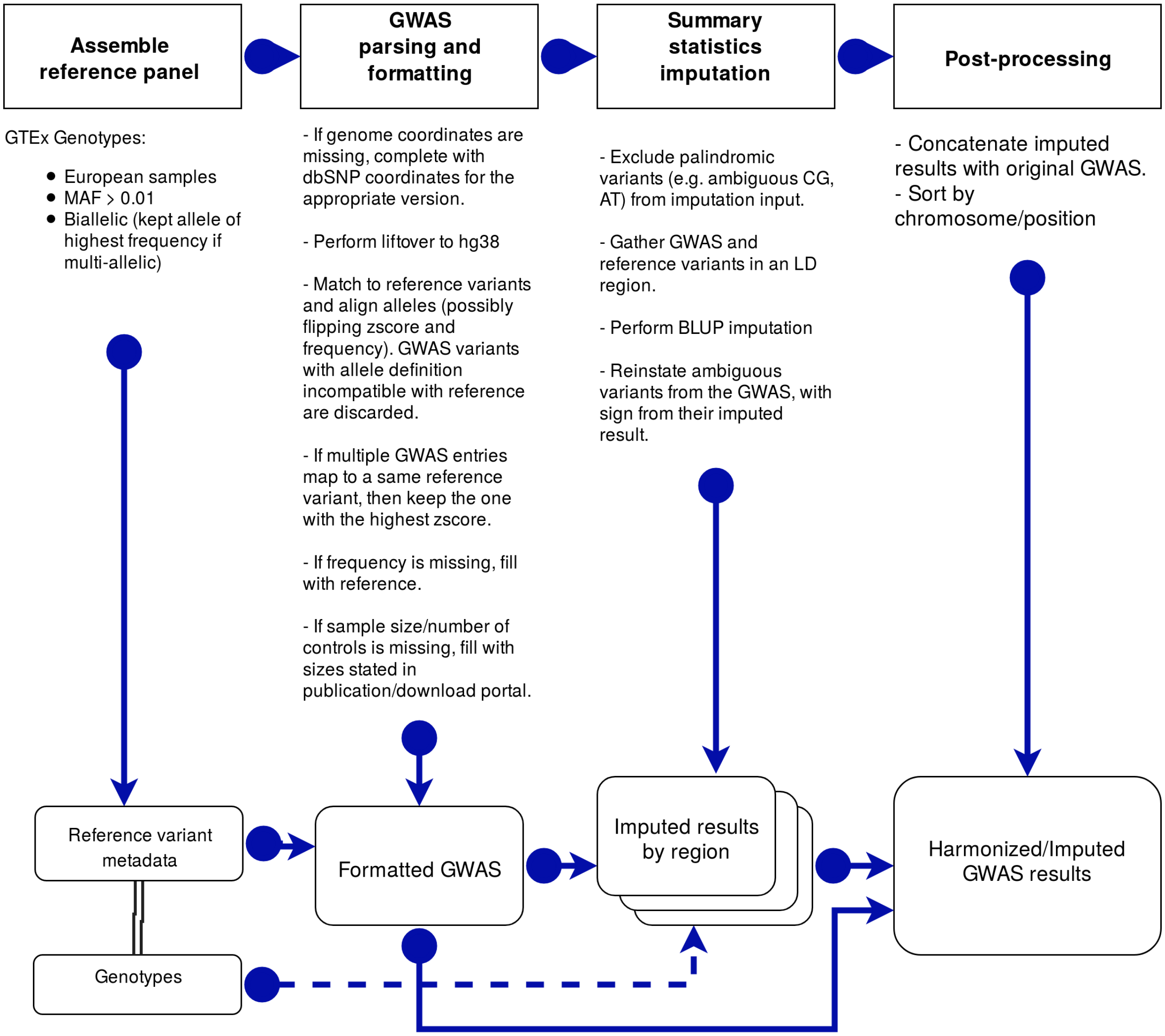

Fig S1. Workflow of GWAS results processing.

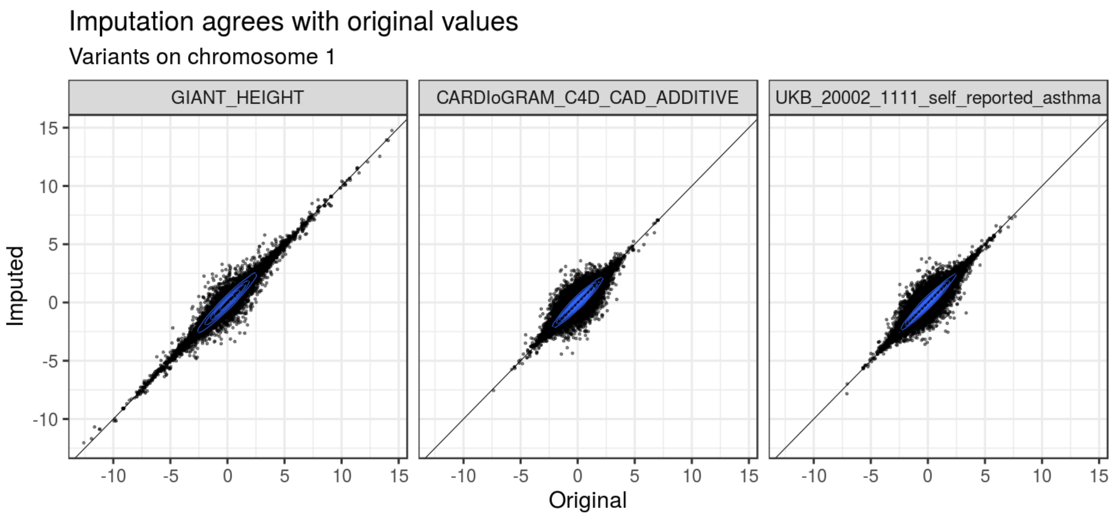

Fig S2. GWAS imputation quality Original versus imputed zscores for palindromic variants in chromosome 1 for 3 traits.

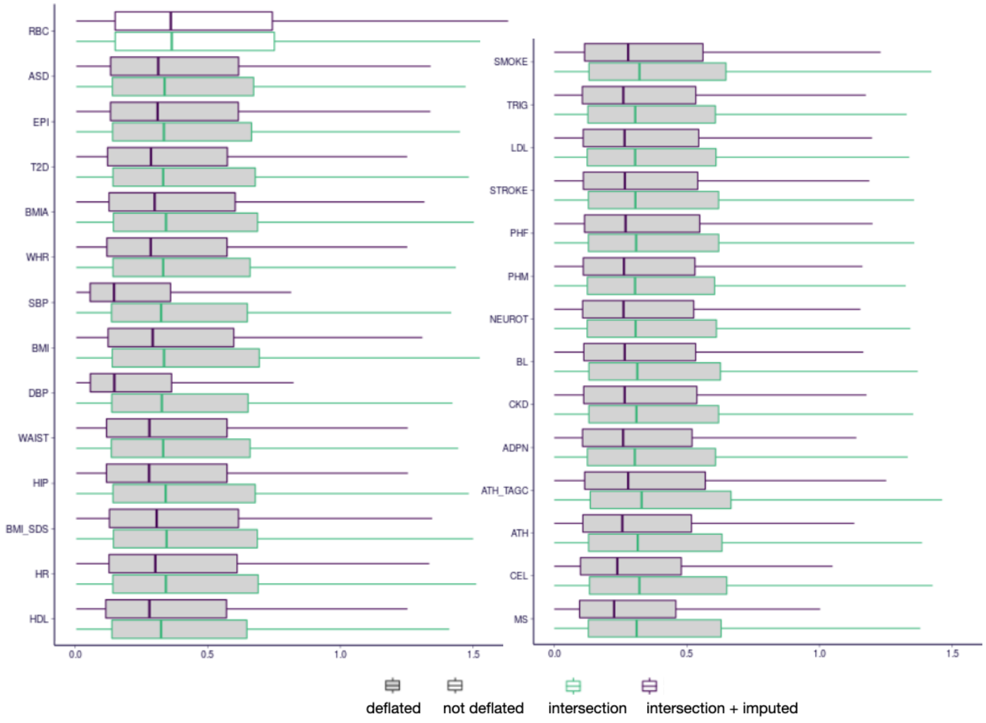

Fig S3. GWAS imputation deflation This figure compares the distribution of p-values for 28 GWAS traits before and after imputation. Vertical scale shows -log10(p-value) of variant association. The 27 traits that exhibited deflation are filled in gray. An undeflated trait (e.g., Red Blood Cell count) is included for comparison. See trait abbreviation list in Additional Table S1.

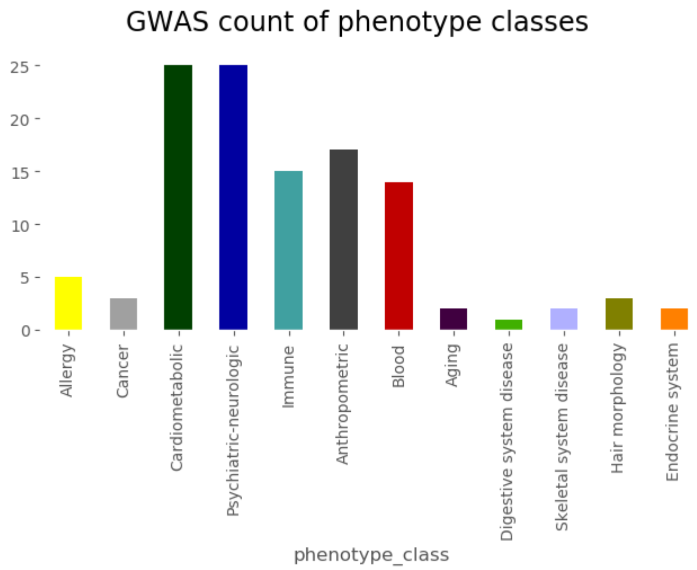

Fig S4. GWAS trait categories. Categories of the traits with full GWAS summary statistics used in the analysis. See list of traits in Table S2.

Table S2: List of 87 GWAS datasets

Category	Trait	Abbreviation	Sample_Size
Psychiatric-neurologic	Alzheimers Disease	AD	54162
Psychiatric-neurologic	Attention Deficit Hyperactivity Disorder	ADHD	53293
Psychiatric-neurologic	Chronotype	CHRONO	128266
Psychiatric-neurologic	Chronotype UKB	CHRONO_UKB	337119
Psychiatric-neurologic	Depressive Symptoms	DEPR	180866
Psychiatric-neurologic	Education Years	EDU	293723
Psychiatric-neurologic	Epilepsy UKB	EPI_UKB	337119
Psychiatric-neurologic	Fluid Intelligence Score UKB	FIS_UKB	337119
Psychiatric-neurologic	Insomnia In Both Sexes	INSOMN	113006
Psychiatric-neurologic	Insomnia UKB	INSOMN_UKB	337119
Psychiatric-neurologic	Insomnia UKBS	INSOMN_UKBS	337119
Psychiatric-neurologic	Migraine UKB	MIGR_UKB	337119
Psychiatric-neurologic	Migraine UKBS	MIGR_UKBS	337119
Psychiatric-neurologic	Multiple Sclerosis UKBS	MS_UKBS	337119
Psychiatric-neurologic	Neuroticism UKB	NEUROT_UKB	337119
Psychiatric-neurologic	Parkinsons Disease UKBS	PD_UKBS	337119
Psychiatric-neurologic	Psychological Problem UKBS	PSY_UKBS	337119
Psychiatric-neurologic	Schizophrenia	SCZ	150064
Psychiatric-neurologic	Schizophrenia UKBS	SCZ_UKBS	337119
Psychiatric-neurologic	Sleep Duration	SLEEP	128266
Psychiatric-neurologic	Sleep Duration UKB	SLEEP_UKB	337119
Anthropometric	BMI UKB	BMI_UKB	337119
Anthropometric	Birth Weight	BW	143677
Anthropometric	Birth Weight UKB	BW_UKB	337119
Anthropometric	Body Fat Percentage UKB	FAT_UKB	337119
Anthropometric	Bone Mineral Density	BMD	49988
Anthropometric	Height	HEIGHT	253288
Anthropometric	Intracraneal Volume	ICV	30717
Anthropometric	Standing Height UKB	HEIGHT_UKB	337119
Cardiometabolic	CH2DB NMR	CH2	24154
Cardiometabolic	Coronary Artery Disease	CAD	184305
Cardiometabolic	Deep Venous Thrombosis UKB	DVT_UKB	337119
Cardiometabolic	Deep Venous Thrombosis UKBS	DVT_UKBS	337119
Cardiometabolic	Fasting Glucose	FG	46186
Cardiometabolic	Fasting Insulin	INSUL	38238
Cardiometabolic	HDL Cholesterol NMR	HDLC	19270
Cardiometabolic	Heart Attack UKB	MI_UKB	337119
Cardiometabolic	High Cholesterol UKBS	HC_UKBS	337119
Cardiometabolic	Hypertension UKBS	HPT_UKBS	337119
Cardiometabolic	LDL Cholesterol NMR	LDLC	13527
Cardiometabolic	Pulmonary Embolism UKB	PE_UKB	337119
Cardiometabolic	Triglycerides NMR	IDL	21559
Cardiometabolic	Type 2 Diabetes UKBS	T2D_UKBS	337119
Blood	Eosinophil Count	EC	173480
Blood	Granulocyte Count	GC	173480
Blood	High Light Scatter Reticulocyte Count	HRET	173480
Blood	Lymphocyte Count	LC	173480
Blood	Monocyte Count	MC	173480
Blood	Myeloid White Cell Count	MWBC	173480
Blood	Neutrophil Count	NC	173480
Blood	Platelet Count	PLT	173480
Blood	Red Blood Cell Count	RBC	173480
Blood	Reticulocyte Count	RET	173480
Blood	Sum Basophil Neutrophil Count	BNC	173480
Blood	Sum Eosinophil Basophil Count	EBC	173480
Blood	Sum Neutrophil Eosinophil Count	NEC	173480
Blood	White Blood Cell Count	WBC	173480
Cancer	Breast Cancer	BC	120000
Cancer	ER-negative Breast Cancer	ERNBC	120000
Cancer	ER-positive Breast Cancer	ERPBC	120000
Allergy	Asthma UKBS	ATH_UKBS	337119
Allergy	Eczema	ECZ	116863
Allergy	Eczema UKBS	ECZ_UKBS	337119
Immune	Ankylosing Spondylitis UKBS	ASP_UKBS	337119
Immune	Asthma UKB	ATH_UKB	337119
Immune	Crohns Disease	CD	20833
Immune	Crohns Disease UKBS	CD_UKBS	337119
Immune	Hayfever UKB	HAY_UKB	337119
Immune	Inflammatory Bowel Disease	IBD	34652
Immune	Inflammatory Bowel Disease UKBS	IBD_UKBS	337119
Immune	Psoriasis UKBS	PSO_UKBS	337119
Immune	Rheumatoid Arthritis	RA	80799
Immune	Rheumatoid Arthritis UKBS	RA_UKBS	337119
Immune	Systemic Lupus Erythematosus	SLE	23210
Immune	Type 1 Diabetes UKBS	T1D_UKBS	337119
Immune	Ulcerative Colitis	UC	27432
Immune	Ulcerative Colitis UKBS	UC_UKBS	337119
Aging	Fathers Age At Death UKB	FAD_UKB	337119
Aging	Mothers Age At Death UKB	MAD_UKB	337119
Digestive system disease	Irritable Bowel Syndrome UKBS	IBS_UKBS	337119
Endocrine system disease	Hyperthyroidism UKBS	HYPERTHY_UKBS	337119
Endocrine system disease	Hypothyroidism UKBS	HYPOTHY_UKBS	337119
Skeletal system disease	Gout UKBS	GOUT_UKBS	337119
Skeletal system disease	Osteoporosis UKBS	OST_UKBS	337119
Morphology	Balding Pattern 2 UKB	BLDP2_UKB	337119
Morphology	Balding Pattern 3 UKB	BLDP3_UKB	337119
Morphology	Balding Pattern 4 UKB	BLDP4_UKB	337119

3.3 IGAP GWAS

We used summary results from an Alzheimer's Disease study from International Genomics of Alzheimer's Project (IGAP).

IGAP is a large two-stage study based upon genome-wide association studies (GWAS) on individuals of European ancestry. In stage 1, IGAP used genotyped and imputed data for 7,055,881 single nucleotide polymorphisms (SNPs) to meta-analyze four previously-published GWAS datasets consisting of 17,008 Alzheimer's disease cases and 37,154 controls (The European Alzheimer's disease Initiative - EADI the Alzheimer Disease Genetics Consortium - ADGC The Cohorts for Heart and Aging Research in Genomic Epidemiology consortium - CHARGE The Genetic and Environmental Risk in AD consortium - GERAD). In stage 2, 11,632 SNPs were genotyped and tested for association in an independent set of 8,572 Alzheimer's disease cases and 11,312 controls. Finally, a meta-analysis was performed combining results from stages 1 & 2.

3.4 NHGRI-EBI GWAS catalog

In addition to the GWAS summary statistics described above, we obtained the list of trait-associated SNPs from the GWAS catalog [Buniello et al., 2019] (downloaded on 9/7/2018), which, at download, contained 80,727 entries. To measure the enrichment of e/sQTL in the GWAS Catalog, we computed the proportion of e/sQTL in the GWAS catalog relative to the proportion of e/sQTL among all GTEx V8 variants. We then obtained a measure of the uncertainty in the proportion and enrichment-fold using block jackknife. See [The GTEx Consortium, 2020] for details.

4 Correlated t-test to summarize across traits and tissues

Most statistics shown in these analyses are at the tissue-trait level. There are 4,263 statistics, generated from 49 tissues and 87 traits. Typical statistical tests assume the data from which the statistic is computed is sampled independently and identically distributed (IID). Among different tissues, there are wide ranges of standard errors and different patterns of correlation. Because of this, the IID assumption can not be applied to the tissue-trait statistics. Therefore, we describe our derivation of standard errors when statistics are summarized across traits for a given tissue, and when statistics are summarized across tissue and trait pairs. In the following paragraphs, we use S_{tp} to indicate a statistic estimated in tissue t and trait p. This statistic has standard error se(S_{tp}).

Summarizing across traits for a given tissue. When we have one statistic per tissue-trait pair and summarize across traits in a given tissue, we assume the traits are independent, but we take into account the differences in standard errors. For each tissue t, we summarized $S_{t1}, \cdots, S_{tP}$ by fitting the following linear model:

$$S_{tp} = \mu_S^t + \epsilon_{tp} \tag{1}$$

$$\epsilon_{tp} \sim N(0, \text{se}(S_{tp})^2 \times \sigma_t^2) \tag{2}$$

So $\hat{\mu}_S^t$ is an estimate for the statistic S summarized across all traits in tissue t, and this estimate has standard error se$(\hat{\mu}_S^t)$. This is essentially a weighted average across traits.

Summarizing across trait and tissue pairs. When we summarize across all tissue-trait pairs, $S_{11}, \cdots,\ S_{tp}, \cdots, S_{TP}$, we fit a similar linear model, which allows for correlation between tissues and correlation between traits, and corrects for differences in the standard errors.

$$S_{tp} = \mu_S + \mu_S^t + \mu_S^p + \epsilon_{tp} \quad (3)$$
$$\mu_S^t \sim N(0, \sigma_T^2) \quad (4)$$
$$\mu_S^p \sim N(0, \sigma_P^2) \quad (5)$$
$$\epsilon_{tp} \sim N(0, \text{se}(S_{tp})^2 \times \sigma^2), \quad (6)$$

Here, μ_S^t is the tissue-specific random intercept, and μ_S^p is the trait-specific random intercept. These components account for features common across traits that are specific to tissue t and features common across tissues that are specific to trait p respectively. The estimate $\hat{\mu}_S$ is the weighted average of S_{tp} across all tissue-trait pairs, and its standard error is se$(\hat{\mu}_S)$.

Testing whether two statistics have different mean. We would often like to test whether two statistics are different, *e.g.* enrichment signal measured for sQTL as μ_{S_1} versus enrichment signal measured for eQTL as μ_{S_2}. For this, we need to construct a test aggregating pairwise differences across all tissue-trait pairs. For this purpose, we constructed the following paired test. Our test statistic is $T^{tp} := S_{1,tp} - S_{2,tp}$ with se$(T^{tp}) = \sqrt{\text{se}(S_{1,tp})^2 + \text{se}(S_{2,tp})^2}$. We calculate $\hat{\mu}_T$ by summarizing across all tissue-trait pairs as described in the previous paragraph. Under the null $\mathcal{H}_0 : \mu_{S_1} = \mu_{S_2}$ and $\hat{\mu}_T \sim N(0, \text{se}(\hat{\mu}_T))$.

5 Enrichment of QTLs among trait-associated variants

To estimate the proportion of SNPs considered as associated with expression (for at least one gene) at various p-value thresholds, we used the most significant p-value (tested using all GTEx individuals) for each SNP from all associations in all tissues (including all genes and variants tested). We observed that the proportion of variants associated with expression and splicing at different significance threshold was much larger for trait-associated variants from the GWAS catalog than for the full set of tested common variants (Fig. 2). At a nominal threshold, the proportion of common variants associated with the expression of a gene in some tissue increased from 92.7% in the V6 release [GTEx Consortium et al., 2017] to 97.3% in V8. For splicing, the proportion was 97.7%. These results should serve as a cautionary note that assigning function to a GWAS locus based on QTL association p-value alone, even with a more stringent threshold, could be misleading.

6 Cis-region and covariates used in fine-mapping and prediction of expression and splicing traits

For each gene, we considered all variants within the cis-window (1Mbps) with MAF>0.01, and used the same covariates as in the GTEx v8 main eQTL analysis: sex, WGS plaform, WGS library preparation protocol, top 5 genetic principal components, and PEER factors. The number of PEER factors was determined from the sample size: 15 for $n < 150$, 30 for $150 \leq n < 250$, 45 for $250 \leq n < 350$, 60 for $350 \leq n$.

7 Fine-mapping expression and splicing QTLs

We applied *dap-g* [Wen, 2016] to the 49 tissues to estimate the degree to which a variant might exert a causal effect on expression or splicing levels, using default parameter values. First, we selected genes annotated as protein-coding, lincRNA or pseudogenes. We used the covariates listed in the supplement

section 6. This yielded a list of clusters (variants related by LD), and posterior inclusion probabilities (*pip*) that provide an estimate of the probability of a variant being causal. We repeated this process for splicing ratios from Leafcutter, using a cis-window ranging from 1Mbps upstream of the splicing event start location to 1Mbps downstream of the end location. We used individual-level data for GTEx-EUR subjects both for expression and splicing. We note that the main report of the GTEx v8 included individuals of non-European descent and reported only expression QTL fine-mapping. Sample sizes ranged from 65 in kidney cortex to 602 in skeletal muscle tissues. All results are made publicly available (https://github.com/hakyimlab/gtex-gwas-analysis).

8 Mediation analysis to quantify the dose-dependent effects of expression and splicing on traits

Enrichment of expression and splicing QTLs suggest a causal role of molecular trait regulation on complex traits. However, confounders such as LD contamination could be inflating these results limiting their interpretation. Here, we sought to gather stronger evidence for a causal link. We tested whether there is a dose-dependent effect of expression and splicing QTLs on complex traits and also whether independent QTLs provided similar measures of the mediated effects.

8.1 Selection of fine-mapped variants as instrumental variables and their effect sizes

To investigate the relationship between GWAS and QTL effect sizes in the transcriptome, we generated a set of fine-mapped QTL signals derived from *dap-g* fine-mapping performed in the GTEx-EUR individuals to serve as proxy for causal QTLs. For splicing, we utilized sQTLs at the splicing event/variant level rather than the gene/variant level. We considered only variants within credible sets with at least 25% total probability. Within each credible set, the variant with highest posterior inclusion probability was selected as the fine-mapped variant. Only variants with variant-level *pip* of at least 0.01 were considered.

For each of the selected QTLs, we used the QTL effect size estimated from the marginal test (using the GTEx-EUR individuals) and the GWAS effect size reported by the study or if missing, calculated from the imputed z-score from the GWAS imputation by $\hat{\beta} \approx z/\sqrt{f(1-f)N}$, where f is the allele frequency and N is the GWAS sample size.

8.2 Correlation between GWAS and QTL effect sizes

To get a first-order approximation to the mediated effect sizes without imposing any modeling assumptions, we calculated the Pearson correlation of the magnitude of observed GWAS effect size and of cis-eQTL effect size, $\widehat{\text{Cor}}(|\hat{\delta}_k|, |\hat{\gamma}_k|)$, for the list of selected fine-mapped QTLs. This was done for each tissue-trait pair separately. The observed Pearson correlation captures the mediated effect (see details in Section 8.5). To obtain a null distribution for the correlation that accounts for the potential confounding effect of different local LD score values, we computed the Pearson correlation under the shuffled data within each LD-score bin defined by quantiles (100 bins were used). The significance of the difference between observed and null distribution was calculated using the correlated t-test method described in Section 4.

8.3 Modeling effect mediated by regulatory process

We compared the magnitude of GWAS and cis-QTL effect sizes, which is the basis of multi-SNP Mendelian randomization approaches [Bowden et al., 2015].

To formalize the relationship between the GWAS effect size (δ) and the QTL effect size (γ), we assumed an additive genetic model for the GWAS trait. Specifically, for variant k,

$$Y = \sum_k \delta_k \cdot X_k + \epsilon, \tag{7}$$

where X_k is the allele count of variant k, Y is the trait, and ϵ is the un-explained variation. We decomposed GWAS effect size into its mediated and un-mediated components,

$$\delta_k = \sum_{g \in \mathcal{G}_k} \beta_g \gamma_{k,g} + \nu_k, \tag{8}$$

where $\mathcal{G}_k$ represents the set of genes regulated by variant k with corresponding QTL effect size as $\gamma_{k,g}$, and ν_k is the un-mediated effect of variant k on trait. And β_g is the downstream effect of gene g on the trait.

8.4 Transcriptome-wide estimation of mediated effects

To estimate the transcriptome-wide contribution of the mediated effects on complex traits, we proposed a mixed-effects model on the basis of Eq. 8,

$$|\delta_k| = \beta_g \cdot (\text{sign}(\delta_k) \cdot \gamma_{k,g}) + b_0 + b_1 \cdot \sqrt{\text{LD-score}_k} + \epsilon \tag{9}$$

$$\beta_g \sim N(0, \sigma^2_{\text{gene}}) \tag{10}$$

$$\epsilon \sim N(0, \sigma^2), \tag{11}$$

where b_0, b_1 are the fixed effect capturing the un-mediated effect and β_g is the mediated effect of the gene or splicing event g. In short, we assumed a random effects model to account for the heterogeneity of the β's and aimed at estimating σ^2_{gene} as the transcriptome-wide average of the mediated effect. For each tissue-trait pair, we fitted the model using selected fine-mapped QTLs, as described in Section 8.1, along with the corresponding $\hat{\delta}_k$ (GWAS effect for variant k), $\hat{\gamma}_{k,g}$ (QTL effect for variant k, gene g). To obtain the distribution of σ^2_{gene} under the null, we performed the same calculation using shuffled GWAS effect sizes. The effect allele choice is arbitrary and we chose them so that all the GWAS effects are positive. This choice made the modeling of the effect of local LD more straightforward since we expect that variants in high LD regions may tag more causal variants and end up with a larger estimated GWAS effect, which would result in a positive b_1. The square root of LD-score represents better the potential effect of LD score on the effect size. Using absolute value of the GWAS effects ($|\delta_k|$) and sign$(\delta_k) \cdot \gamma_{k,g}$ in equation (9) is a convenient way to implement the recoding of the effect allele.

8.5 Robustness of the estimation of the mediating effect to LD contamination

We illustrate the intuition behind the LD-contamination correction when the average mediated effects are estimated using the approximate method (correlation of absolute values) or the mixed-effects approach.

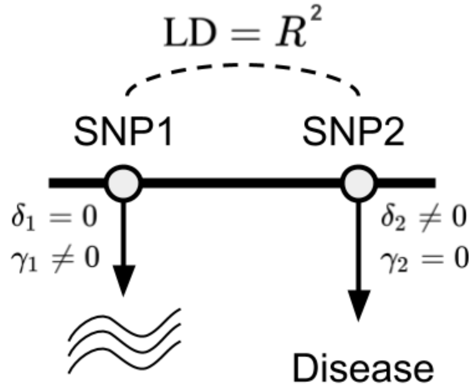

Additional Fig. A1: Schematic representation of LD contamination. SNP1 has a causal effect on the expression level of a gene but not on the trait (Disease here), $\delta_1 = 0$ and $\gamma_1 \neq 0$. SNP2 has a causal effect on the trait but not on the expression of the gene, $\delta_2 \neq 0$ and $\gamma_2 = 0$.

Consider the LD-contamination scenario where SNP 1 and SNP 2 are in LD with correlation R^2 (suppose LD is fixed) and have a non-zero effect on gene expression and trait, respectively (as shown in Fig. A1). The marginal effect estimates of SNP 1, *i.e.* $\hat{\delta}_1$ and $\hat{\gamma}_1$, are given by

$$\hat{\delta}_1 = R\delta_2 + \epsilon_{\text{GWAS}} \tag{12}$$
$$\hat{\gamma}_1 = \gamma_1 + \epsilon_{\text{QTL}}, \tag{13}$$

where Eq. 12 holds because the marginal effect size depends on LD. To determine the covariance of the magnitude of the GWAS and QTL estimates for SNP 1, we consider $\mathsf{E}(|\hat{\delta}_1||\hat{\gamma}_1|)$.

$$\begin{aligned} \mathsf{E}(\hat{\delta}_1\hat{\gamma}_1 \,|R) &= \mathsf{E}((R\delta_2 + \epsilon_{\text{GWAS}}) \cdot (\gamma_1 + \epsilon_{\text{QTL}}) \,|R) && (14)\\ &= \mathsf{E}(R\delta_2\gamma_1 \,|R) + \mathsf{E}(\epsilon_{\text{GWAS}}\gamma_1) + \mathsf{E}(R\delta_2\epsilon_{\text{QTL}} \,|R) + \mathsf{E}(\epsilon_{\text{GWAS}}\epsilon_{\text{QTL}}) && (15)\\ &= R \cdot \mathsf{E}(\delta_2\gamma_1), && (16) \end{aligned}$$

where Eq. 16 holds since the last three terms in the previous line are zeros, due to the independence among ϵ_{GWAS}, ϵ_{QTL}, and true effect sizes, δ and γ.

Hence, the covariance of the GWAS and QTL effect sizes under the LD contamination scenario is

$$\begin{aligned} \mathsf{Cov}(\hat{\delta}_1, \hat{\gamma}_1 \,|R) &= \mathsf{E}(\hat{\delta}_1\hat{\gamma}_1 \,|R) - \mathsf{E}(\hat{\delta}_1 \,|R) \cdot \mathsf{E}(\hat{\gamma}_1 \,|R) && (17)\\ &= R \cdot \mathsf{E}(\delta_2\gamma_1) - \mathsf{E}(\hat{\delta}_1 \,|R) \cdot \mathsf{E}(\hat{\gamma}_1 \,|R) && (18)\\ &= R \cdot \mathsf{E}(\delta_2\gamma_1) - \mathsf{E}(R\delta_2 + \epsilon_{\text{GWAS}}) \cdot \mathsf{E}(\gamma_1 + \epsilon_{\text{QTL}}) && (19)\\ &= R \cdot \mathsf{E}(\delta_2\gamma_1) - R \cdot \mathsf{E}(\delta_2) \cdot \mathsf{E}(\gamma_1) && (20)\\ &= R \cdot \mathsf{Cov}(\delta_2, \gamma_1), && (21) \end{aligned}$$

which implies that conditioning on LD, the observed correlation between $\hat{\delta}$ and $\hat{\gamma}$ should be very small.

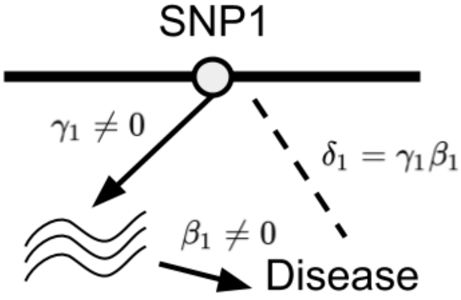

Additional Fig. A2: Diagram representation of mediation model.

Similarly, we can derive the correlation between GWAS and QTL effect size estimates under the simple mediation model shown in Fig. A2, where we have

$$\hat{\delta}_1 = \beta_1\gamma_1 + \epsilon_{\text{GWAS}} \tag{22}$$

$$\hat{\gamma}_1 = \gamma_1 + \epsilon_{\text{QTL}}, \tag{23}$$

where Eq. 22 follows by definition of the mediation model considering no direct effect. So,

$$\text{Cov}(\hat{\delta}_1, \hat{\gamma}_1 \,|\beta_1) = \beta_1\text{E}(\gamma_1^2) - \beta_1\text{E}(\gamma_1)^2 \tag{24}$$

$$= \beta_1\text{Var}(\gamma_1) \tag{25}$$

So, if we consider a gene locus, which naturally conditions on local LD and gene-level effect β, we can conclude that

$$\text{Cov}(\hat{\delta}_1, \hat{\gamma}_1 \,|\text{gene locus}) = \text{Cov}(\hat{\delta}_1, \hat{\gamma}_1 \,|\beta_1, R) \tag{26}$$

$$= \begin{cases} 0 & \text{LD contamination} \\ \text{Var}(\gamma_1) & \text{Mediation model} \end{cases} \tag{27}$$

8.6 Concordance of mediated effects for allelic series of independent eQTLs

Under the mediation model in Eq. 8, we expect that for a given gene with multiple QTL signals, these signals should share the same downstream effect, β_g. Since the number of splicing events with multiple QTL signals was limited, we restricted this analysis to eQTLs only. We tested for concordance of downstream effect size obtained from the primary and secondary eQTL of a gene (ranked by QTL significance or QTL effect size estimate). Specifically, for a given trait and gene g, we defined the observed downstream effect for the kth variant as $\hat{\beta}_{k,g} = \hat{\delta}_k/\hat{\gamma}_{k,g}$. Thus, for each gene, we obtained $\hat{\beta}_{prim}$ and $\hat{\beta}_{sec}$ as the observed downstream effect for the primary and secondary eQTLs if more than one eQTL signal was detected by *dap-g*. Ideally, for a mediating gene in a causal tissue (or a good proxy tissue), we would expect that $\hat{\beta}_{prim}$ and $\hat{\beta}_{sec}$ should be similar. We measured the concordance in two ways: 1) correlation between $\hat{\beta}_{prim}$ and $\hat{\beta}_{sec}$; 2) percent concordant, defined as the fraction of eQTL pairs having the same sign in $\hat{\beta}_{prim}$ and $\hat{\beta}_{sec}$. The results of 1) were reported in [The GTEx Consortium, 2020].

Visualizing the concordance among colocalized genes. To visualize the concordance of $\hat{\beta}_{prim}$ and $\hat{\beta}_{sec}$, we first scaled $\hat{\delta}$ and $\hat{\gamma}$ by their standard deviation among all eQTLs selected in Section 8.1.

Then, we extracted the set of genes with at least two *dap-g* eQTLs (defined in 8.1) and labelled the top two eQTLs (rank by QTL effect size magnitude) as primary and secondary based on QTL significance or QTL effect size. We computed $\hat{\beta}_{prim}$ and $\hat{\beta}_{sec}$ and removed the genes with $\hat{\beta}_{prim}$ or $\hat{\beta}_{sec}$ in the top and bottom 5%. As a control, we also simulated random δ to compute simulated β_{sim} for downstream analysis. We further filtered the genes by selecting only those with *enloc* rcp > 0.1.

9 Identifying patterns of regulation of expression across tissues

We used FLASH Sparse Factor Analysis [Wang and Stephens, 2018] to identify latent factors specific to different tissue clusters. We ran `flashr` on a set of top eQTLs (obtained from all GTEx individuals) per gene which had been tested in all 49 tissues (around 16,000 eQTLs in total were selected) and shown strong evidence of being active in at least one tissue. Then, for each selected variant-gene pair, the marginal effect size estimates were extracted for all 49 tissues regardless of whether it was significant in that tissue or not. The resulting estimated effect-size matrix (of dimension $\sim 16,000 \times 49$) was the input to `flashr` (with normal prior on loading and uniform with positive support as prior on factor) to obtain the sparse factors. The `flashr` run yielded 31 FLASH factors (Fig. S13), which were used to assign the tissue-specificity of an eQTL.

We defined the eQTL cross-tissue patterns by projecting the estimated effect-size vector across 49 tissues onto the FLASH factors and computed the quality of the projection, PVE, as PVE$_k = \frac{\|\vec{\beta}_k\|_2^2}{\|\vec{\beta}\|_2^2}$. PVE$_k$ represented the quality score for using FLASH factor k to explain the cross-tissue pattern of eQTL. The eQTL was assigned to a FLASH factor k if PVE$_k$ was maximal among all FLASH factors and PVE$_k > 0.2$ and for those with PVE$_k \leq 0.2$ in all FLASH factors, `NA` (short for not assigned) was assigned instead. These "not assigned" eQTLs had more complex tissue-sharing pattern than the factors captured in the FLASH analysis. To obtain an interpretable tissue-specificity category, we labeled *Factor1* as the shared factor, *Factor2*, *Factor13*, *Factor14*, *Factor29*, and *Factor30* as brain-specific factors, and the rest of the factor assignment as *other factors*.

We applied the multivariate adaptive shrinkage implemented in *mashr* [Urbut et al., 2018] to smooth cis-eQTL effect size estimates (obtained from all GTEx individuals) by taking advantage of correlation between tissues. To fit the *mashr* model, we used the set of $\sim 16,000$ cis-eQTLs as stated in Section 9 to learn the *mashr* prior, and then fit the *mashr* model using $\sim 40,000$ randomly selected variant-gene pairs for the same set of eGenes. We learned data-driven *mashr* priors in three ways: 1) FLASH factors as described above; 2) PCA with number of PC = 3; 3) empirical covariance of observed z-scores. The data-driven covariances were further denoised by calling `cov_ed` in `mashr`. Furthermore, we included the set of canonical covariances as described in [Urbut et al., 2018] as an additional *mashr* prior. We fit the *mashr* model using the set of randomly selected variant-gene pairs with the error correlation estimated by applying `estimate_null_correlation` function in `mashr` and the priors obtained above. The resulting *mashr* model was used to compute the posterior mean, standard deviation, and local false sign rate (LFSR) for any variant-trait pair.

10 Causal gene prioritization

Two classes of methods can be used to identify the target genes of GWAS loci. One class is based on the colocalization of GWAS and QTL loci, which seeks to determine whether the causal variant for the trait is the same as the causal variant for the molecular phenotype. The other class is based on the association between the genetically regulated component of gene expression (or splicing) with the trait. We applied representative examples of each class of methods.

10.1 Colocalization

For a given variant associated with multiple traits such as gene expression (eQTL) and complex disease (trait-associated variant), extensive LD makes it challenging to identify the underlying true causal mechanisms. Colocalization approaches attempt to address this problem. Here, we conducted colocalization analysis using two independent approaches: *coloc* [Giambartolomei et al., 2014] and *enloc* [Wen et al., 2017]), to estimate whether a gene's expression or a splicing event shares a causal variant with a trait.

10.2 enloc

We computed Bayesian regional colocalization probability (rcp) using *enloc*, to estimate the probability of a GWAS region and a gene's cis window sharing causal variants. We used the *dap-g* results described in 7, which was based on EUR individuals only. We split the GWAS summary statistics into approximately LD-independent regions [Berisa and Pickrell, 2016], each region defining a GWAS locus. For each tissue-trait combination, we computed the rcp of every overlapping GWAS locus to a gene's or splicing event's cis window with *enloc*'s default execution mode.

For each trait, we counted the number of GWAS loci that contain a GWAS significant hit, and among these, the number of loci that additionally contain a gene with *enloc* colocalization $rcp > 0.5$. As shown in Fig. A4C, across traits, a median 29% of loci with a GWAS signal contain an *enloc* colocalized signal. Given *enloc*'s conservative nature, we caution that $rcp < 0.5$ does not mean that there is no causal relationship between the molecular phenotype and the complex trait; rather, it should be interpreted as lack of sufficient evidence with current data. We summarize the findings in Fig. A5. We observed a smaller proportion of GWAS loci containing a colocalized splicing event (median 11% across traits).

10.3 coloc

We computed *coloc* on all cis-windows with at least one eVariant (cis-eQTL per-tissue q-value< 0.05) or sVariant. For each gene's cis-window, we used summary statistics from the GWAS traits and the main GTEx eQTL/sQTL analysis. For binary traits, case proportion and 'cc' trait type parameters were used. For continuous traits, sample size and 'quant' trait type parameters were used. In both cases, imputed or calculated z-scores were used as effect coefficients in Bayes factor calculations.

Coloc is very sensitive to the choice of priors. We used *enloc*'s enrichment estimates to define data-based priors in a consistent manner. First, we defined likely LD-independent blocks of variants using definitions provided previously [Berisa and Pickrell, 2016]. The probability of eQTL signal, $\Pr(d_i = 1)$, was estimated using *dap-g* [Wen, 2016]. Subsequently, we calculated priors p_1, p_2, and p_{12} for colocalization analyses as follows:

$$p_1 := \Pr(\gamma_i = 1, d_i = 0) = \frac{\exp(\alpha_0)}{1 + \exp(\alpha_0)} \times (1 - \Pr(d_i = 1)),$$

$$p_2 := \Pr(\gamma_i = 0, d_i = 1) = \frac{1}{1 + \exp(\alpha_0 + \alpha_1)} \times \Pr(d_i = 1), \text{ and}$$

$$p_{12} := \Pr(\gamma_i = 1, d_i = 1) = \frac{\exp(\alpha_0 + \alpha_1)}{1 + \exp(\alpha_0 + \alpha_1)} \times \Pr(d_i = 1),$$

where α_0 and α_1 indicate intercept effect estimate and log odds ratio estimate for the enrichment using *enloc*, respectively.

We ran *coloc* using variants in the cis-window for each gene and the intersection with each GWAS trait, obtaining five probabilities for each gene-tissue-trait tuple: **P0** for the probability of neither expression nor GWAS having a causal variant; **P1** for the probability of only expression having a causal variant; **P2** for only the GWAS having a causal variant; **P3** for the GWAS and expression traits to have distinct causal variants;

P4 for the GWAS and expression traits to have a shared causal variant. We repeated this process using sQTL results.

11 Fine-mapping of height GWAS using summary statistics

To investigate the robustness of fine-mapping, we fine-mapped "height" from the GIANT GWAS meta-analysis and "standing height" from the UK Biobank using `susieR` [Wang et al., 2018]. We performed fine-mapping using `susie_bhat` within each LD block [Berisa and Pickrell, 2016]. We used GWAS effect sizes $\tilde{\beta}$ imputed from z-scores by $\tilde{\beta} = z/\sqrt{Nf(1-f)}$ and se$(\tilde{\beta}) = \tilde{\beta}/z$, where f is allele frequency and N is GWAS sample size. The GTEx-EUR individuals were used to calculate the reference LD panel. We recorded 95% credible set which has posterior probability 95% to capture a causal signal. To compare the fine-mapping results of two GWASs, we defined their 95% credible sets as "overlapped" if they shared at least one variant. To see how 95% in GIANT GWAS is colocalized with UK Biobank GWAS, we calculated colocalization probability as $\sum_{i:\text{variant}_i \in 95\%\text{CS of GIANT}} \text{PIP}_{i,\text{GIANT}} \times \text{PIP}_{i,\text{UKB}}$.

12 Association to predicted expression or splicing

12.1 Predicting the genetically regulated components of expression and splicing

To predict expression, we constructed linear prediction models [Barbeira et al., 2020], using only individuals of European ancestry, and variants with MAF > 0.01, for genes annotated as protein-coding, pseudo-gene, or lncRNA. For each gene-tissue pair, we selected the variants with highest *pip* in their cluster, and kept those achieving pip > 0.01 in *dap-g* [Wen, 2016]. We used *mashr* [Urbut et al., 2018] effect sizes (as computed in 9) for each selected variant. For each model, we computed the covariance matrix between variants using only individuals of European ancestries, with sample sizes ranging from 65 (kidney - cortex) to 602 (skeletal muscle). This allowed us to build LD panels for every tissue. For every gene, we also computed the covariance of all the variants present across the different tissue models, compiling a cross-tissue LD panel to compute the correlation between predicted expression levels across tissues. **We refer to these models as fine-mapped-*mashr* models**. We compared the number of *mashr* models to the number of Elastic Net models from GTEx version 7 (Fig. S5). We generated analogous prediction models for splicing ratios, as computed by Leafcutter [Li et al., 2018], applying the same model-building methodology to the data from the sQTL analysis.

Expression phenotypes were adjusted for unwanted variation using the following covariates: sex, sequencing plaform, the top 3 principal components from genotype data, and PEER factors. The number of PEER factors was determined from the sample size: 15 for $n < 150$, 30 for $150 \leq n < 250$, 45 for $250 \leq n < 350$, 60 for $350 \leq n$. We obtained 686,241 models for different (gene, tissue) pairs.

We also generated analogous prediction models for splicing ratios, with the same model-building methodology applied to the data from the sQTL analysis, obtaining 1,816,703 (splicing event, tissue) pairs.

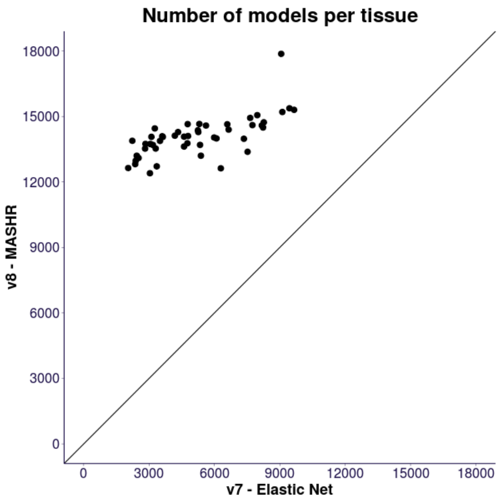

Fig S5. Number of models available in v8 fine-mapped-*mashr* family of models, compared to v7 Elastic Net family. The point with 17,867 models is Testis, consistently with the high levels of expression observed in the eQTL analysis The GTEx Consortium [2020].

12.2 PrediXcan

We performed PrediXcan analysis [Barbeira et al., 2018] on the 87 complex traits, using the GWAS summary statistics described in 3.2, to identify trait-associated genes (typically $p < 2.5 \times 10^{-7}$). We used the 49 models and LD panels described in 12.1, separately on each trait, to obtain 59,485,548 gene-tissue-trait tuples. Repeating this process to generate splicing event ratio models, we obtained 154,891,730 splicing event-tissue-trait tuples; for each trait, the Bonferroni-significance threshold was $p < 9.5 \times 10^{-8}$.

12.3 Colocalized and significantly associated genes

We assessed how many genes present evidence of trait association and colocalization, using both expression and splicing event. First, we counted the proportion of genes that showed a colocalized expression signal with any trait in any tissue, and observed 15% such genes at rcp> 0.5. Then, for each gene, we considered the splicing event with highest colocalization value in any trait or tissue, and found evidence for 5% at rcp> 0.5.

Then we repeated this process for PrediXcan associations at different signifcance thresholds. About 30% of genes showed a significant PrediXcan association to any trait, and only 8% when filtered for associations with rcp> 0.5. When using the highest splicing association and colocalization value for a gene, these proportions were 20% and 3%, respectively.

These proportions gauge our power to predict causal genes affecting complex traits on the GTEx resource, with expression yielding more findings than splicing.

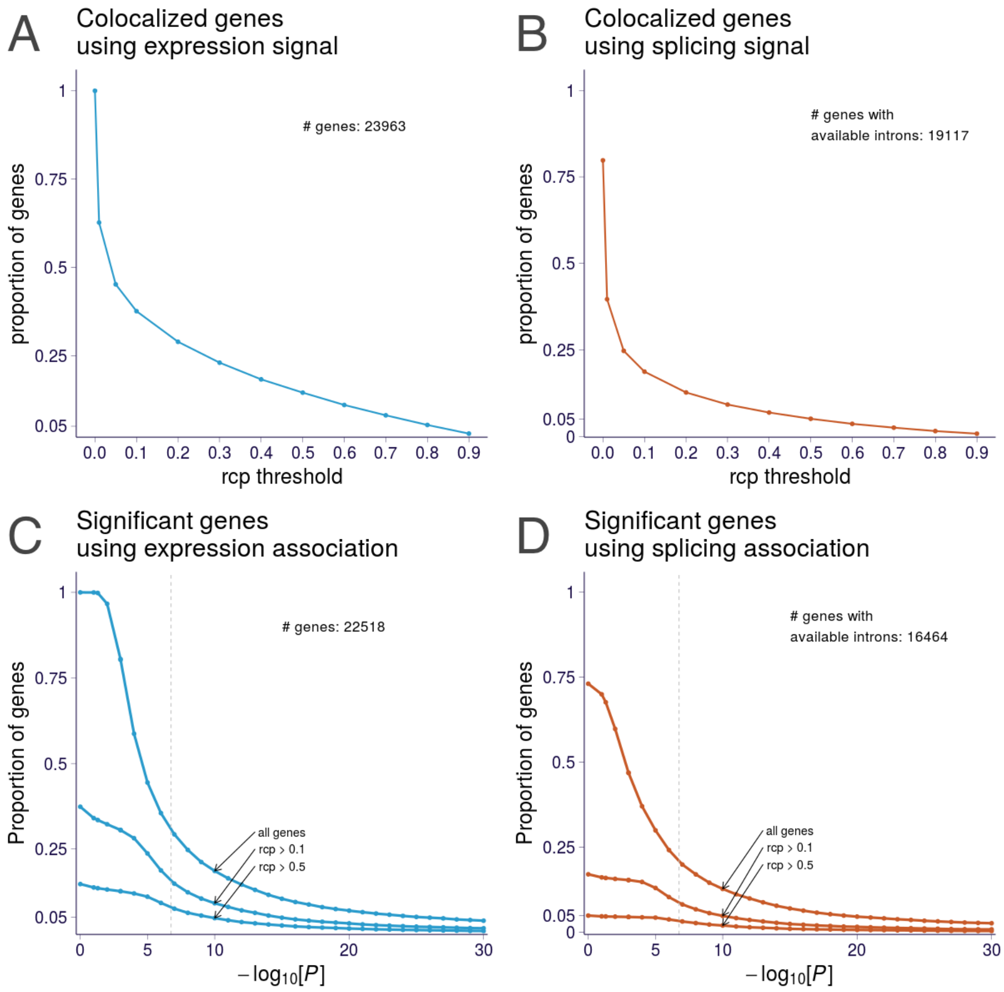

Fig S6. Proportion of genes with a colocalized or associated signal using expression or splicing event.
A shows the proportion of genes with colocalization evidence in expression data, for different rcp thresholds. 3,477 genes show evidence at rcp> 0.5 (15% out of 23,963 genes with *enloc* results).
B shows the proportion of genes with colocalization evidence in splicing data; 1,277 genes (5% of all 23,963) show evidence at rcp> 0.5.
C shows the proportion of genes with association evidence in expression data, additionally filtered by colocalization on different thresholds. About 30% of genes show associations at the bonferroni threshold ($p < 0.05/686,241$), while 8% also show colocalization evidence.
D shows the proportion with association and colocalization evidence in splicing data; about 20% show association evidence ($p < 0.05/1,816,703$) and 3% are also colocalized.

12.4 S-MultiXcan

Given the substantial sharing of eQTLs across tissues [GTEx Consortium et al., 2017], we aggregated PrediXcan results across tissues using S-MultiXcan [Barbeira et al., 2019]. MultiXcan has been shown to exploit the tissue sharing of regulatory variation, to improve our ability to identify trait-associated genes. The method extends the single-tissue PrediXcan approach, leveraging GWAS summary statistics and taking into account the correlation between tissues. We obtained association statistics for 1,958,220 gene-trait pairs and 11,986,329 splicing event-trait pairs.

Additional Table A1: BioVU table. (See Additional file 5) Columns are: **gene**, **tissue**, **trait_map**: mapped trait, **pheno**: trait, **gene_name**, **p_discovery**, **rcp_discovery**, **beta_biovu**, **p_biovu**, **z_biovu**.

12.5 PrediXcan replication in BioVU

We replicated the significant gene-level associations for a prioritized list of traits (Additional Table A1) using BioVU [Denny et al., 2013], Vanderbilt University's DNA Biobank tied to a large-scale Electronic Health Records (EHR) database. We sought BioVU replication in the exact discovery tissues for the significant gene-trait associations. We restricted our analysis to subjects of European ancestries, using principal component analysis as implemented in EIGENSOFT (version 7.1.2; [Price et al., 2006]). First, we estimated the genetically determined component of gene expression in the BioVU individuals using the PrediXcan imputation models. We then conducted association analysis for the prioritized traits using logistic regression, with sex and age as covariates.

Among replicated loci are *SORT1* (liver, coronary artery disease rcp = 0.952; dicovery p = 2.041×10^{-19} BioVU p = 3.475×10^{-4}), which has a well-established associations to lipid metabolism and cardiovascular traits [Musunuru et al., 2010]. Chromosome 6p24 region, which contains *PHACTR1*, has been previously associated with a constellation of vascular diseases, including coronary artery disease [Nikpay et al., 2015] and migraine headache [Anttila et al., 2013]. Notably, *PHACTR1* was significant in three different arteries (aorta artery, coronary artery and tibial artery) in two traits (coronary artery disease and migraine) in the replication analysis. In all six tissue-trait pairs, *PHACTR1* showed very high posterior probabilities in discovery analyses (rcp = 0.992 to 1.00). In our replication analysis, *PHACTR1* remained significant only for coronary artery disease associations (Additional Table A1, aorta artery, discovery p = 2.246×10^{-39}, BioVU p = 7.484×10^{-8}; coronary artery, discovery p = 1.952×10^{-37}, BioVU p = 2.047×10^{-7}; tibial artery, discovery p = 1.559×10^{-33}, BioVU p = 9.880×10^{-7}).

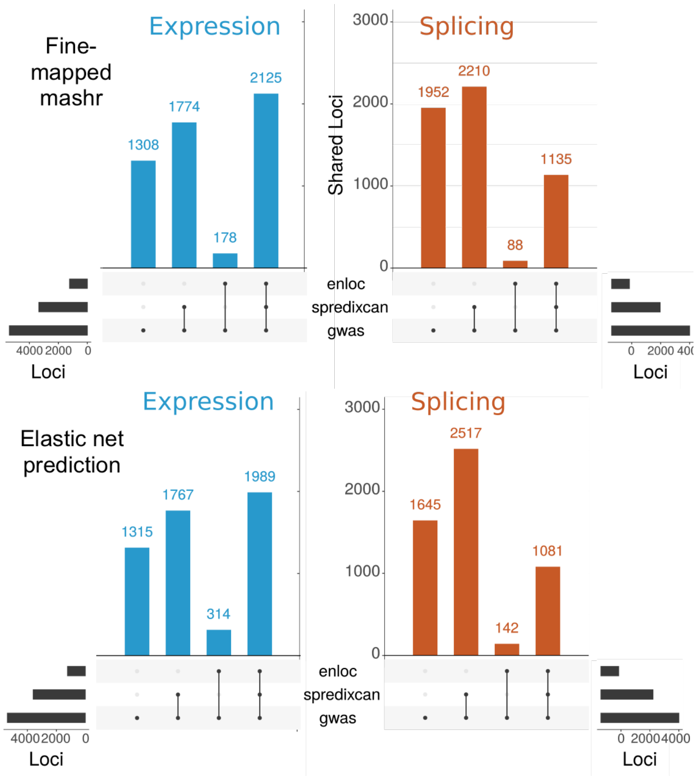

Additional Fig. A3: Causal gene prioritization using PrediXcan and *enloc*.
(A) Fine-mapped-*mashr* model predictions: summary of GWAS loci that also contain an associated PrediXcan or colocalized signal, for expression (left) and splicing (right).
Significance was defined at Bonferroni-adjusted threshold for number of tests in each trait: $p < 0.05/(\text{gene-tissue pairs}) = 7.28 \times 10^{-8}$ for expression, $p < 0.05/(\text{intro-tissue pairs}) = 2.75 \times 10^{-8}$ for splicing. Colocalization status was defined as *enloc* rcp> 0.5.
(B) Elastic net predictions: summary of GWAS loci that also contain an associated PrediXcan or *enloc* signal, for expression (left) and splicing (right), using Elastic Net models.
Significance was defined at Bonferroni-adjusted threshold for number of tests in each trait: $p < 0.05/(\text{gene-tissue pairs}) = 1.77 \times 10^{-7}$ for expression, $p < 0.05/(\text{intro-tissue pairs}) = 9.51 \times 10^{-8}$ for splicing. Colocalization status was defined as *enloc* rcp> 0.5.

The number of loci with potential target genes according to both S-PrediXcan and enloc went up from 1989 with Elastic Net models to 2125 with the improved fine-mapped-*mashr* models. The number of S-PrediXcan backed loci increased only 7, so that the added loci were mostly due to an increased overlap with enloc.

Additional Table A2: Expression and splicing prediction models using fine-mapped-*mashr* models. Training sample size and number of genes predicted for expression and splicing traits.

name	european samples	abbreviation	expression models	splicing models
Adipose - Subcutaneous	491	ADPSBQ	14732	42912
Adipose - Visceral (Omentum)	401	ADPVSC	14640	41720
Adrenal Gland	200	ADRNLG	13622	36754
Artery - Aorta	338	ARTAORT	14396	40474
Artery - Coronary	180	ARTCRN	13878	40579
Artery - Tibial	489	ARTTBL	14493	40690
Brain - Amygdala	119	BRNAMY	12814	24236
Brain - Anterior cingulate cortex (BA24)	135	BRNACC	13528	28806
Brain - Caudate (basal ganglia)	172	BRNCDT	14118	32127
Brain - Cerebellar Hemisphere	157	BRNCHB	13771	39862
Brain - Cerebellum	188	BRNCHA	13992	40747
Brain - Cortex	184	BRNCTXA	14284	35086
Brain - Frontal Cortex (BA9)	158	BRNCTXB	14091	32031
Brain - Hippocampus	150	BRNHPP	13526	27437
Brain - Hypothalamus	157	BRNHPT	13741	30326
Brain - Nucleus accumbens (basal ganglia)	181	BRNNCC	14062	32670
Brain - Putamen (basal ganglia)	153	BRNPTM	13694	28461
Brain - Spinal cord (cervical c-1)	115	BRNSPC	13096	28883
Brain - Substantia nigra	101	BRNSNG	12637	23677
Breast - Mammary Tissue	337	BREAST	14654	44613
Cells - Cultured fibroblasts	417	FIBRBLS	13976	36809
Cells - EBV-transformed lymphocytes	116	LCL	12398	37627
Colon - Sigmoid	274	CLNSGM	14363	41581
Colon - Transverse	306	CLNTRN	14582	41215
Esophagus - Gastroesophageal Junction	281	ESPGEJ	14285	41004
Esophagus - Mucosa	423	ESPMCS	14589	37186
Esophagus - Muscularis	399	ESPMSL	14603	40376
Heart - Atrial Appendage	322	HRTAA	14035	36322
Heart - Left Ventricle	334	HRTLV	13200	29470
Kidney - Cortex	65	KDNCTX	11164	24571
Liver	183	LIVER	12714	27011
Lung	444	LUNG	15058	44346
Minor Salivary Gland	119	SLVRYG	13884	38380
Muscle - Skeletal	602	MSCLSK	13381	31855
Nerve - Tibial	449	NERVET	15373	45478
Ovary	140	OVARY	13738	40857
Pancreas	253	PNCREAS	13695	31203
Pituitary	219	PTTARY	14647	42343
Prostate	186	PRSTTE	14450	41991
Skin - Not Sun Exposed (Suprapubic)	440	SKINNS	14932	42005
Skin - Sun Exposed (Lower leg)	517	SKINS	15204	42219
Small Intestine - Terminal Ileum	144	SNTTRM	14065	39864
Spleen	186	SPLEEN	14073	40290
Stomach	269	STMACH	14102	36624
Testis	277	TESTIS	17867	67784
Thyroid	494	THYROID	15303	45217
Uterus	108	UTERUS	13199	39485
Vagina	122	VAGINA	12969	36931
Whole Blood	573	WHLBLD	12623	24568
total			686241	1816703

Table S3: GWAS loci with colocalized or significant genes assigned. Numbers of loci-trait associations with associated/colocalized genes/splicing event detected by each method. A locus is said to have a GWAS association to a trait if it contains at least one variant with $p < 0.05/(\text{variants tested}) = 5.7 \times 10^{-9}$. We list here how many such loci-trait associations have an S-PrediXcan association or *enloc* signal. Significant S-PrediXcan associations were defined at Bonferroni-adjusted threshold for number of tests in each trait: $p < 0.05/(\text{gene-tissue pairs}) = 7.28 \times 10^{-8}$ for expression, $p < 0.05/(\text{intro-tissue pairs}) = 2.75 \times 10^{-8}$ for splicing. Colocalization status was defined as *enloc* rcp> 0.5.

GWAS-significant (loci, trait) associations		5385
GWAS-significant unique loci		1167
enloc (loci, trait) colocalizations	expression	2303
enloc (loci, trait) colocalizations	splicing	1223
PrediXcan (loci, trait) associations	expression	3899
PrediXcan (loci, trait) associations	splicing	3345
PrediXcan & enloc (loci, trait) detections	expression	2125
PrediXcan & enloc (loci, trait) detections	splicing	1135

12.6 Summary-data-based Mendelian Randomization (SMR) and HEIDI

For comparison, we also performed top-eQTL based Summary-data-based Mendelian Randomization (SMR) [Zhu et al., 2016] analysis of the 4,263 tissue-trait pairs. SMR, which integrates summary statistics from GWAS and eQTL data, has been used to prioritize genes underlying GWAS associations.

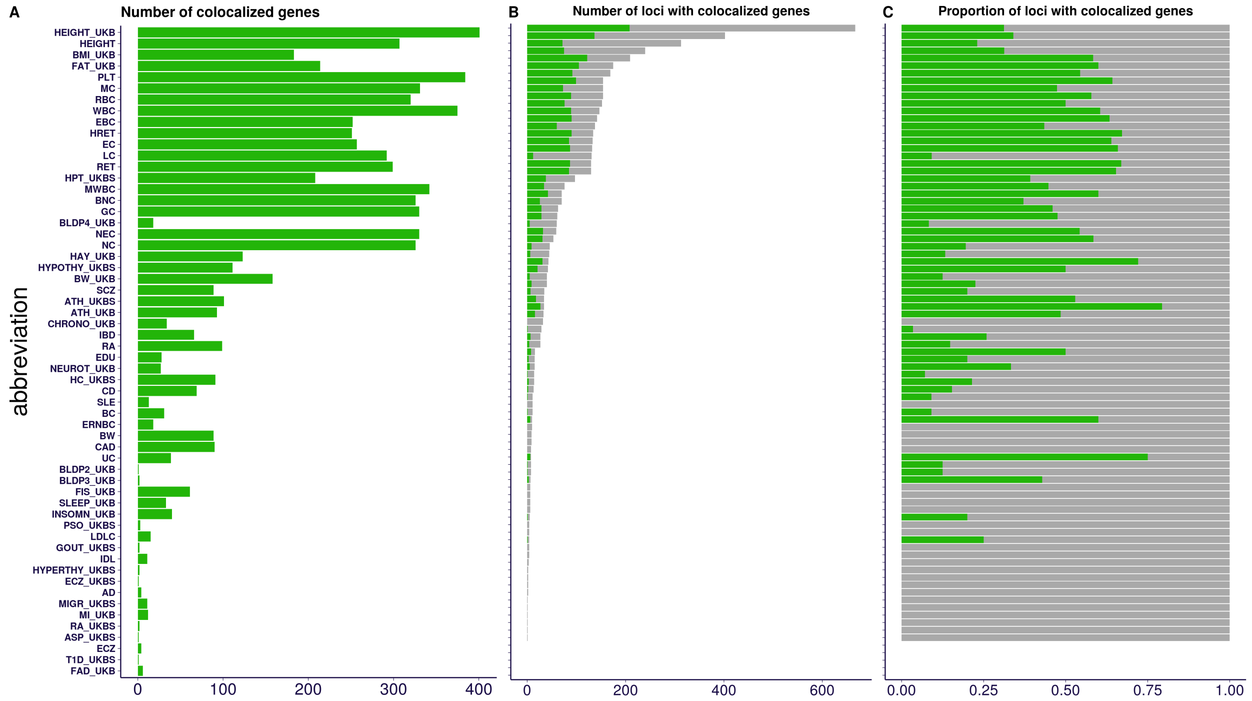

Additional Fig. A4: Colocalization of expression QTLs for the 87 GWAS traits aggregated across the 49 tissues. GWAS loci are shown in gray, colocalized results are shown in dark green. The traits are ordered by number of GWAS-significant variants.

Panel A shows the number of colocalized genes, achieving *enloc* $rcp > 0.5$ in at least one tissue, for each GWAS trait. The number of colocalized results tends to increase with the number of GWAS-significant variants.

Panel B shows the number of loci (approximately independent LD regions from [Berisa and Pickrell, 2016]) with at least one GWAS-significant variant (dark gray), and among them those with at least one gene reaching $rcp > 0.5$ (dark green).

Panel C shows the proportion of loci with at least one GWAS-significant hit that contain at least one colocalized gene. Across traits, a median of 21% of the GWAS loci contain colocalized results. See trait abbreviation list in Table S2. These results were also presented in [The GTEx Consortium, 2020] and are shown here for completeness.

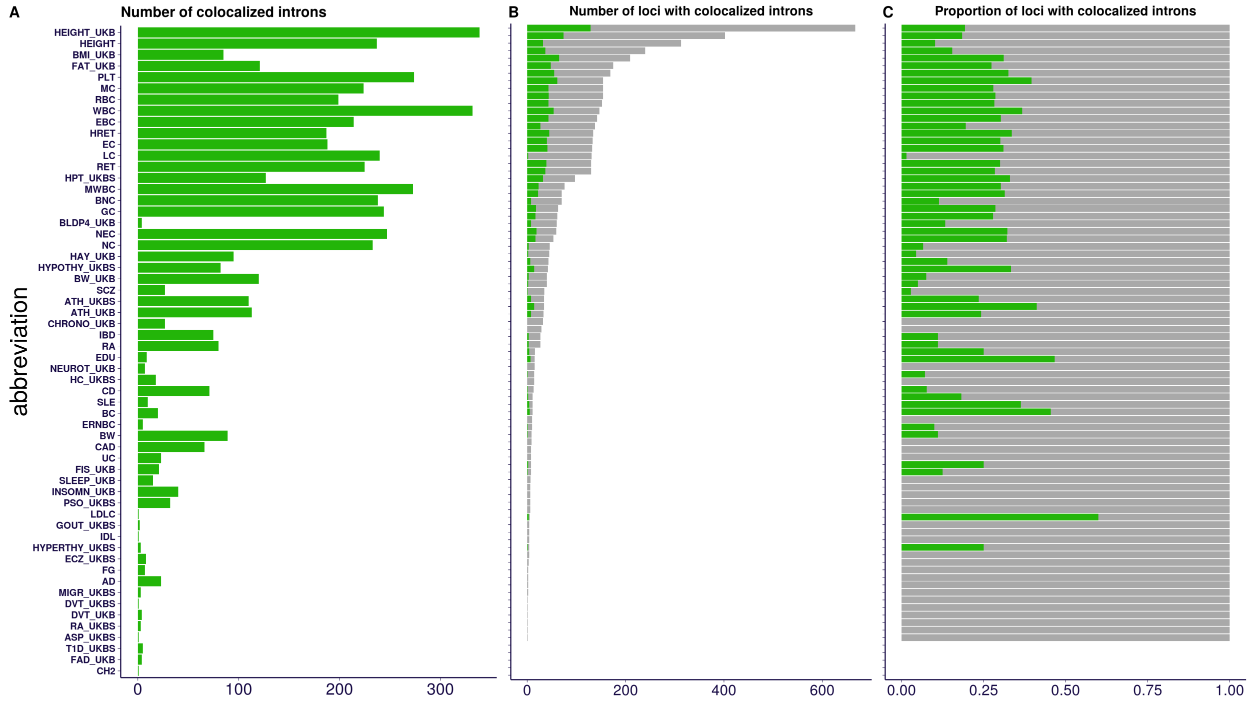

Additional Fig. A5: Colocalization of splicing QTLs for each of the 87 GWAS traits aggregated across the 49 tissues. The traits are ordered by number of GWAS-significant variants. GWAS loci are shown in gray, colocalized results are shown in dark green.

Panel A shows the number of colocalized splicing event, achieving *enloc* $rcp > 0.5$ in at least one tissue, for each GWAS trait. As with gene expression results, the number of colocalized results tends to increase with the number of GWAS-significant variants.

Panel B shows the number of loci (approximately independent LD regions from [Berisa and Pickrell, 2016]) with at least one GWAS-significant variant (dark gray), and among them those with one splicing event achieving $rcp > 0.5$ (dark green).

Panel C shows the proportion of loci with at least one GWAS-significant hit loci with at least one colocalized splicing event. Across traits, a median of 11% of the GWAS loci contain a colocalized result, lower than the gene expression counterpart (29%), indicating a decreased power in the sQTL study. See trait abbreviation list in Table S2.

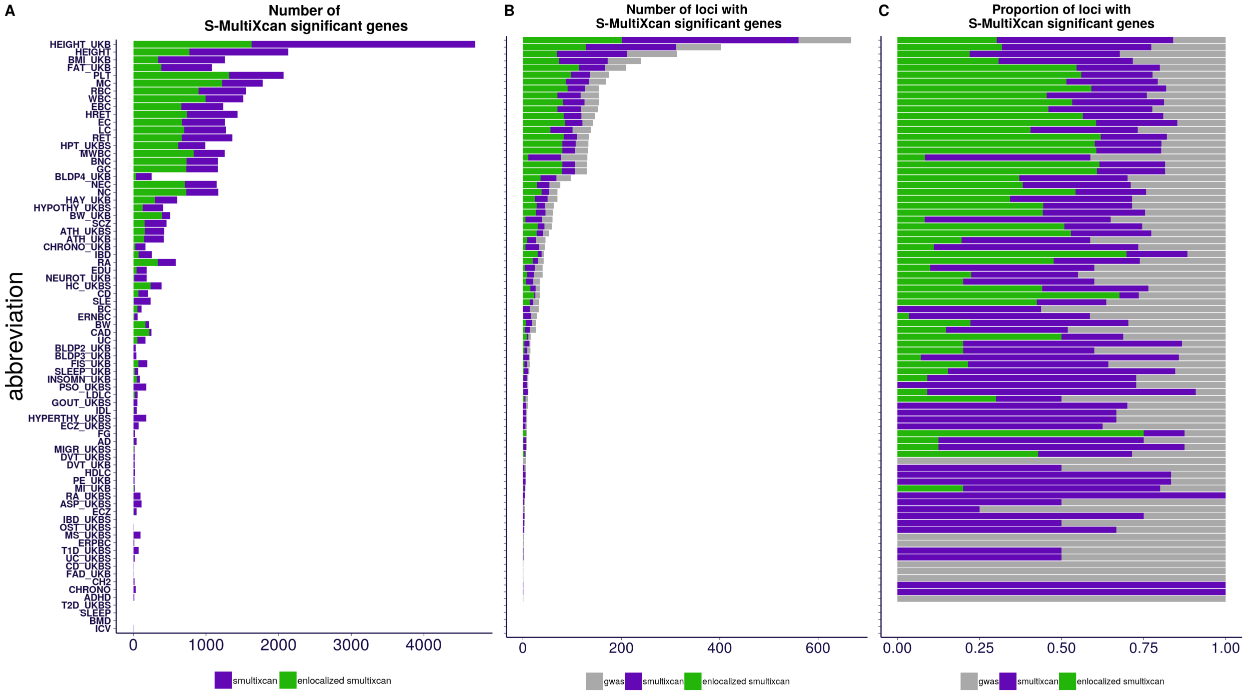

Fig S7. PrediXcan expression associations aggregated across tissues. This figure summarizes S-MultiXcan associations for each of the 87 traits using the gene expression models. The traits are ordered by number of GWAS-significant variants.

Panel A) shows in purple the number of S-MultiXcan significant genes, and in dark green the subset also achieving *enloc* $rcp > 0.5$ in any tissue. S-MultiXcan has a high power for detecting associations, but 12% (median across traits) of these genes show evidence of colocalization.

Panel B) shows the number of loci (approximately independent LD regions [Berisa and Pickrell, 2016]) with a significant GWAS association (gray), a significant S-MultiXcan association (purple), and a significant S-MultiXcan association that is colocalized (dark green). Anthropometric and Blood traits tend to present the largest number of associated loci, with Height from two independent studies leading the number of associations.

Panel C) shows the proportion of loci with significant GWAS associations (gray) that contain S-Multixcan (purple) and colocalized S-MultiXcan associations (dark green). Across traits, a median of 70% of GWAS-associated loci show a S-MultiXcan detection, while 19% show a colocalized S-MultiXcan detection.

See trait abbreviation list in Table S2.

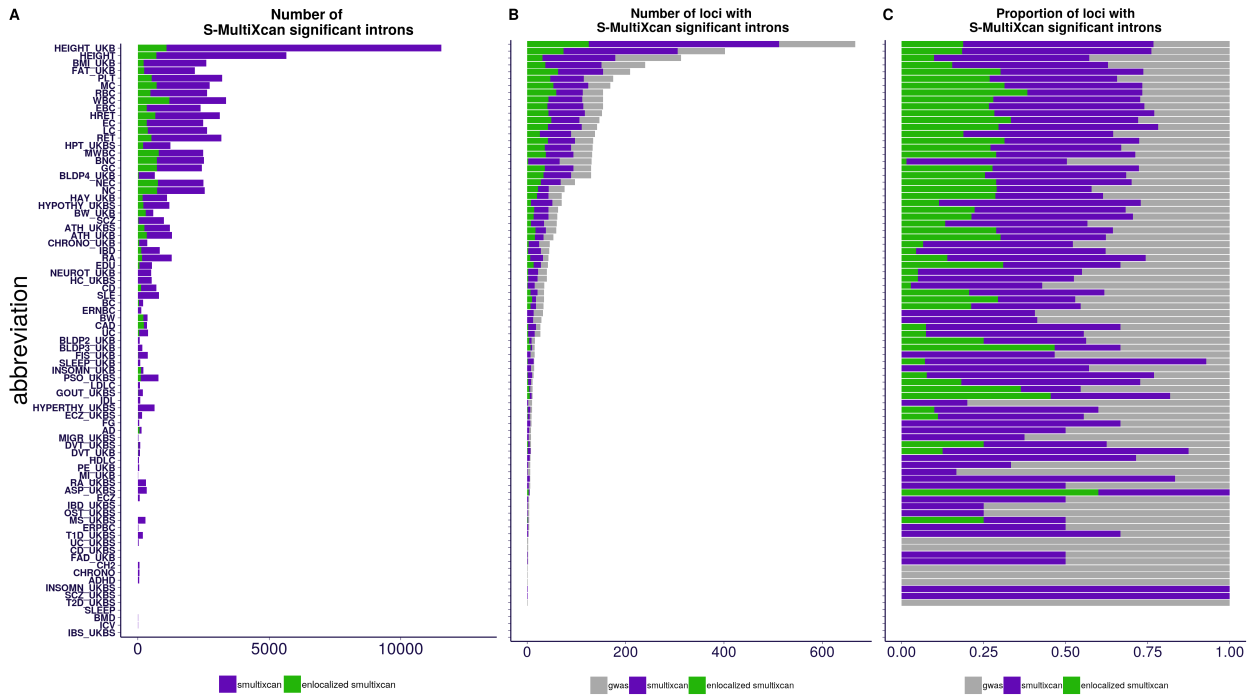

Fig S8. PrediXcan splicing associations aggregated across tissues. This figure summarizes S-MultiXcan associations for each of the 87 traits using splicing models. The traits are ordered by number of GWAS-significant variants.
Panel A) shows in purple the number of S-MultiXcan significant splicing events, and in dark green the subset also achieving *enloc* $rcp > 0.5$ in any tissue. The proportion of colocalized, significantly associated splicing events is typically 2%, much lower than the proportion from gene expression (12%).
Panel B) shows the number of loci (approximately independent LD regions [Berisa and Pickrell, 2016]) with a significant GWAS association (gray), a significant S-MultiXcan association (purple), and a significant S-MultiXcan association that is colocalized (dark green). As in the case of expression models, Anthropometric and Blood traits tend to present the largest number of associated loci.
Panel C) shows the proportion of loci with significant GWAS associations (gray) that contain S-Multixcan (purple) and colocalized S-MultiXcan associations (dark green). Across traits, a median of 63% of GWAS-associated loci show an S-MultiXcan association, while 11% show a colocalized S-MultiXcan association. These proportions are lower than the corresponding ones for expression (70% and 19% respectively).
See trait abbreviation list in Table S2.

13 Assessing the performance of association and colocalization methods to identify causal genes

To assess the performance of colocalization and association methods to identify causal genes, we curated two sets of ‘causal’ gene-trait pairs. One set is based on the OMIM database and the other one is based on rare variant association results from exome-wide association studies. To quantify the performance, we framed the causal gene identification problem as one of classification and used the standard tools such as ROC and precision recall curves, which have the advantage of not needing ad-hoc thresholds and show the full trade-off between true positives and false positives as well as precision vs. power. Throughout this section, we limited our scope to only the protein-coding genes.

13.1 OMIM-based curation of causal genes

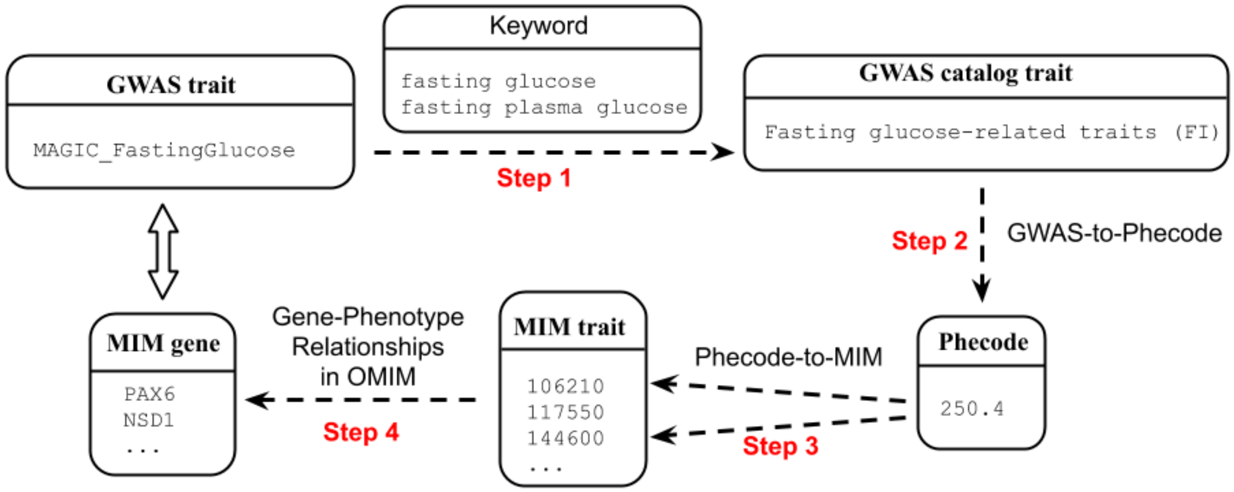

Fig S9. Workflow of OMIM-based curation of causal genes. The workflow of OMIM-based causal gene curation is shown where each box represents the trait description/identifier in different databases. The steps to obtain OMIM genes for `MAGIC_FastingGlucose`, one of our GWAS traits, is shown as a concrete application of the workflow.

Additional Table A3: Keywords of GWAS traits used for mapping with the GWAS catalog. Keywords of all 114 GWAS traits used for OMIM-based curation and analyses are listed.

Abbreviation	Keyword	Abbreviation	Keyword
Sleep_Duration_UKB	sleep duration	Sum_Eosinophil_Basophil_Ct	
Chronotype_UKB	chronotype	Sum_Neutrophil_Eosinophil_Ct	
Insomnia_UKB	insomnia	White_Blood_Cell_Count	white blood cell count
Fathers_Age_At_Death_UKB	aging	Coronary_Artery_Disease	coronary heart disease
Deep_Venous_Thrombosis_UKB	venous thromboembolism	Chronic_Kidney_Disease	chronic kidney
Asthma_UKB	asthma	Insomnia_In_Both_Sexes	insomnia
Irritable_Bowel_Syndrome_UKB	irritable bowel	Type_2_Diabetes	type 2 diabetes
Type_1_Diabetes_UKB	type 1 diabetes	Eczema	atopic dermatitis
Type_2_Diabetes_UKB	type 2 diabetes	Birth_Length	
Hyperthyroidism_UKB	hyperthyroidism	BMI_Childhood	bmi;body mass index
Hypothyroidism_UKB	hypothyroidism	Birth_Weight	
Psychological_Problem_UKB	psychiatric;psychological	Pubertal_Height_Female	
Multiple_Sclerosis_UKB	multiple sclerosis	Pubertal_Height_Male	
Parkinsons_Disease_UKB	Parkinson's	Intracraneal_Volume	intracranial volumn
Migraine_UKB	migraine	Asthma	asthma
Schizophrenia_UKB	Schizophrenia	Bone_Mineral_Density	bone mineral density
Osteoporosis_UKB	osteoporosis	BMI_Active_Inds	bmi;body mass index
Ankylosing_Spondylitis_UKB	ankylosing spondylitis	BMI_EUR	bmi;body mass index
Eczema_UKB	eczema;dermatitis	Height	height
Psoriasis_UKB	psoriasis	Hip_Circumference_EUR	hip circumference
Inflammatory_Bowel _Disease_UKB	inflammatory bowel disease	Waist_Circumference_EUR	waist circumference
Crohns_Disease_UKB	crohn's disease	Waist-to-Hip_Ratio_EUR	waist-to-hip
Ulcerative_Colitis_UKB	ulcerative colitis	HDL_Cholesterol	hdl cholesterol
Rheumatoid_Arthritis_UKB	rheumatoid arthritis	LDL_Cholesterol	ldl cholesterol
Gout_UKB	gout	Triglycerides	triglycerides
High_Cholesterol_UKB	total cholesterol	Neuroticism	neuroticism
Insomnia_UKB	insomnia	Heart_Rate	heart rate
Fluid_Intelligence_Score_UKB	intelligence	Crohns_Disease	crohn's disease
Birth_Weight_UKB	birth weight	Inflammatory_Bowel_Disease	inflammatory bowel disease
Neuroticism_UKB	neuroticism	Ulcerative_Colitis	ulcerative colitis
BMI_UKB	bmi;body mass index	Alzheimers_Disease	alzheimer
Body_Fat_Percentage_UKB	body fat	Epilepsy	epilepsy
Balding_Pattern_2_UKB		Celiac_Disease	celiac disease
Balding_Pattern_3_UKB		Multiple_Sclerosis	multiple sclerosis
Balding_Pattern_4_UKB		Systemic_Lupus_ Erythematosus	systemic lupus erythematosus
Mothers_Age_At_Death_UKB	aging	Stroke	stroke
Standing_Height_UKB	height	Chronotype	chronotype
Heart_Attack_UKB		Sleep_Duration	sleep duration
		Fasting_Glucose	fasting glucose; fasting plasma glucose
Pulmonary_Embolism_UKB		Fasting_Insulin	fasting insulin
Asthma_UKB	asthma	CH2DB_NMR	
Hayfever_UKB		HDL_Cholesterol_NMR	hdl cholesterol
Epilepsy_UKB	epilepsy	Triglycerides_NMR	triglycerides
Migraine_UKB	migraine	LDL_Cholesterol_NMR	ldl cholesterol
Hypertension_UKB	hypertension	Attention_Deficit _Hyperactivity_Disorder	attention deficit hyperactivity disorder
Adiponectin	adiponectin	Autism_Spectrum_Disorder	autism
Eosinophil_Count	eosinophil count	Schizophrenia	schizophrenia
Granulocyte_Count		Rheumatoid_Arthritis	rheumatoid arthritis
High_Light_Scatter _Reticulocyte_Count		Depressive_Symptoms	depression
Lymphocyte_Count	lymphocyte	Education_Years	education
Monocyte_Count	monocyte count;monocytes	Asthma_TAGC_EUR	asthma
Myeloid_White_Cell_Count		Systolic_Blood_Pressure	systolic blood pressure
Neutrophil_Count	neutrophil count;neutrophils	Diastolic_Blood_Pressure	diastolic blood pressure
Platelet_Count	platelet counts	ER-negative_Breast_Cancer	breast cancer
Red_Blood_Cell_Count	red blood cell count	ER-positive_Breast_Cancer	breast cancer
Reticulocyte_Count		Breast_Cancer	breast cancer
Sum_Basophil_Neutrophil_Ct		Smoker	smoking behavior

To obtain a curated set of trait-gene pairs from the OMIM database [Hamosh et al., 2005], we mapped our GWAS traits to the OMIM traits and linked them to the corresponding genes in the OMIM database. The mapping process is illustrated in Fig. S9 for a specific example GWAS trait, fasting glucose by the MAGIC consortium. First, the GWAS trait was mapped to the GWAS catalog trait names by searching for relevant keywords (defined manually Additional Table A3) in the description field of the GWAS catalog. Second, the GWAS catalog trait names were linked to phecodes using the mapping in the phewas catalog [Denny et al., 2013]. Third, we mapped phecodes to OMIM traits ids (MIM) as described in [Bastarache et al.,

2018]. Finally, in step 4, we mapped OMIM traits to OMIM genes using the OMIM gene to phenotype map (genemap2.txt) in the OMIM database.

Table S4: Presumed causal genes included in the OMIM database. (See Additional file 3) Columns are: **trait**: Tag used for the trait, **pheno_mim**: MIM ID of the phenotype mapped to GWAS trait, **mim**: MIM ID of the corresponding gene, **entry_type**: Entry type in the OMIM database, **entrez_gene_id**: Gene ID based on Entrez database, **gene_name**: Official gene symbol, **ensembl_gene_id**: Gene ID based on Ensembl database, **gene_type**: Gene type based on Gencode, **gene**: Trimmed Gene ID based on Ensembl database.

The keywords used for the each of the initial selected set of 114 GWAS traits is listed in Additional Table A3). For a subset of datasets with GWAS results from more than one source (public GWAS vs UKB) in our collection, we kept the dataset with higher number of GWAS loci to avoid double counting. The number of GWAS loci was determined based on counting the lead variants, using the PLINK V1.9 command `-clump-r2 0.2 -clump-p1 5e-8` at genome-wide significance 5×10^{-8}) for each trait. Furthermore, for this analysis, we excluded GWAS traits with fewer than 50 GWAS loci. The full list of OMIM based trait-gene pairs is listed in Additional Table S4.

With this procedure, we curated a list of 1,592 gene-trait pairs with evidence of causal associations in the OMIM database (hereafter, **OMIM genes**), which was downloaded from omim.org/downloads (accessed on Aug 12th 2019). After matching traits, we retained 29 unique traits and 631 unique genes that were within the same LD block [Berisa and Pickrell, 2016] as the GWAS hit (Additional Table S4).

13.2 Rare variant association-based curation of causal genes

In addition to the OMIM-based curation, we collected a set of genes in which rare protein-coding variants were reported to be significantly associated with our list of complex traits. Given the power of existing rare variant association studies, we focused on height and lipid traits (low-density lipid cholesterol, high-density lipid cholesterol, triglycerides, and total cholesterol levels) [Marouli et al., 2017; Liu et al., 2017; Locke et al., 2019].

Table S5: Genes suggested as causal by rare variant association studies. (See Additional file 4) Columns are: **gene**: Trimmed gene ID based on Ensembl database, **nobs**: Number of times gene has been observed in the trait, **trait**: Tag for the trait name.

We collected significant coding/splicing variants reported previously [Marouli et al., 2017] and kept variants with effect allele frequency < 0.01 (table S6 therein: ExomeChip variants with Pdiscovery <2e-07 in the European-ancestry meta-analysis (N=381,625)). Similarly, we collected significant variants reported by [Liu et al., 2017] (table S12 therein: Association Results for 444 independently associated variants with lipid traits) and filtered out variants with minor allele frequency < 0.01. For the whole-exome sequencing study conducted in Finnish isolates [Locke et al., 2019], we extracted significant genes identified by a gene-based test using protein truncating variants (table S9 therein: Gene-based associations from aggregate testing with EMMAX SKAT-O with P<3.88E-6) and significant variants (table S7 therein: A review of all variants that pass unconditional threshold of P<5E-07 for at least one trait) with gnomAD MAF < 0.01. The full list of trait-gene pairs constructed from the process is available in Additional Table S5.

13.3 Setting up the classification problem to quantify performance for identifying causal genes

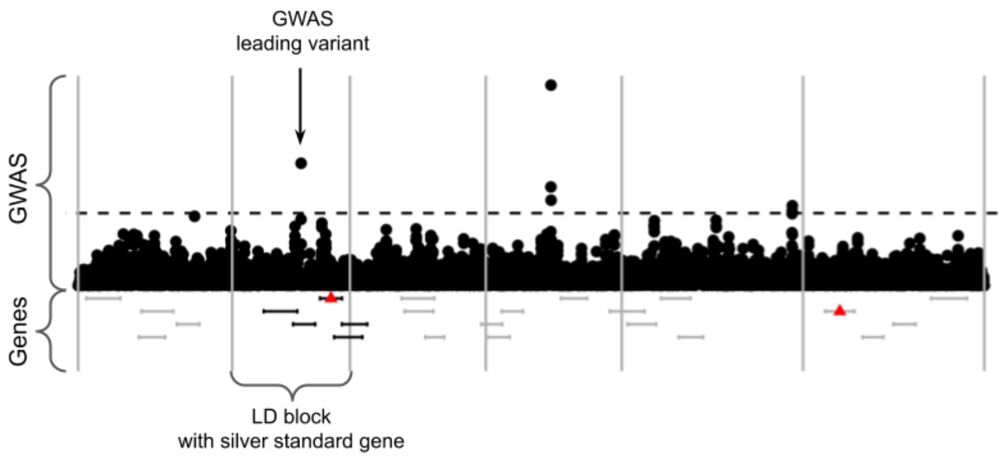

Fig S10. Selection of genes to assess ability to identify silver standard genes. The GWAS summary statistics were binned into independent LD blocks (boundaries of LD block are shown as gray vertical lines). Only genes within LD blocks that contain both a silver standard gene (red triangle) and a GWAS significant variant (points above $-\log_{10}(p) > -\log_{10}(5 \cdot 10^{-8})$) were used in the calculation of performance (ROC and PR curves).

trait	gene	LD block	lead SNP	silver std status	enloc rcp	PrediXcan p-value	proximity
trait1	gene1	LD1	SNP1	0	0.01	1E-04	0
trait1	gene2	LD1	SNP1	1	0.30	1E-08	1
trait1	gene3	LD1	SNP1	0	0.02	0.32	2
trait1	gene4	LD1	SNP1	0	0.10	0.01	3
trait1	gene5	LD2	SNP2	0	0.00	0.38	0
trait1	gene6	LD2	SNP2	0	0.00	0.26	1

Additional Fig. A6: Schematic representation of data used for classification.

We partitioned the genome into approximately independent LD blocks [Berisa and Pickrell, 2016] and for each GWAS trait, we kept only genes located in LD blocks where there were at least one silver standard gene and a GWAS significant hit for the trait as illustrated in Fig. S10. Then, we labelled the silver standard genes as 1 and all the others were labelled as 0, as represented schematically in Fig. A6.

We calculated the ROC and precision recall curves for classifying the silver standard gene correctly. Note that we used a universal cutoff across all GWAS loci, hence highly correlated genes would be classified as causal or non-causal as a cluster.

In more detail, for each of tested gene-trait pairs, we obtained the gene-level statistics for the corresponding trait from the application of various methods, *i.e.* *enloc*, *coloc*, SMR, and PrediXcan-*mashr*. Since we had results across tissues, we selected the 'best' scores (highest regional colocalization probability (rcp) in *enloc*; highest posterior probability under hypothesis 4 in *coloc*; smallest p-value in SMR

and PrediXcan-*mashr*) to build the Table A6. For splicing (with statistics reported at the intron excision event level), we obtained gene-level statistics by taking the 'best' score among all splicing events of the gene, across all tissues.

Additional Table A4: OMIM genes included in the analysis. (See Additional file 6) Columns are: **gene**, **trait.**

Additional Table A5: Rare variant silver standard genes included in the analysis. (See Additional file 7) Columns are: **gene**, **trait.**

The full list of silver standard genes that included in the analysis (after intersecting with GWAS loci) can be found in Additional Table A4 and Additional Table A5.

silver standard	trait	nloci	ngene	silver standard	trait	nloci	ngene
rare variant	Standing_Height_UKB	29	35	OMIM	Monocyte_Count	1	1
rare variant	LDL_Cholesterol	7	10	OMIM	Neutrophil_Count	14	17
rare variant	High_Cholesterol_UKBS	6	8	OMIM	White_Blood_Cell_Count	16	17
rare variant	HDL_Cholesterol	12	18	OMIM	Coronary_Artery_Disease	12	13
rare variant	Triglycerides	6	9	OMIM	Type_2_Diabetes	11	12
OMIM	Deep_Venous_Thrombosis	2	2	OMIM	Waist_Circumference_EUR	6	6
OMIM	Asthma_UKBS	10	12	OMIM	LDL_Cholesterol	7	9
OMIM	Type_1_Diabetes_UKBS	1	2	OMIM	Triglycerides	11	11
OMIM	Hypothyroidism_UKBS	14	14	OMIM	Inflammatory_Bowel_Disease	7	8
OMIM	Eczema_UKBS	4	5	OMIM	Ulcerative_Colitis	4	4
OMIM	Psoriasis_UKBS	2	2	OMIM	Alzheimers_Disease	2	2
OMIM	Gout_UKBS	1	1	OMIM	Systemic_Lupus_Erythematosus	3	5
OMIM	High_Cholesterol_UKBS	6	8	OMIM	Schizophrenia	1	1
OMIM	BMI_UKB	35	35	OMIM	Rheumatoid_Arthritis	3	3
OMIM	Hypertension_UKBS	19	24	OMIM	Systolic_Blood_Pressure	2	2
OMIM	Eosinophil_Count	7	7	OMIM	Diastolic_Blood_Pressure	3	3
OMIM	Lymphocyte_Count	2	2				

Additional Table A6: Count of GWAS loci with predicted causal effects overlapping likely functional genes. The number of GWAS loci and the number of silver standard genes included for analysis after taking the intersection between GWAS loci and silver standard genes are shown.

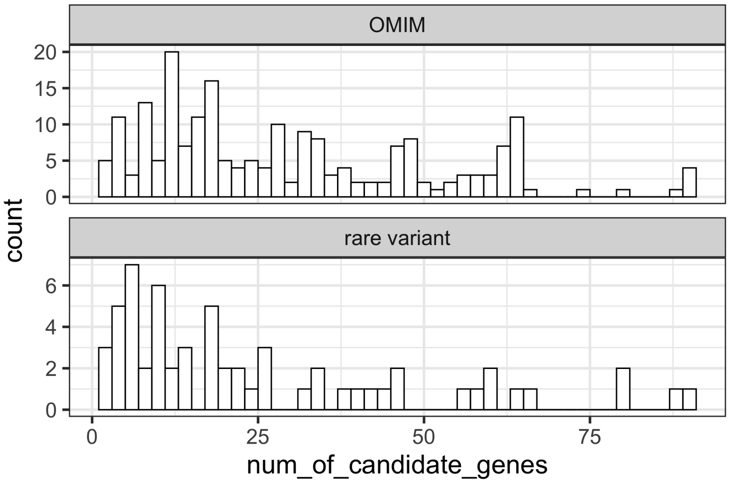

Additional Fig. A7: Distribution of the number of tested genes per GWAS locus overlapping OMIM- and rare variant-based silver standard. The distributions of the number of candidate genes per GWAS locus are shown for OMIM-based curation (top) and rare variant association-based curation (bottom).

The number of GWAS loci and silver standard genes that remained after the above filtering steps can be found in Table A6. The number of genes tested per LD block is shown in Fig. A7.

Additional Table A7: PrediXcan and enloc results for predicted causal genes selected based on OMIM. (See Additional file 8) Columns are: **lead_var**: the most significant variant within the LD block, **trait**: trait name, **gene**: Ensembl ID for the gene, **is_omim**: Is included in the OMIM database. TRUE if included, FALSE if not, **proximity**: 0 if variant is in the gene, otherwise BPS from the gene boundary, **rank_proximity**: ranking by proximity within LD block (rank starts from 0 and the closer the lower rank), **percentage_proximity**: rank_proximity / number of genes in the locus, **predixcan_mashr_eur_score**: -log10 p-value (most significant across tissues is used) of PrediXcan-MASH trained on European data, **enloc_score**: rcp (max across tissues), **predixcan_mashr_eur_rank**: PrediXcan significance ranking within LD block (rank starts from 0 and the higher significance the lower rank), **enloc_rank**: enloc rcp ranking within LD block (rank starts from 0 and the higher rcp the lower rank), **predixcan_mashr_eur_percentage**: predixcan_mashr_eur_rank / number of genes in the locus, **enloc_percentage**: enloc_rank / number of genes in the locus, **gene_name**: Official gene symbol, **gene_type**: Gencode annotsted gene type, **chromosome**: Chromosome for the gene, **start**: Gencode annotated gene start position. All isoforms are combined, **end:** Gencode annotated gene end position. All isoforms are combined, **strand**: Gencode annotated gene strand.

Additional Table A8: PrediXcan and enloc results for presumed causal genes in the rare variant based silver standard. (See Additional file 9) Columns are: **lead_var**: the most significant variant within the LD block, **trait**: trait name, **gene**: Ensembl ID for the gene, **is_ewas**: Is included in the EWAS . TRUE if included, FALSE if not, **proximity**: 0 if variant is in the gene, otherwise BPS from the gene boundary, **rank_proximity**: ranking by proximity within LD block (rank starts from 0 and the closer the lower rank), **percentage_proximity**: rank_proximity / number of genes in the locus, **predixcan_mashr_score**: -log10 p-value (most significant across tissues is used) of PrediXcan-MASH trained on European data, **enloc_score**: rcp (max across tissues), **predixcan_mashr_rank**: PrediXcan significance ranking within LD block (rank starts from 0 and the higher significance the lower rank), **enloc_rank**: enloc rcp ranking within LD block (rank starts from 0 and the higher rcp the lower rank), **predixcan_mashr_percentage**: predixcan_mashr_eur_rank / number of genes in the locus, **enloc_percentage**: enloc_rank / number of genes in the locus, **gene_name**: Official gene symbol, **gene_type**: Gencode annotsted gene type, **chromosome**: Chromosome for the gene, **start**: Gencode annotated gene start position. All isoforms are combined, **end**: Gencode annotated gene end position. All isoforms are combined, **strand**: Gencode annotated gene strand.

And the resulting data tables are Table A7 and Table A8.

13.4 AUC of the ROC curves

For expression, the areas under the curve (AUC) of were, in increasing performance, 0.553, 0.591, 0.669, and 0.672 for *coloc*, SMR, *enloc*, and PrediXcan using the OMIM silver standard (Figure 4C). AUC were higher when using the rare variant silver standard with SMR at the bottom of the ranking followed by *coloc*, PrediXcan, and enloc at the top (Additional Table A9). For splicing *enloc* had higher 0.650 vs. 0.632 for PrediXcan using OMIM silver standard and 0.714 and 0.686 using the rare variant silver standard.

Regulation	Dataset	Method	ROC AUC
expression	OMIM	*coloc*	0.553
expression	OMIM	*enloc*	0.669
expression	OMIM	PrediXcan	0.672
expression	OMIM	SMR	0.591
expression	Rare variant	*coloc*	0.661
expression	Rare variant	*enloc*	0.755
expression	Rare variant	PrediXcan	0.743
expression	Rare variant	SMR	0.629
splicing	OMIM	*enloc*	0.650
splicing	OMIM	PrediXcan	0.632
splicing	Rare variant	*enloc*	0.714
splicing	Rare variant	PrediXcan	0.686

Additional Table A9: Enrichment and AUC for *coloc*, *enloc*, SMR, and PrediXcan

13.5 Precision-recall curves of PrediXcan and *enloc* on the OMIM-based silver standard gene set

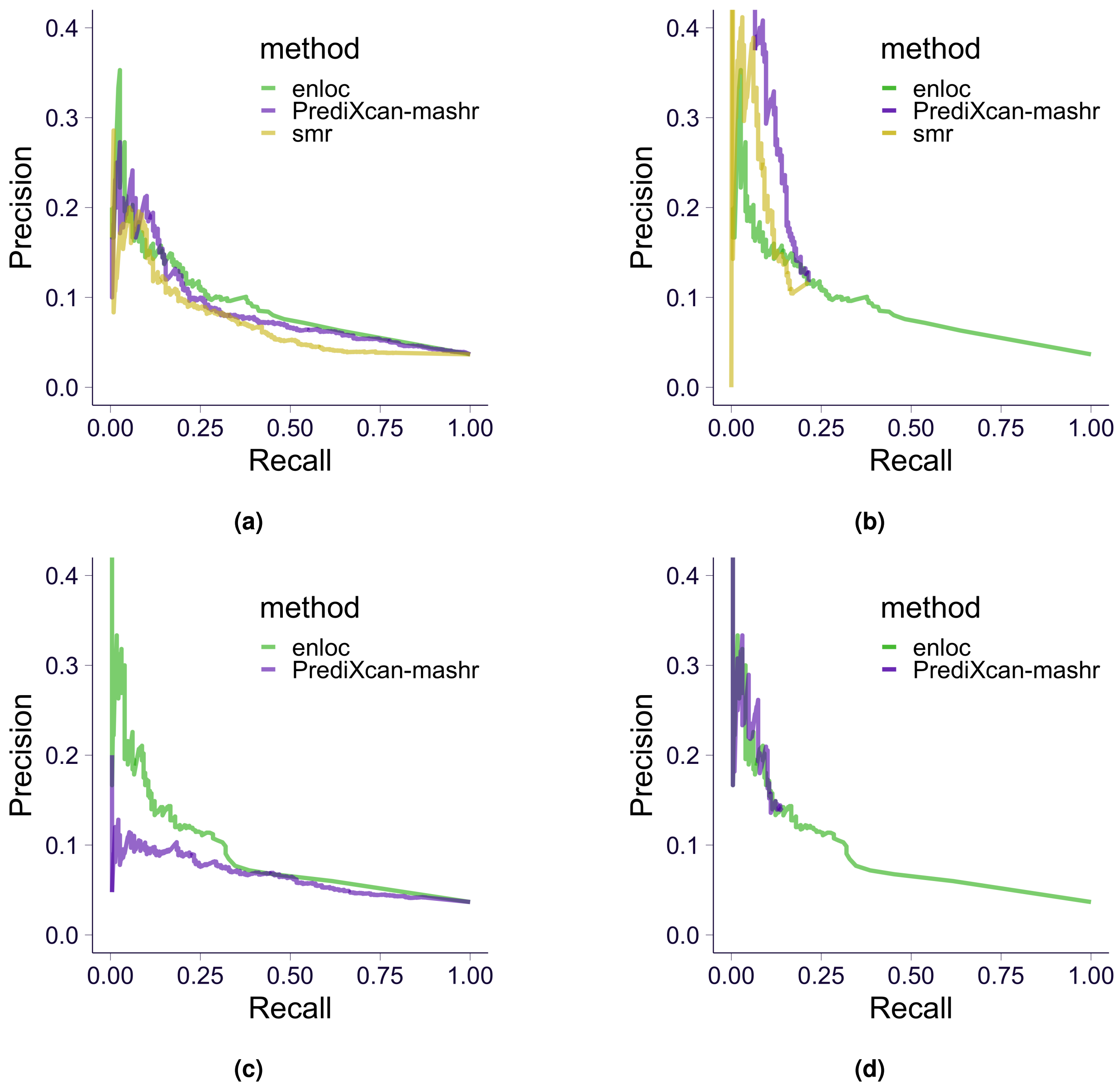

Additional Fig. A8: Precision-recall curves of colocalization/association based methods on OMIM silver standard. The results on expression data are shown in top row and the ones on splicing data are shown in bottom row. **(A,C)** Precision-recall curve of colocalization/association based methods. **(B,D)** Precision-recall curve of association based methods when pre-filtering with *enloc* rcp > 0.1.

ROC and PR curves under permuted data

To examine if the shapes of PR and ROC curves were driven by the bias buried in the data, we plotted the PR and ROC curves under the permuted data. Specifically, for each GWAS locus, we permuted the

genes that overlapped with the locus while keeping the scores unchanged. We compared the PR and ROC curves for observed and permuted data for OMIM silver standard as shown in Fig. S11 and Fig. A9.

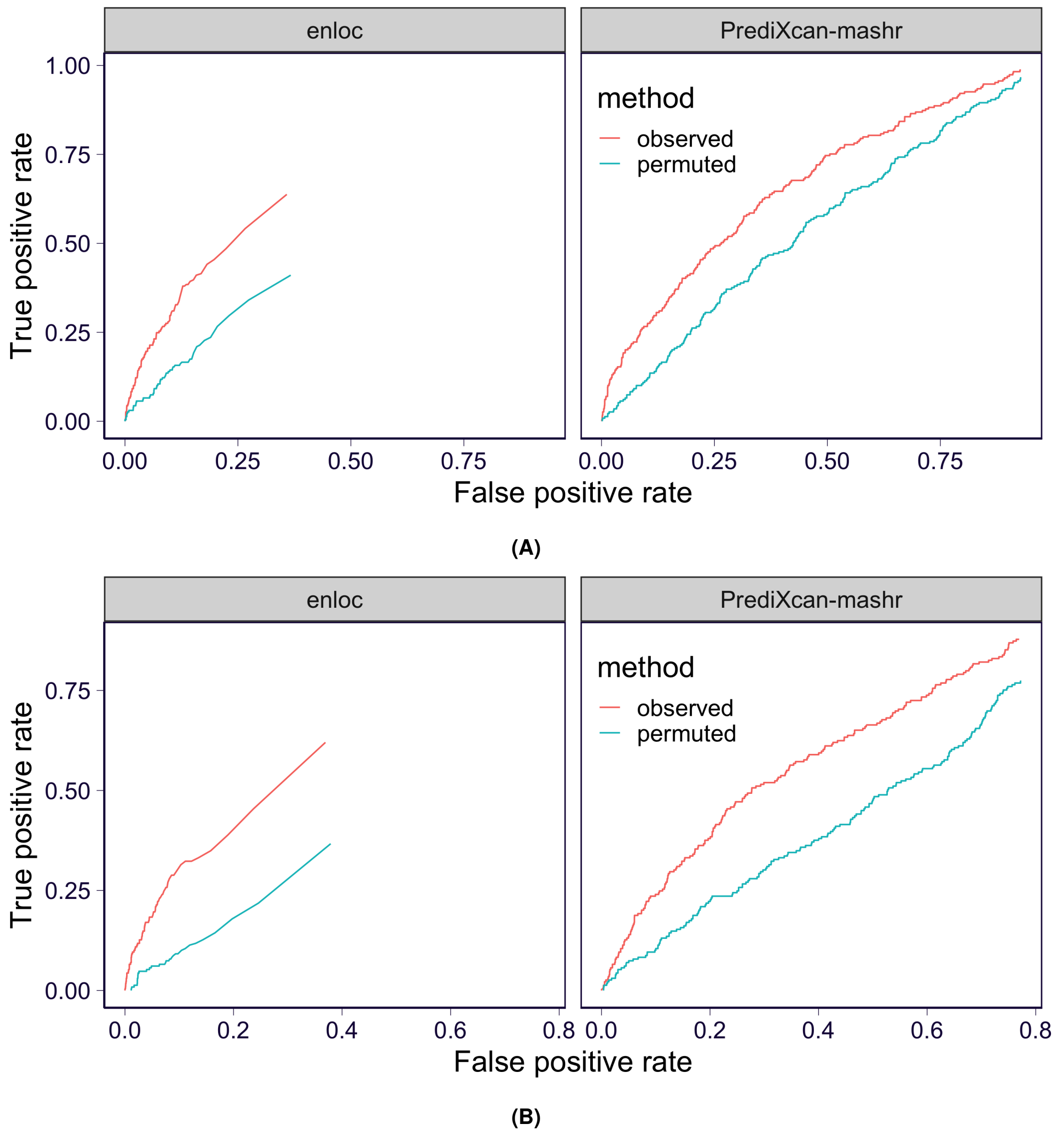

Fig S11. The ROC curves under permuted data based on OMIM silver standard. The ROC curves under observed and permuted data are shown in **(A)** (for expression based analysis) and **(B)** (for splicing based analysis).

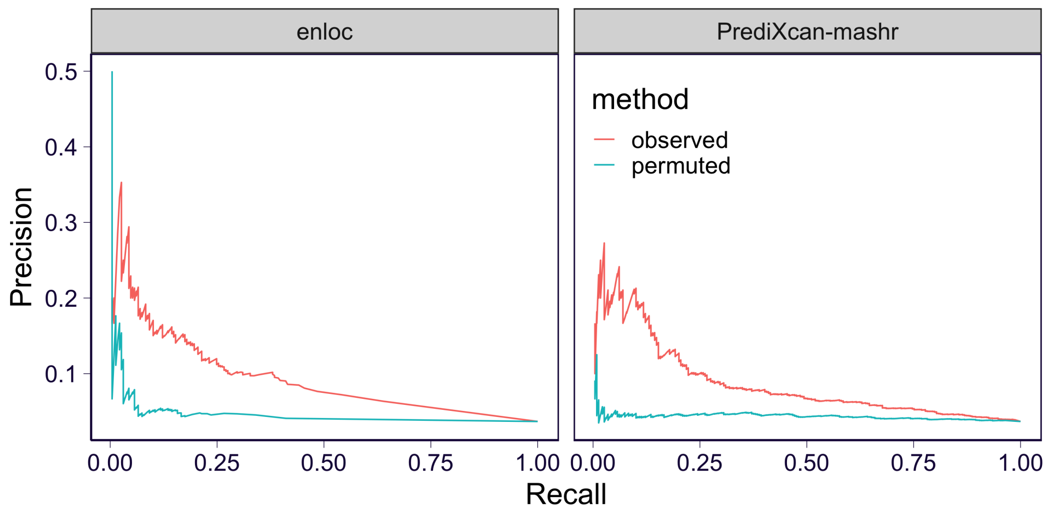

(a)

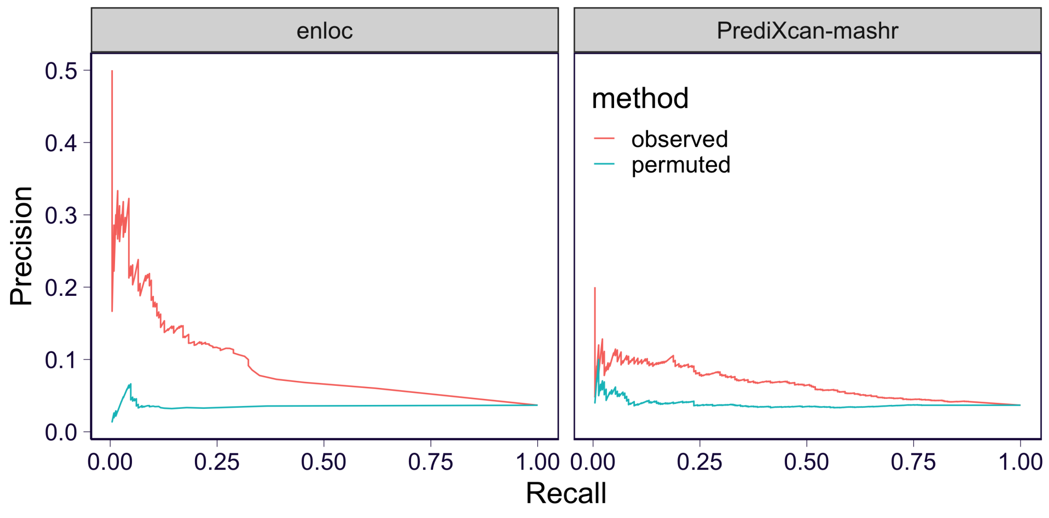

(b)

Additional Fig. A9: The PR curves under permuted data based on OMIM silver standard. The PR curves under observed and permuted data are shown in **(A)** (for expression based analysis) and **(B)** (for splicing based analysis).

13.6 Precision-recall curves of PrediXcan and *enloc* on the rare variant association-based silver standard gene set

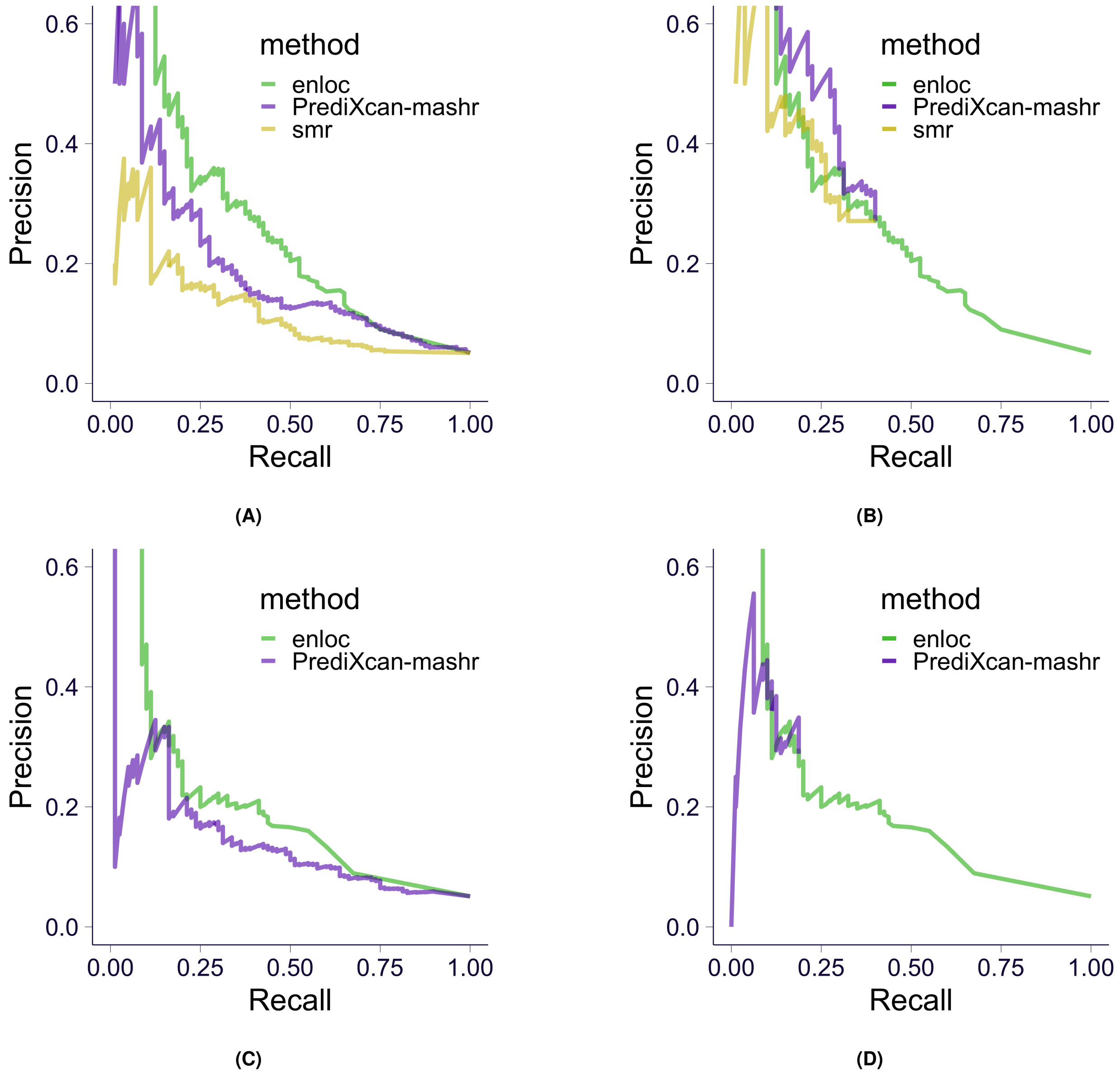

Fig S12. Precision-recall curves of colocalization/association based methods on rare variant-based silver standard. The results on expression data are shown in top row and the ones on splicing data are shown in bottom row. **(A,C)** Precision-recall curve of colocalization/association based methods. **(B,D)** Precision-recall curve of association based methods when pre-filtering with *enloc* rcp > 0.1.

13.7 Comparing precision-recall curves of *coloc* and *enloc*

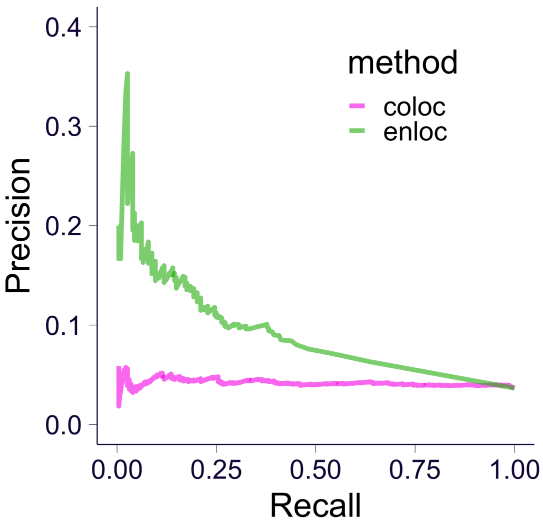

(a)

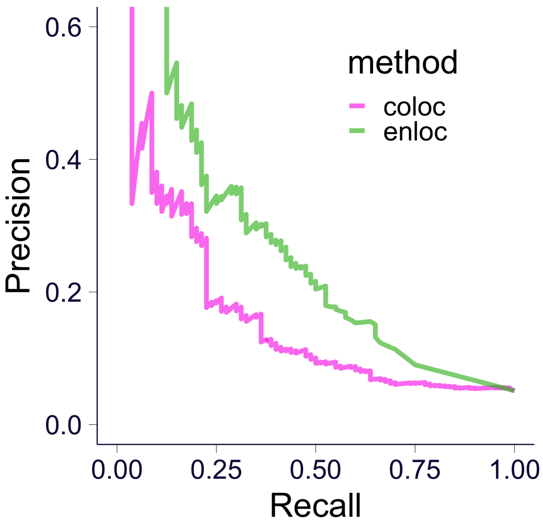

(b)

Additional Fig. A10: Precision-recall curves of *enloc* vs *coloc*. Precision recall curve of *enloc* (blue) and *coloc* (green) with expression using OMIM silver standard (in **(A)**) and rare variant-based silver standard (in **(B)**).

13.8 Assessing the contribution of proximity, colocalization, and association significance

To investigate the usefulness of the colocalization and association statistics reported by *enloc* and PrediXcan respectively, we performed logistic regression, as described in Eq. 28, to fit log odds of being a 'causal' gene against the ranking of: 1) proximity to GWAS lead variant (from close to distal), 2) rcp from *enloc* (from high to low), and 3) gene-level association p-value from PrediXcan-*mashr* or SMR (from significant to non-significant).

$$\text{logit}(\Pr(\text{causal}_i)) = \beta_0 + \beta_1 \cdot \text{rank}(\text{proximity}_i) + \beta_2 \cdot \text{rank}(\text{rcp}_i) + \beta_3 \cdot \text{rank}(\text{P-value}_i), \quad (28)$$

in which non-zero β_k meant that the kth variable contributed independently on predicting whether a gene was causal. Moreover, negative β_k indicated that the direction of contribution of the variable was as expected.

We note that here the analysis is performed by LD blocks rather than genome-wide as was done for calculating the ROC and precision recall curves. More specifically, the ranking within each LD block is used rather than genome-wide.

regulation	silver_standard	variable	coefficient	coefficient_se	pvalue
expression	OMIM	rank_proximity	-0.018	0.0081	0.03
expression	OMIM	predixcan_mashr_eur	-0.038	0.008	2.2×10^{-6}
expression	OMIM	enloc	-0.02	0.0093	0.031
splicing	OMIM	rank_proximity	-0.026	0.0073	0.00031
splicing	OMIM	predixcan_mashr_eur	-0.037	0.008	3.5×10^{-6}
splicing	OMIM	enloc	-0.012	0.0086	0.17
expression	rare variant	rank_proximity	-0.013	0.018	0.46
expression	rare variant	predixcan_mashr_eur	-0.043	0.016	0.0084
expression	rare variant	enloc	-0.043	0.02	0.032
splicing	rare variant	rank_proximity	-0.048	0.015	0.0015
splicing	rare variant	predixcan_mashr_eur	-0.018	0.013	0.15
splicing	rare variant	enloc	-0.02	0.015	0.2

Table S6: Predictive value of different per-locus prioritization methods. Results on regression-based test (logistic regression) in per-locus analysis are shown. The estimated log odds ratio of the rank of proximity (distance between GWAS leading variant and gene body), PrediXcan significance, and *enloc* rcp are shown in rows **rank_proximity**, **predixcan_mashr_eur**, and **enloc**.

14 Causal tissue analysis

To identify tissues of relevance for the etiology of complex traits, we investigated the patterns of tissue specificity and tissue sharing of PrediXcan association results across 49 tissues. For each trait-gene pair, the PrediXcan z-score can be represented as a 49×1 vector with each entry being the gene-level z-score in the corresponding tissue (if the prediction model of the gene is not available in that tissue, we filled in zero). To explore the tissue-specificity of the PrediXcan z-score vector, we proceeded by assigning the z-score vector to a tissue-pattern category and tested whether certain tissue-pattern categories were over-represented among colocalized PrediXcan genes as compared to non-colocalized genes. We used the FLASH factors identified from matrix factorization applied to the cis-eQTL effect size matrix, as described in Section 9 (as PrediXcan and cis-eQTL shared similar tissue-sharing pattern, data not shown). To obtain a set of detailed and biologically interpretable tissue-pattern categories from the 31 FLASH factors, we manually merged them into 18 categories as shown in Fig. S13. For each trait, we projected the z-score vector of each gene to one of the 31 FLASH factors (as described in Section 9) so that the gene was assigned to the corresponding tissue-pattern category. We defined a ‘positive’ set of genes as the ones with PrediXcan p-value that meets Bonferroni significance at $\alpha = 0.05$ in at least one tissue and *enloc* rcp > 0.01 in at least one tissue, which could be thought as a set of candidate genes affecting the trait through expression level. We chose a rather low threshold used for the rcp due to the stringent conservative nature of colocalization probabilities. We also constructed a ‘negative’ set of genes with *enloc* rcp = 0, which could be thought as a set of genes whose expressions were unlikely to affect the trait. We proceeded to test whether certain tissue-pattern categories were enriched in ‘positive’ set as compared to ‘negative’ set. Since the main focus of this analysis was tissue-specific patterns, we excluded *Factor1* (the cross-tissue factor) and *Factor25* (likely to be a tissue-shared factor capturing tissues with large sample size). Additionally, we excluded *Factor7* (testis), as it was unlikely to be the mediating tissue but might introduce false positives. We tested the enrichment of each tissue-pattern category by Fisher’s exact test (‘positive’/‘negative’ sets and in/not in tissue-patter category). Among 87 traits, 82 traits had *enloc* signal and the enrichment of these was calculated accordingly.

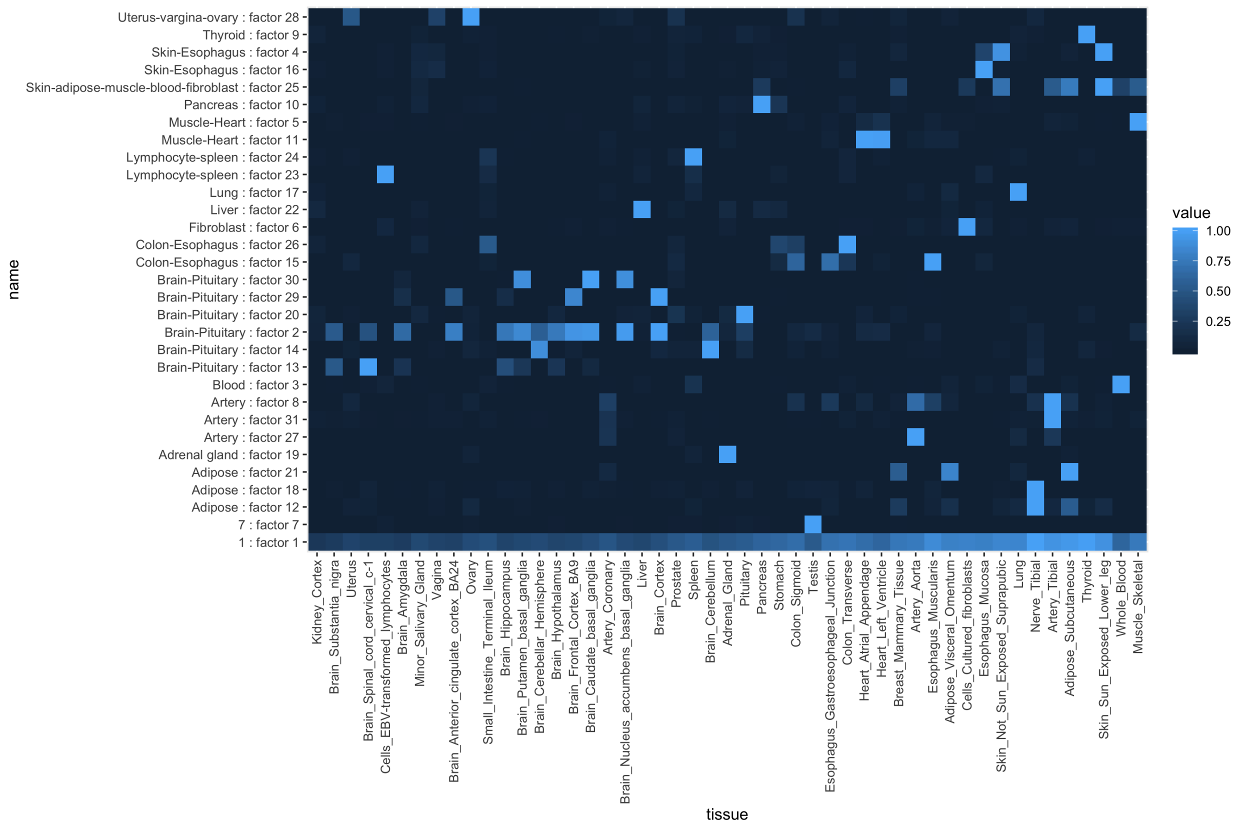

Fig S13. Patterns of tissue sharing identified via factor analysis using flashr. Tissue-pattern categories generated from FLASH applied to the cis-eQTLs are shown. Factor 1 represents cross tissue category covering all tissues, with higher weight for larger sample size tissues. These tissue categories (on y-axis) were used in the analysis of causal tissue identification. Tissues are ordered by sample size.

References

V. Anttila, B. S. Winsvold, P. Gormley, T. Kurth, F. Bettella, G. McMahon, M. Kallela, R. Malik, B. de Vries, G. Terwindt, S. E. Medland, U. Todt, W. L. McArdle, L. Quaye, M. Koiranen, M. A. Ikram, T. Lehtimaki, A. H. Stam, L. Ligthart, J. Wedenoja, I. Dunham, B. M. Neale, P. Palta, E. Hamalainen, M. Schurks, L. M. Rose, J. E. Buring, P. M. Ridker, S. Steinberg, H. Stefansson, F. Jakobsson, D. A. Lawlor, D. M. Evans, S. M. Ring, M. Farkkila, V. Artto, M. A. Kaunisto, T. Freilinger, J. Schoenen, R. R. Frants, N. Pelzer, C. M. Weller, R. Zielman, A. C. Heath, P. A. F. Madden, G. W. Montgomery, N. G. Martin, G. Borck, H. Gobel, A. Heinze, K. Heinze-Kuhn, F. M. K. Williams, A.-L. Hartikainen, A. Pouta, J. van den Ende, A. G. Uitterlinden, A. Hofman, N. Amin, J.-J. Hottenga, J. M. Vink, K. Heikkila, M. Alexander, B. Muller-Myhsok, S. Schreiber, T. Meitinger, H. E. Wichmann, A. Aromaa, J. G. Eriksson, B. Traynor, D. Trabzuni, E. Rossin, K. Lage, S. B. R. Jacobs, J. R. Gibbs, E. Birney, J. Kaprio, B. W. Penninx, D. I. Boomsma, C. van Duijn, O. Raitakari, M.-R. Jarvelin, J.-A. Zwart, L. Cherkas, D. P. Strachan, C. Kubisch, M. D. Ferrari, A. M. J. M. van den Maagdenberg, M. Dichgans, M. Wessman, G. D. Smith, K. Stefansson, M. J. Daly, D. R. Nyholt, D. Chasman, and A. Palotie. Genome-wide meta-analysis identifies new susceptibility loci for migraine. *Nature genetics*, 45(8):912–917, Aug. 2013. ISSN 1546-1718 1061-4036. doi: 10.1038/ng.2676.

A. N. Barbeira, S. P. Dickinson, R. Bonazzola, J. Zheng, H. E. Wheeler, J. M. Torres, E. S. Torstenson, K. P. Shah, T. Garcia, T. L. Edwards, E. A. Stahl, L. M. Huckins, F. Aguet, K. G. Ardlie, B. B. Cummings, E. T. Gelfand, G. Getz, K. Hadley, R. E. Handsaker, K. H. Huang, S. Kashin, K. J. Karczewski, M. Lek, X. Li, D. G. MacArthur, J. L. Nedzel, D. T. Nguyen, M. S. Noble, A. V. Segrè, C. A. Trowbridge, T. Tukiainen, N. S. Abell, B. Balliu, R. Barshir, O. Basha, A. Battle, G. K. Bogu, A. Brown, C. D. Brown, S. E. Castel, L. S. Chen, C. Chiang, D. F. Conrad, F. N. Damani, J. R. Davis, O. Delaneau, E. T. Dermitzakis, B. E. Engelhardt, E. Eskin, P. G. Ferreira, L. Frésard, E. R. Gamazon, D. Garrido-Martín, A. D. Gewirtz, G. Gliner, M. J. Gloudemans, R. Guigo, I. M. Hall, B. Han, Y. He, F. Hormozdiari, C. Howald, B. Jo, E. Y. Kang, Y. Kim, S. Kim-Hellmuth, T. Lappalainen, G. Li, X. Li, B. Liu, S. Mangul, M. I. McCarthy, I. C. McDowell, P. Mohammadi, J. Monlong, S. B. Montgomery, M. Muñoz-Aguirre, A. W. Ndungu, A. B. Nobel, M. Oliva, H. Ongen, J. J. Palowitch, N. Panousis, P. Papasaikas, Y. S. Park, P. Parsana, A. J. Payne, C. B. Peterson, J. Quan, F. Reverter, C. Sabatti, A. Saha, M. Sammeth, A. J. Scott, A. A. Shabalin, R. Sodaei, M. Stephens, B. E. Stranger, B. J. Strober, J. H. Sul, E. K. Tsang, S. Urbut, M. Van De Bunt, G. Wang, X. Wen, F. A. Wright, H. S. Xi, E. Yeger-Lotem, Z. Zappala, J. B. Zaugg, Y. H. Zhou, J. M. Akey, D. Bates, J. Chan, M. Claussnitzer, K. Demanelis, M. Diegel, J. A. Doherty, A. P. Feinberg, M. S. Fernando, J. Halow, K. D. Hansen, E. Haugen, P. F. Hickey, L. Hou, F. Jasmine, R. Jian, L. Jiang, A. Johnson, R. Kaul, M. Kellis, M. G. Kibriya, K. Lee, J. B. Li, Q. Li, J. Lin, S. Lin, S. Linder, C. Linke, Y. Liu, M. T. Maurano, B. Molinie, J. Nelson, F. J. Neri, Y. Park, B. L. Pierce, N. J. Rinaldi, L. F. Rizzardi, R. Sandstrom, A. Skol, K. S. Smith, M. P. Snyder, J. Stamatoyannopoulos, H. Tang, L. Wang, M. Wang, N. Van Wittenberghe, F. Wu, R. Zhang, C. R. Nierras, P. A. Branton, L. J. Carithers, P. Guan, H. M. Moore, A. Rao, J. B. Vaught, S. E. Gould, N. C. Lockart, C. Martin, J. P. Struewing, S. Volpi, A. M. Addington, S. E. Koester, A. R. Little, L. E. Brigham, R. Hasz, M. Hunter, C. Johns, M. Johnson, G. Kopen, W. F. Leinweber, J. T. Lonsdale, A. McDonald, B. Mestichelli, K. Myer, B. Roe, M. Salvatore, S. Shad, J. A. Thomas, G. Walters, M. Washington, J. Wheeler, J. Bridge, B. A. Foster, B. M. Gillard, E. Karasik, R. Kumar, M. Miklos, M. T. Moser, S. D. Jewell, R. G. Montroy, D. C. Rohrer, D. R. Valley, D. A. Davis, D. C. Mash, A. H. Undale, A. M. Smith, D. E. Tabor, N. V. Roche, J. A. McLean, N. Vatanian, K. L. Robinson, L. Sobin, M. E. Barcus, K. M. Valentino, L. Qi, S. Hunter, P. Hariharan, S. Singh, K. S. Um, T. Matose, M. M. Tomaszewski, L. K. Barker, M. Mosavel, L. A. Siminoff, H. M. Traino, P. Flicek, T. Juettemann, M. Ruffier, D. Sheppard, K. Taylor, S. J. Trevanion, D. R. Zerbino, B. Craft, M. Goldman, M. Haeussler, W. J. Kent, C. M. Lee, B. Paten, K. R. Rosenbloom, J. Vivian, J. Zhu, D. L. Nicolae, N. J. Cox, and H. K. Im. Exploring the phenotypic consequences of tissue specific gene expression variation inferred from GWAS summary statistics. *Nature Communications*, 2018. ISSN 20411723. doi: 10.1038/s41467-018-03621-1.

A. N. Barbeira, M. Pividori, J. Zheng, H. E. Wheeler, D. L. Nicolae, and H. K. Im. Integrating predicted

transcriptome from multiple tissues improves association detection. *PLOS Genetics*, 15(1):1–20, 01 2019. doi: 10.1371/journal.pgen.1007889. URL https://doi.org/10.1371/journal.pgen.1007889.

A. N. Barbeira, O. J. Melia, Y. Liang, R. Bonazzola, G. Wang, H. E. Wheeler, F. Aguet, K. G. Ardlie, X. Wen, and H. K. Im. Fine-mapping and qtl tissue-sharing information improves the reliability of causal gene identification. *Genetic Epidemiology*, n/a(n/a), 2020. doi: 10.1002/gepi.22346. URL https://onlinelibrary.wiley.com/doi/abs/10.1002/gepi.22346.

L. Bastarache, J. J. Hughey, S. Hebbring, J. Marlo, W. Zhao, W. T. Ho, S. L. Van Driest, T. L. McGregor, J. D. Mosley, Q. S. Wells, et al. Phenotype risk scores identify patients with unrecognized mendelian disease patterns. *Science*, 359(6381):1233–1239, 2018.

T. Berisa and J. K. Pickrell. Approximately independent linkage disequilibrium blocks in human populations. *Bioinformatics*, 32(2):283–285, Jan. 2016. ISSN 1367-4803 1367-4811. doi: 10.1093/bioinformatics/btv546.

J. Bowden, G. Davey Smith, and S. Burgess. Mendelian randomization with invalid instruments: effect estimation and bias detection through Egger regression. *International Journal of Epidemiology*, 44(2): 512–525, June 2015. doi: 10.1093/ije/dyv080. URL http://www.ije.oxfordjournals.org/cgi/doi/10.1093/ije/dyv080.

A. Buniello, J. A. Macarthur, M. Cerezo, L. W. Harris, J. Hayhurst, C. Malangone, A. McMahon, J. Morales, E. Mountjoy, E. Sollis, D. Suveges, O. Vrousgou, P. L. Whetzel, R. Amode, J. A. Guillen, H. S. Riat, S. J. Trevanion, P. Hall, H. Junkins, P. Flicek, T. Burdett, L. A. Hindorff, F. Cunningham, and H. Parkinson. The NHGRI-EBI GWAS Catalog of published genome-wide association studies, targeted arrays and summary statistics 2019. *Nucleic Acids Research*, 2019. ISSN 13624962. doi: 10.1093/nar/gky1120.

C. Bycroft, C. Freeman, D. Petkova, G. Band, L. T. Elliott, K. Sharp, A. Motyer, D. Vukcevic, O. Delaneau, J. O'Connell, A. Cortes, S. Welsh, A. Young, M. Effingham, G. McVean, S. Leslie, N. Allen, P. Donnelly, and J. Marchini. The UK Biobank resource with deep phenotyping and genomic data. *Nature*, 562(7726): 203–209, 2018. ISSN 1476-4687. doi: 10.1038/s41586-018-0579-z. URL https://doi.org/10.1038/s41586-018-0579-z.

D. S. DeLuca, J. Z. Levin, A. Sivachenko, T. Fennell, M.-D. Nazaire, C. Williams, M. Reich, W. Winckler, and G. Getz. RNA-SeQC: RNA-seq metrics for quality control and process optimization. *Bioinformatics*, 28 (11):1530–1532, June 2012.

J. C. Denny, L. Bastarache, M. D. Ritchie, R. J. Carroll, R. Zink, J. D. Mosley, J. R. Field, J. M. Pulley, A. H. Ramirez, E. Bowton, M. A. Basford, D. S. Carrell, P. L. Peissig, A. N. Kho, J. A. Pacheco, L. V. Rasmussen, D. R. Crosslin, P. K. Crane, J. Pathak, S. J. Bielinski, S. A. Pendergrass, H. Xu, L. A. Hindorff, R. Li, T. A. Manolio, C. G. Chute, R. L. Chisholm, E. B. Larson, G. P. Jarvik, M. H. Brilliant, C. A. Mccarty, I. J. Kullo, J. L. Haines, D. C. Crawford, D. R. Masys, and D. M. Roden. Systematic comparison of phenome-wide association study of electronic medical record data and genome-wide association study data. *Nature Biotechnology*, 31(12):1102, 2013. ISSN 10870156. doi: 10.1038/nbt.2749.

A. Dobin, C. A. Davis, F. Schlesinger, J. Drenkow, C. Zaleski, S. Jha, P. Batut, M. Chaisson, and T. R. Gingeras. STAR: ultrafast universal RNA-seq aligner. *Bioinformatics*, 29(1):15–21, Jan. 2013. ISSN 1367-4803. doi: 10.1093/bioinformatics/bts635. URL http://dx.doi.org/10.1093/bioinformatics/bts635.

C. Giambartolomei, D. Vukcevic, E. E. Schadt, L. Franke, A. D. Hingorani, C. Wallace, and V. Plagnol. Bayesian Test for Colocalisation between Pairs of Genetic Association Studies Using Summary Statistics. *PLOS Genetics*, 10(5):1–15, 2014. doi: 10.1371/journal.pgen.1004383. URL https://doi.org/10.1371/journal.pgen.1004383.

GTEx Consortium, F. Aguet, A. A. Brown, S. E. Castel, J. R. Davis, Y. He, B. Jo, P. Mohammadi, Y. Park, P. Parsana, A. V. Segrè, B. J. Strober, Z. Zappala, B. B. Cummings, E. T. Gelfand, K. Hadley, K. H. Huang, M. Lek, X. Li, J. L. Nedzel, D. Y. Nguyen, M. S. Noble, T. J. Sullivan, T. Tukiainen, D. G. MacArthur, G. Getz, A. Addington, P. Guan, S. Koester, A. R. Little, N. C. Lockhart, H. M. Moore, A. Rao, J. P. Struewing, S. Volpi, L. E. Brigham, R. Hasz, M. Hunter, C. Johns, M. Johnson, G. Kopen, W. F. Leinweber, J. T. Lonsdale, A. McDonald, B. Mestichelli, K. Myer, B. Roe, M. Salvatore, S. Shad, J. A. Thomas, G. Walters, M. Washington, J. Wheeler, J. Bridge, B. A. Foster, B. M. Gillard, E. Karasik, R. Kumar, M. Miklos, M. T. Moser, S. D. Jewell, R. G. Montroy, D. C. Rohrer, D. Valley, D. C. Mash, D. A. Davis, L. Sobin, M. E. Barcus, P. A. Branton, N. S. Abell, B. Balliu, O. Delaneau, L. Frésard, E. R. Gamazon, D. Garrido-Martín, A. D. H. Gewirtz, G. Gliner, M. J. Gloudemans, B. Han, A. Z. He, F. Hormozdiari, X. Li, B. Liu, E. Y. Kang, I. C. McDowell, H. Ongen, J. J. Palowitch, C. B. Peterson, G. Quon, S. Ripke, A. Saha, A. A. Shabalin, T. C. Shimko, J. H. Sul, N. A. Teran, E. K. Tsang, H. Zhang, Y.-H. Zhou, C. D. Bustamante, N. J. Cox, R. Guigó, M. Kellis, M. I. McCarthy, D. F. Conrad, E. Eskin, G. Li, A. B. Nobel, C. Sabatti, B. E. Stranger, X. Wen, F. A. Wright, K. G. Ardlie, E. T. Dermitzakis, T. Lappalainen, F. Aguet, K. G. Ardlie, B. B. Cummings, E. T. Gelfand, G. Getz, K. Hadley, R. E. Handsaker, K. H. Huang, S. Kashin, K. J. Karczewski, M. Lek, X. Li, D. G. MacArthur, J. L. Nedzel, D. T. Nguyen, M. S. Noble, A. V. Segrè, C. A. Trowbridge, T. Tukiainen, N. S. Abell, B. Balliu, R. Barshir, O. Basha, A. Battle, G. K. Bogu, A. Brown, C. D. Brown, S. E. Castel, L. S. Chen, C. Chiang, D. F. Conrad, N. J. Cox, F. N. Damani, J. R. Davis, O. Delaneau, E. T. Dermitzakis, B. E. Engelhardt, E. Eskin, P. G. Ferreira, L. Frésard, E. R. Gamazon, D. Garrido-Martín, A. D. Gewirtz, G. Gliner, M. J. Gloudemans, R. Guigo, I. M. Hall, B. Han, Y. He, F. Hormozdiari, C. Howald, H. Kyung Im, B. Jo, E. Yong Kang, Y. Kim, S. Kim-Hellmuth, T. Lappalainen, G. Li, X. Li, B. Liu, S. Mangul, M. I. McCarthy, I. C. McDowell, P. Mohammadi, J. Monlong, S. B. Montgomery, M. Muñoz-Aguirre, A. W. Ndungu, D. L. Nicolae, A. B. Nobel, M. Oliva, H. Ongen, J. J. Palowitch, N. Panousis, P. Papasaikas, Y. Park, P. Parsana, A. J. Payne, C. B. Peterson, J. Quan, F. Reverter, C. Sabatti, A. Saha, M. Sammeth, A. J. Scott, A. A. Shabalin, R. Sodaei, M. Stephens, B. E. Stranger, B. J. Strober, J. H. Sul, E. K. Tsang, S. Urbut, M. van de Bunt, G. Wang, X. Wen, F. A. Wright, H. S. Xi, E. Yeger-Lotem, Z. Zappala, J. B. Zaugg, Y.-H. Zhou, J. M. Akey, D. Bates, J. Chan, L. S. Chen, M. Claussnitzer, K. Demanelis, M. Diegel, J. A. Doherty, A. P. Feinberg, M. S. Fernando, J. Halow, K. D. Hansen, E. Haugen, P. F. Hickey, L. Hou, F. Jasmine, R. Jian, L. Jiang, A. Johnson, R. Kaul, M. Kellis, M. G. Kibriya, K. Lee, J. Billy Li, Q. Li, X. Li, J. Lin, S. Lin, S. Linder, C. Linke, Y. Liu, M. T. Maurano, B. Molinie, S. B. Montgomery, J. Nelson, F. J. Neri, M. Oliva, Y. Park, B. L. Pierce, N. J. Rinaldi, L. F. Rizzardi, R. Sandstrom, A. Skol, K. S. Smith, M. P. Snyder, J. Stamatoyannopoulos, B. E. Stranger, H. Tang, E. K. Tsang, L. Wang, M. Wang, N. Van Wittenberghe, F. Wu, R. Zhang, C. R. Nierras, P. A. Branton, L. J. Carithers, P. Guan, H. M. Moore, A. Rao, J. B. Vaught, S. E. Gould, N. C. Lockart, C. Martin, J. P. Struewing, S. Volpi, A. M. Addington, S. E. Koester, A. R. Little, L. E. Brigham, R. Hasz, M. Hunter, C. Johns, M. Johnson, G. Kopen, W. F. Leinweber, J. T. Lonsdale, A. McDonald, B. Mestichelli, K. Myer, B. Roe, M. Salvatore, S. Shad, J. A. Thomas, G. Walters, M. Washington, J. Wheeler, J. Bridge, B. A. Foster, B. M. Gillard, E. Karasik, R. Kumar, M. Miklos, M. T. Moser, S. D. Jewell, R. G. Montroy, D. C. Rohrer, D. R. Valley, D. A. Davis, D. C. Mash, A. H. Undale, A. M. Smith, D. E. Tabor, N. V. Roche, J. A. McLean, N. Vatanian, K. L. Robinson, L. Sobin, M. E. Barcus, K. M. Valentino, L. Qi, S. Hunter, P. Hariharan, S. Singh, K. S. Um, T. Matose, M. M. Tomaszewski, L. K. Barker, M. Mosavel, L. A. Siminoff, H. M. Traino, P. Flicek, T. Juettemann, M. Ruffier, D. Sheppard, K. Taylor, S. J. Trevanion, D. R. Zerbino, B. Craft, M. Goldman, M. Haeussler, W. J. Kent, C. M. Lee, B. Paten, K. R. Rosenbloom, J. Vivian, and J. Zhu. Genetic effects on gene expression across human tissues. *Nature*, 550:204, Oct. 2017. URL http://dx.doi.org/10.1038/nature24277.

A. Hamosh, A. F. Scott, J. S. Amberger, C. A. Bocchini, and V. A. McKusick. Online Mendelian Inheritance in Man (OMIM), a knowledgebase of human genes and genetic disorders. *Nucleic Acids Research*, 2005. ISSN 03051048. doi: 10.1093/nar/gki033.

S. Köhler, S. C. Doelken, C. J. Mungall, S. Bauer, H. V. Firth, I. Bailleul-Forestier, G. C. Black, D. L. Brown, M. Brudno, J. Campbell, et al. The human phenotype ontology project: linking molecular biology and disease through phenotype data. *Nucleic acids research*, 42(D1):D966–D974, 2013.

D. Lee, T. B. Bigdeli, B. P. Riley, A. H. Fanous, and S. A. Bacanu. DIST: Direct imputation of summary statistics for unmeasured SNPs. *Bioinformatics*, 29(22):2925–2927, 2013. ISSN 13674803. doi: 10.1093/bioinformatics/btt500.

B. Li and C. N. Dewey. RSEM: accurate transcript quantification from RNA-Seq data with or without a reference genome. *BMC Bioinformatics*, 12(1):323, Aug. 2011. ISSN 1471-2105. doi: 10.1186/1471-2105-12-323. URL https://doi.org/10.1186/1471-2105-12-323.

Y. I. Li, D. A. Knowles, J. Humphrey, A. N. Barbeira, S. P. Dickinson, H. K. Im, and J. K. Pritchard. Annotation-free quantification of RNA splicing using LeafCutter. *Nature Genetics*, 50(1):151–158, Jan. 2018. ISSN 1546-1718. doi: 10.1038/s41588-017-0004-9. URL https://doi.org/10.1038/s41588-017-0004-9.

D. J. Liu, G. M. Peloso, H. Yu, A. S. Butterworth, X. Wang, A. Mahajan, D. Saleheen, C. Emdin, D. Alam, A. C. Alves, et al. Exome-wide association study of plasma lipids in> 300,000 individuals. *Nature genetics*, 49 (12):1758, 2017.

A. E. Locke, K. M. Steinberg, C. W. Chiang, S. K. Service, A. S. Havulinna, L. Stell, M. Pirinen, H. J. Abel, C. C. Chiang, R. S. Fulton, et al. Exome sequencing of finnish isolates enhances rare-variant association power. *Nature*, page 1, 2019.

J. Malone, E. Holloway, T. Adamusiak, M. Kapushesky, J. Zheng, N. Kolesnikov, A. Zhukova, A. Brazma, and H. Parkinson. Modeling sample variables with an Experimental Factor Ontology. *Bioinformatics*, 26 (8):1112–1118, 2010. ISSN 13674803. doi: 10.1093/bioinformatics/btq099.

E. Marouli, M. Graff, C. Medina-Gomez, K. S. Lo, A. R. Wood, T. R. Kjaer, R. S. Fine, Y. Lu, C. Schurmann, H. M. Highland, et al. Rare and low-frequency coding variants alter human adult height. *Nature*, 542 (7640):186, 2017.

K. Musunuru, A. Strong, M. Frank-Kamenetsky, N. E. Lee, T. Ahfeldt, K. V. Sachs, X. Li, H. Li, N. Kuperwasser, V. M. Ruda, J. P. Pirruccello, B. Muchmore, L. Prokunina-Olsson, J. L. Hall, E. E. Schadt, C. R. Morales, S. Lund-Katz, M. C. Phillips, J. Wong, W. Cantley, T. Racie, K. G. Ejebe, M. Orho-Melander, O. Melander, V. Koteliansky, K. Fitzgerald, R. M. Krauss, C. A. Cowan, S. Kathiresan, and D. J. Rader. From noncoding variant to phenotype via SORT1 at the 1p13 cholesterol locus. *Nature*, 466 (7307):714–719, Aug. 2010. ISSN 1476-4687 0028-0836. doi: 10.1038/nature09266.

M. Nikpay, A. Goel, H.-H. Won, L. M. Hall, C. Willenborg, S. Kanoni, D. Saleheen, T. Kyriakou, C. P. Nelson, J. C. Hopewell, T. R. Webb, L. Zeng, A. Dehghan, M. Alver, S. M. Armasu, K. Auro, A. Bjonnes, D. I. Chasman, S. Chen, I. Ford, N. Franceschini, C. Gieger, C. Grace, S. Gustafsson, J. Huang, S.-J. Hwang, Y. K. Kim, M. E. Kleber, K. W. Lau, X. Lu, Y. Lu, L.-P. Lyytikainen, E. Mihailov, A. C. Morrison, N. Pervjakova, L. Qu, L. M. Rose, E. Salfati, R. Saxena, M. Scholz, A. V. Smith, E. Tikkanen, A. Uitterlinden, X. Yang, W. Zhang, W. Zhao, M. de Andrade, P. S. de Vries, N. R. van Zuydam, S. S. Anand, L. Bertram, F. Beutner, G. Dedoussis, P. Frossard, D. Gauguier, A. H. Goodall, O. Gottesman, M. Haber, B.-G. Han, J. Huang, S. Jalilzadeh, T. Kessler, I. R. Konig, L. Lannfelt, W. Lieb, L. Lind, C. M. Lindgren, M.-L. Lokki, P. K. Magnusson, N. H. Mallick, N. Mehra, T. Meitinger, F.-U.-R. Memon, A. P. Morris, M. S. Nieminen, N. L. Pedersen, A. Peters, L. S. Rallidis, A. Rasheed, M. Samuel, S. H. Shah, J. Sinisalo, K. E. Stirrups, S. Trompet, L. Wang, K. S. Zaman, D. Ardissino, E. Boerwinkle, I. B. Borecki, E. P. Bottinger, J. E. Buring, J. C. Chambers, R. Collins, L. A. Cupples, J. Danesh, I. Demuth, R. Elosua, S. E. Epstein, T. Esko, M. F. Feitosa, O. H. Franco, M. G. Franzosi, C. B. Granger, D. Gu, V. Gudnason, A. S. Hall, A. Hamsten, T. B. Harris, S. L. Hazen, C. Hengstenberg, A. Hofman, E. Ingelsson, C. Iribarren, J. W. Jukema, P. J. Karhunen, B.-J. Kim, J. S. Kooner, I. J. Kullo, T. Lehtimaki, R. J. F. Loos, O. Melander, A. Metspalu, W. Marz, C. N. Palmer, M. Perola, T. Quertermous, D. J. Rader, P. M. Ridker, S. Ripatti, R. Roberts, V. Salomaa, D. K. Sanghera, S. M. Schwartz, U. Seedorf, A. F. Stewart, D. J. Stott, J. Thiery, P. A. Zalloua, C. J. O'Donnell, M. P. Reilly, T. L. Assimes, J. R.

Thompson, J. Erdmann, R. Clarke, H. Watkins, S. Kathiresan, R. McPherson, P. Deloukas, H. Schunkert, N. J. Samani, and M. Farrall. A comprehensive 1,000 Genomes-based genome-wide association meta-analysis of coronary artery disease. *Nature genetics*, 47(10):1121–1130, Oct. 2015. ISSN 1546-1718 1061-4036. doi: 10.1038/ng.3396.

H. Ongen, A. Buil, A. A. Brown, E. T. Dermitzakis, and O. Delaneau. Fast and efficient QTL mapper for thousands of molecular phenotypes. *Bioinformatics*, 2016. ISSN 14602059. doi: 10.1093/bioinformatics/btv722.

B. Pasaniuc, N. Zaitlen, H. Shi, G. Bhatia, A. Gusev, J. Pickrell, J. Hirschhorn, D. P. Strachan, N. Patterson, and A. L. Price. Fast and accurate imputation of summary statistics enhances evidence of functional enrichment. *Bioinformatics*, 30(20):2906–2914, 2014. doi: 10.1093/bioinformatics/btu416. URL http://dx.doi.org/10.1093/bioinformatics/btu416.

A. L. Price, N. J. Patterson, R. M. Plenge, M. E. Weinblatt, N. A. Shadick, and D. Reich. Principal components analysis corrects for stratification in genome-wide association studies. *Nature Genetics*, 38:904, July 2006. URL http://dx.doi.org/10.1038/ng1847.

M. D. Robinson and A. Oshlack. A scaling normalization method for differential expression analysis of RNA-seq data. *Genome Biology*, 11(3):R25, Mar. 2010. ISSN 1474-760X. doi: 10.1186/gb-2010-11-3-r25. URL https://doi.org/10.1186/gb-2010-11-3-r25.

The GTEx Consortium. The gtex consortium atlas of genetic regulatory effects across human tissues. *Science*, 369(6509):1318–1330, 2020. ISSN 0036-8075. doi: 10.1126/science.aaz1776. URL https://science.sciencemag.org/content/369/6509/1318.

S. M. Urbut, G. Wang, P. Carbonetto, and M. Stephens. Flexible statistical methods for estimating and testing effects in genomic studies with multiple conditions. Technical report, Nature Publishing Group, 2018.

G. Wang, A. K. Sarkar, P. Carbonetto, and M. Stephens. A simple new approach to variable selection in regression, with application to genetic fine-mapping. *bioRxiv*, page 501114, 2018.

W. Wang and M. Stephens. Empirical bayes matrix factorization. *arXiv preprint arXiv:1802.06931*, 2018.

X. Wen. Molecular QTL discovery incorporating genomic annotations using Bayesian false discovery rate control. *Ann. Appl. Stat.*, 10(3):1619–1638, Sept. 2016. ISSN 1932-6157. doi: 10.1214/16-AOAS952. URL https://projecteuclid.org:443/euclid.aoas/1475069621.

X. Wen, R. Pique-Regi, and F. Luca. Integrating molecular QTL data into genome-wide genetic association analysis: Probabilistic assessment of enrichment and colocalization. *PLoS Genetics*, 13(3):e1006646, Mar. 2017. doi: 10.1371/journal.pgen.1006646. URL http://dx.plos.org/10.1371/journal.pgen.1006646.

Z. Zhu, F. Zhang, H. Hu, A. Bakshi, M. R. Robinson, J. E. Powell, G. W. Montgomery, M. E. Goddard, N. R. Wray, P. M. Visscher, and J. Yang. Integration of summary data from GWAS and eQTL studies predicts complex trait gene targets. *Nature genetics*, 48(5):481–487, May 2016. ISSN 1546-1718 1061-4036. doi: 10.1038/ng.3538.
