# Supplementary material for: Exploiting the GTEx resources to decipher the mechanisms at GWAS loci
Source: Genome Biol. 2021 Jan 26;22:49. doi: 10.1186/s13059-020-02252-4 (PMC7836161; doi:10.1186/s13059-020-02252-4)
Supplement: Supplementary file 10 — Additional file 10 Review history [file 13059_2020_2252_MOESM10_ESM.docx]

Review History

**First round of review**

**Reviewer 1**

**Are you able to assess all statistics in the manuscript, including the appropriateness of statistical tests used?**

Yes, and I have assessed the statistics in my report.

**Comments to author:**

The authors presented the results combining GWAS and GTEx resources to identify plausible mechanisms underlying GWAS signals, demonstrated dose-dependent effects of RNA expression and splicing, and suggested combing multiple approaches to prioritize causal genes. The manuscript is of broad interest to readers in the field, and the manuscript is clearly written. While the analyses are carefully executed, I have several concerns on the results.

Major comments:

1. The authors did a lot of excellent analyses, however, most results in the section of "causal gene prediction and prioritization" has been reported by GTEx consortium [Aguet et al., 2019]. And similar results in the section of "Performance for identifying ground truth genes" has also been reported by Barbeira et al. 2020 BioRxiv. More justification of the novelty of this manuscript is required.

2. The authors reported that 50% of GWAS signals lead to a target gene through eQTL or sQTL. This is an important claim. And it sounds a lot better than 11% of heritability being mediated through cis-eQTLs (PMID: 32424349), although we can't compare the two metrics directly. I would like to see some discussion between them. In addition, it is not clear how meaningful this percentage is, because this analyses don't explicitly demonstrate what would be expected by chance given the polygenicity of the traits and the high-dense coverage of eQTLs/sQTLs across the genome.

3. The authors used "ground truth" genes to compare the ability of different approaches to identify the causal gene that mediates the association between GWAS loci and the traits. The authors compared enloc, PrediXcan, and SMR. However, PrediXcan cannot distinguish between pleiotropy and linkage. It would be useful and informative to compare such methods which can distinguish pleiotropy from linkage (e.g., compare enloc and SMR+HEIDI). In addition, it would be useful to show the ROC curve by selecting a random set of genes that are included in the GWAS LD blocks.

Minor comments

4. Justification of the rcp threshold of 0.5 is required.

5. For the low colocalization of height signals between GIANT and UKB, the authors aimed to demonstrate that enloc is conservative, although the authors also acknowledged that uncorrected population structure in GWAS meta-analyses may also partly contribute to this. However, the data could not fully support this hypothesis. In other words, we can also interpret the results like this "population structure in GIANT data may be the major confounder of low colocalization.".

6. It is not clear how to derive the ROC curve if one GWAS locus contains multiple highly corelated genes.

7. While the section of "tissue enrichment of GWAS signals" appears to be sound, it lacks of the detail of the method, making it difficult to follow, although they can be found in Supplementary Text. More details of method are required in the main text to improve the readability.

8. On page 3 line 35, "Here we develop novel methods, approach, and resources". This is a strong statement given that all the data have been made publicly available by GTEx consortium and that all the methods used in this manuscript are developed by previous studies.

9. A typo in Figure S5. The circle represents all variants and square represents GWAS catalogue variant.

**Reviewer 2**

**Are you able to assess all statistics in the manuscript, including the appropriateness of statistical tests used?**

Yes, and I have assessed the statistics in my report.

**Comments to author:**

Barbeira, Bonazzola, Gamazon, Liand, Park and collaborators have carried out a thorough evaluation of the impact of the genetics of gene expression on complex traits and diseases. Using data from GTEx v8, they first uncover three pieces of evidence that imply a gene-dosage relationship between expression and phenotypes. Second, they perform a comprehensive query for causal genes at GWAS loci using colocalization and association with predicted gene expression, including replication in the BioVU cohort (for a number of traits available). Besides proposing candidate genes for many GWAS loci, these analyses provide many useful insights about the practices, features and methodologies that work best in regards to these approaches. Yet, a large fraction of loci associated with complex traits remains unresolved, and the low overlap in candidates observed for two large GWAS for height put an upper-bound for the potential of these methodologies to settle this aspect (here the authors conclude that further characterization of the genetic basis of gene expression is necessary). Evaluation in two independent sets of likely causal genes (elegantly ascertained 'silver genes') confirms these propositions. Third, and finally, they demonstrate that the catalogues of QTLs detected by GTEx provide insights about the tissue nature underlying complex traits and diseases.

This is an excellent study. The authors accomplish what I think is the most comprehensive examination of the many ways in which knowing about the genetic architecture of gene expression across tissues is useful to learn about the genetic basis of complex traits and disease susceptibility. Almost as important, the authors also provide a wealth of results across GTEx tissues and GWAS traits for several of the most used methods in statistical genetics, including coloc, dap-g fine-mapping, prediXcan, etc. Benchmarking them, making available the models to run new TWAS, providing a large catalogue of candidate genes, etc, all make of this work an excellent resource for the community. This is obviously possible thanks to the work of many different analysts, but I feel compelled to congratulate all the authors for putting this out.

The methods and analyses seem totally correct to me. The manuscript is well written and easy to follow (even if at times the main text is a bit lacking in methodological details, everything is covered with great detail in the supplementary materials). Rather than important flaws to be addressed, I have compiled a list of general comments and questions for the authors:

-	The observation that sets the scene in the main text (eQTL and sQTLs have a strong enrichment for good GWAS pvalues) refers to FigS5. The numbers in the figure are impressive. This is an important problem, since many researchers use blindly the evidence from QTL association to blindly infer causal genes. I would suggest discussing one or two examples of this enrichment in the main text (bottom in page5) so that readers can understand better the need for this manuscript

-	The concordance of effects in the allelic series analysis is a beautiful result. Why did the authors restrict to situations with two independent eQTLs? Is it due to lack of genes with >2 fine-mapped variants in GTEx (sample size)? Authors could use here known lists of genes controlled by several independent eQTLs, at least in blood, for example, PMID 31123039 (although I can imagine that LD noise might difficult effect size estimation of each variant)

-	In this regard, the permuted null distributions (with LD-matched regions) in Fig2B and C are clearly not centered around 0. Do the authors know the reason for this? Even if assigning GWAS effects randomy, longer LD blocks have a higher chance to land a larger effect?

-	Fig 2D is very interesting. I wonder if there is an excess of not consistent beta-genes (beyond expected by random sampling)? Do these correspond to weaker (likely buffered) GWAS signals? Are these genes less findable through colocalization and TWAS? I am not requesting for a full-fledged analysis here, but deeper understanding of the situation for these genes might be informative

-	Supp. Fig S9 C and D imply that many associations with strong evidence for colocalization (rcp >50%) fare poorly in TWAS analysis. Is this expected? Do the graphs refer to association evidence for the same tissue (in both enloc and TWAS)?

-	Besides reporting a few replicated hits, can the authors add more general figures about the replicability of PrediXcan associations in the BioVU Biobank? This can be useful to understand better the factors that affect candidate gene detectability

-	I don´t think I understand the reasoning about the lower precision for colocalization-TWAS because "OMIM genes are depleted for regulatory variation" (page 13). Does this imply that not all the OMIM genes included in the silver lists were "findable" (they had eQTLs to start with)?

-	From the logistic regression analysis to understand the power of these methods to find causal genes, the authors imply that proximity to a GWAS hit seems to contribute in instances in which e and sQTLs are missing (and hence enloc, TWAS, etc cannot help). Would the dosage impact model also hold in these loci? Under this model, and considering that these GWAS loci lack molecular QTLs, are the GWAS signals weaker overall? I am not sure it can be done, but I wonder if deeper characterization of those instances can be helpful to determine what is going on, and why colocalization and/or TWAS do not help whereas proximity to a hit putatively does

-	I was surprised by the authors not sharing the imputed standardized association statistics for the GWAS studies included in the analyses. They do provide the pipeline to impute and harmonize them, but this might be difficult for small research groups. Considering all the genetic studies were harmonized to the variants available in the catalog of GTEx results, Sharing the resulting full association statistics for the GWAS studies would certainly be useful.

-	In this regard, from the text it is unclear why the 27 traits that are excluded (due to low overlap with GTEx variants) would present significantly deflated imputed p-values? If there is an obvious explanation (that I am missing), it might be a nice idea to discuss this in the supplementary text

-	A couple of minor typos. Line 13 in section 1.13.1 (OMIM) from Supp is missing an ending "in step 4,we mapped OMIM traits to OMIM genes using the". Also, in page 10 there is a missing reference in the sentence "the LD blocks are defined by analyzing empirical patterns of LD observed in 1000 Genomes [?]"

Finally, I would like to apologize for the delay in sharing my review. No one is having an easy time, and I am sorry if my late response has been a source of anxiety or any problem.

**Authors Response**

**Point-by-point responses to the reviewers’ comments:**

The authors presented the results combining GWAS and GTEx resources to identify plausible mechanisms underlying GWAS signals, demonstrated dose-dependent effects of RNA expression and splicing, and suggested combing multiple approaches to prioritize causal genes. The manuscript is of broad interest to readers in the field, and the manuscript is clearly written. While the analyses are carefully executed, I have several concerns on the results.

*We thank the reviewer for the positive feedback and the constructive criticisms.*

Major comments:

1. The authors did a lot of excellent analyses, however, most results in the section of "causal gene prediction and prioritization" has been reported by GTEx consortium [Aguet et al., 2019]. And similar results in the section of "Performance for identifying ground truth genes" has also been reported by Barbeira et al. 2020 BioRxiv. More justification of the novelty of this manuscript is required.

*The analysis reported in this paper was performed with an improved set of prediction models (fine-mapping-based instead of elastic net) that were generated after the results for the main paper were produced. The comparison with our original elastic net-based models is published as a separate paper in Genetic Epidemiology.*

*ACTION: We have clarified this in the results section.*

*We added the following paragraph.*

*“In depth comparison of these fine-mapped models with Elastic*

*Net-based and CTIMP \myref{utmost} is described by*

*\myref{barbeira:2020a}. The analysis presented here uses these updated models instead of Elastic Net as reported with the main GTEx publication \myref{gtex:2020}.”*

*Also in the supplement, we merged the figures presenting the summary of GWAS loci with associated and or colocalized genes for the fine-mapped-mashr and elastic net predictions and added to the caption the following:*

*“The number of loci with potential target genes according to both S-PrediXcan and enloc went up from 1989 with elastic net models to 2125 with the improved fine-mapped mashr models. The number of S-PrediXcan backed loci increased only 7, so that the added loci were mostly due to an increased overlap with enloc.”*

2. The authors reported that 50% of GWAS signals lead to a target gene through eQTL or sQTL. This is an important claim. And it sounds a lot better than 11% of heritability being mediated through cis-eQTLs (PMID: 32424349), although we can't compare the two metrics directly. I would like to see some discussion between them. In addition, it is not clear how meaningful this percentage is, because this analyses don't explicitly demonstrate what would be expected by chance given the polygenicity of the traits and the high-dense coverage of eQTLs/sQTLs across the genome.

*There are multiple factors that contribute to the apparent discrepancy, which include:*

*-	Our estimate also includes loci with splicing association/colocalization. Without splicing, the percentage would have been 39%.*

*-	GTEx v7 has half the sample size ov v8, so if the increase in regulability is linear as suggested by Yao et al results, we could extrapolate to about 22%*

*-	Pleiotropy: a variant may act through expression of the gene and also through direct effects (i.e. through other mechanisms not related to expression or splicing traits). This was discussed by Yao et al.*

*-	Attenuation bias leading to underestimation: it does not seem that Yao et al account for attenuation bias in their approach.*

*Error in variables are known to cause biased effect sizes.*

*ACTION:*

*we added the following paragraph to the results section “A recent report estimates that the proportion of trait variance explained by assayed transcriptome is on average 11\%\mycite{yao:2020}. Even though this number is not directly comparable with the proportion of loci with support from PrediXcan and\textit{enloc} some discussion is warranted. Differences may arise with our analysis may arise from the fact that 1) GTEx v8 doubles the number of samples with both genotype and RNAseq in v7, 2) we include links based on splicing in addition to expression, 3) a variant may act through both regulation of expression levels and other undetected mechanisms (pleiotropy), 4) attenuation bias may reduce the estimates given the error in eQTL effect sizes.”*

3. The authors used "ground truth" genes to compare the ability of different approaches to identify the causal gene that mediates the association between GWAS loci and the traits. The authors compared enloc, PrediXcan, and SMR. However, PrediXcan cannot distinguish between pleiotropy and linkage. It would be useful and informative to compare such methods which can distinguish pleiotropy from linkage (e.g., compare enloc and SMR+HEIDI). In addition, it would be useful to show the ROC curve by selecting a random set of genes that are included in the GWAS LD blocks.

*Enloc is a better alternative to SMR since it is a more statistically principled approach to determine what the authors of SMR call pleiotropy, in the paper we call it LD-contamination.*

*The issues with SMR is discussed in https://www.biorxiv.org/content/10.1101/2020.07.01.182097v1 . Mainly, that “Although the idea is intuitive, the HEIDI procedure is set up to claim colocalization by accepting the null hypothesis.”*

*What we call pleiotropy (same variant affecting gene expression levels and the complex trait but without a causal link between the gene and the complex trait) cannot be resolved by statistical approaches alone.*

*Ideally, we would like to run a more fine grained analysis as suggested by the reviewer, but the scarcity of ground truth gene/trait pairs makes it infeasible at the moment.*

*ACTION:*

*We added the following paragraph to the colocalization section in results “For a more extensive discussion on the benefits of Bayesian colocalization methods and comparison of \textit{enloc} to other colocalization approaches including SMR-HEIDI see (Hukku et al).”*

*ACTION: we added the following ROC and PR curves with permuting the scores of genes within each of the GWAS locus as a supplementary figure. We did not add to the main figure to keep the figures cleaner.*

Minor comments

4. Justification of the rcp threshold of 0.5 is required.

*Like any threshold, 0.5 is somewhat arbitrary. Given the degree of colocalization of height from two different datasets (GIANT and UK Biobank) where half of the GIANT loci had colocalization probability below 0.001, we considered 0.5 a quite conservative threshold.*

*ACTION: clarified this in the text.*

5. For the low colocalization of height signals between GIANT and UKB, the authors aimed to demonstrate that enloc is conservative, although the authors also acknowledged that uncorrected population structure in GWAS meta-analyses may also partly contribute to this. However, the data could not fully support this hypothesis. In other words, we can also interpret the results like this "population structure in GIANT data may be the major confounder of low colocalization.".

*The main message we wanted to get across is that, in practice, given current limitations and heterogeneity of GWAS, colocalization and fine-mapping studies must be interpreted with care. For practitioners, imperfect GWAS is a reality that must be reckoned with.*

*ACTION: added the following clarification in the results section. “Notice that this limitation is not inherent to the colocalization method itself but the limitation of currently available large scale GWAS meta analysis results”*

6. It is not clear how to derive the ROC curve if one GWAS locus contains multiple highly corelated genes.

*We used a universal cutoff across all GWAS loci, hence highly correlated genes would be classified as causal or non causal as a cluster.*

*ACTION:*

*Add clarification in the methods/supplement section*

7. While the section of "tissue enrichment of GWAS signals" appears to be sound, it lacks of the detail of the method, making it difficult to follow, although they can be found in Supplementary Text. More details of method are required in the main text to improve the readability.

*ACTION: We moved the description of the casual tissue method from the supplement to the main results section.*

8. On page 3 line 35, "Here we develop novel methods, approach, and resources". This is a strong statement given that all the data have been made publicly available by GTEx consortium and that all the methods used in this manuscript are developed by previous studies.

*ACTION: we have modified the sentence as follows*

*“Here, we present methods and resources that help elucidate how genetic variants associated with gene expression (cis-eQTLs) or splicing (cis-sQTLs) contribute to, or mediate, the functional mechanisms underlying a wide array of complex diseases and quantitative traits.”*

9. A typo in Figure S5. The circle represents all variants and square represents GWAS catalogue variant.

*Fixed.*

Reviewer 2

Comments to author:

Barbeira, Bonazzola, Gamazon, Liand, Park and collaborators have carried out a thorough evaluation of the impact of the genetics of gene expression on complex traits and diseases. Using data from GTEx v8, they first uncover three pieces of evidence that imply a gene-dosage relationship between expression and phenotypes. Second, they perform a comprehensive query for causal genes at GWAS loci using colocalization and association with predicted gene expression, including replication in the BioVU cohort (for a number of traits available). Besides proposing candidate genes for many GWAS loci, these analyses provide many useful insights about the practices, features and methodologies that work best in regards to these approaches. Yet, a large fraction of loci associated with complex traits remains unresolved, and the low overlap in candidates observed for two large GWAS for height put an upper-bound for the potential of these methodologies to settle this aspect (here the authors conclude that further characterization of the genetic basis of gene expression is necessary). Evaluation in two independent sets of likely causal genes (elegantly ascertained 'silver genes') confirms these propositions. Third, and finally, they demonstrate that the catalogues of QTLs detected by GTEx provide insights about the tissue nature underlying complex traits and diseases.

*We thank the reviewer for the positive feedback.*

This is an excellent study. The authors accomplish what I think is the most comprehensive examination of the many ways in which knowing about the genetic architecture of gene expression across tissues is useful to learn about the genetic basis of complex traits and disease susceptibility. Almost as important, the authors also provide a wealth of results across GTEx tissues and GWAS traits for several of the most used methods in statistical genetics, including coloc, dap-g fine-mapping, prediXcan, etc. Benchmarking them, making available the models to run new TWAS, providing a large catalogue of candidate genes, etc, all make of this work an excellent resource for the community. This is obviously possible thanks to the work of many different analysts, but I feel compelled to congratulate all the authors for putting this out.

*Thank you. It is rewarding to hear that our effort is appreciated.*

The methods and analyses seem totally correct to me. The manuscript is well written and easy to follow (even if at times the main text is a bit lacking in methodological details, everything is covered with great detail in the supplementary materials). Rather than important flaws to be addressed, I have compiled a list of general comments and questions for the authors.

-	The observation that sets the scene in the main text (eQTL and sQTLs have a strong enrichment for good GWAS pvalues) refers to FigS5. The numbers in the figure are impressive. This is an important problem, since many researchers use blindly the evidence from QTL association to blindly infer causal genes. I would suggest discussing one or two examples of this enrichment in the main text (bottom in page5) so that readers can understand better the need for this manuscript

*ACTION: [ ] move figure to main results*

*Added the following paragraph: Fig.~\myref{SFIG-ENRICHMENT} also illustrates the dangers of using a naive approach to assigning causal genes to GWAS variants that are associated with expression or splicing, especially when using loose p-value thresholds. For example, with a p-value threshold of 0.05, over 97\% of common variants will be assigned some gene in some tissue associated at that level.”*

-	The concordance of effects in the allelic series analysis is a beautiful result. Why did the authors restrict to situations with two independent eQTLs? Is it due to lack of genes with >2 fine-mapped variants in GTEx (sample size)? Authors could use here known lists of genes controlled by several independent eQTLs, at least in blood, for example, PMID 31123039 (although I can imagine that LD noise might difficult effect size estimation of each variant)

*We started using the variants with exactly two independent signals unintentionally.*

*ACTION: We updated the figure using all genes with multiple independent eQTLs.*

-	In this regard, the permuted null distributions (with LD-matched regions) in Fig2B and C are clearly not centered around 0. Do the authors know the reason for this? Even if assigning GWAS effects randomy, longer LD blocks have a higher chance to land a larger effect?

*Fig.2B: The positive correlation of the null distribution suggests that local LD is driving a portion of the positive correlation observed between eQTL and trait effect sizes.*

*Fig.2C: shows the estimated variance of the gene level effect, so they are restricted to be > 0. The strict deviation from 0 also indicates there is an effect of LD inflating on the observed estimates.*

*ACTION: added the following sentence to the Results*

*“The non-negative permuted correlation values indicate that local LD contributed to inflate the estimated mediation effect.”*

-	Fig 2D is very interesting. I wonder if there is an excess of not consistent beta-genes (beyond expected by random sampling)? Do these correspond to weaker (likely buffered) GWAS signals? Are these genes less findable through colocalization and TWAS? I am not requesting for a full-fledged analysis here, but deeper understanding of the situation for these genes might be informative

*Yes, the excess of points in the concordant quadrants suggests that. The genes that have non-concordant effects would be less findable by TWAS.*

*ACTION: We added the following to the Results section:*

*“Note that genes with discordant effects within the allelic series would be harder to detect and suggest more complex causal relationship or context specificity.”*

-	Supp. Fig S9 C and D imply that many associations with strong evidence for colocalization (rcp >50%) fare poorly in TWAS analysis. Is this expected? Do the graphs refer to association evidence for the same tissue (in both enloc and TWAS)?

*Supp Figure S9C and D show that among TWAS significant genes, a portion is also colocalized. A simpler way to see that most colocalized genes are also TWAS significant can be seen in the summary figure S10 where only 178 loci are colocalized and not significant compared to 1774 loci that are significantly associated but not colocalized.*

-	Besides reporting a few replicated hits, can the authors add more general figures about the replicability of PrediXcan associations in the BioVU Biobank? This can be useful to understand better the factors that affect candidate gene detectability

*We would love to run this analysis but, unfortunately, this is a bit outside of the scope of the current effort. Evidence of replication in BioVU was obtained for our phenomexcan paper (10.1126/sciadv.aba2083) where we use the BioVU-derived PheWAS catalog as the silver standard and show the predictive power of PhenomeXcan via AUC and precision-recall curves.*

*ACTION: added the following sentence to the paper*

*“Further confirming this statistical replication in BioVU, we used the PheWAS catalog as the silver standard and found an AUC curve of 0.62.”*

-	I don´t think I understand the reasoning about the lower precision for colocalization-TWAS because "OMIM genes are depleted for regulatory variation" (page 13). Does this imply that not all the OMIM genes included in the silver lists were "findable" (they had eQTLs to start with)?

*OMIM genes are not well predicted by regulatory variation which means that our method’s power is reduced.*

*ACTION: we simplified the paragraph which now says:*

*“Our current understanding of gene function is biased towards protein-coding variants with very large effects, as reflected in the list of OMIM genes. Genes associated to rare severe disease tend to be depleted of regulatory variation \mycite{gnomad:2019, Mohammadi:2019kw}, which will decrease the performance of a QTL-based method \mycite{Mohammadi:2019kw}”*

-	From the logistic regression analysis to understand the power of these methods to find causal genes, the authors imply that proximity to a GWAS hit seems to contribute in instances in which e and sQTLs are missing (and hence enloc, TWAS, etc cannot help). Would the dosage impact model also hold in these loci? Under this model, and considering that these GWAS loci lack molecular QTLs, are the GWAS signals weaker overall? I am not sure it can be done, but I wonder if deeper characterization of those instances can be helpful to determine what is going on, and why colocalization and/or TWAS do not help whereas proximity to a hit putatively does

*Proximity is an empirical criterion that provides information on the causality of genes. This criterion helps both when*

*1.	We don’t have TWAS/colocalization results.*

*2.	TWAS/colocalization leads to multiple genes.*

*We know that GWAS signals tend to be more significant when the variant is also a QTL. It seems natural that the GWAS effect sizes will also be smaller. The relationship between lack of QTL signal and effect sizes of GWAS will be explored in future studies.*

*ACTION: added the following to the results section*

*“Proximity criterion also helps resolve cases when QTL data indicates multiple genes with similar significance.”*

-	I was surprised by the authors not sharing the imputed standardized association statistics for the GWAS studies included in the analyses. They do provide the pipeline to impute and harmonize them, but this might be difficult for small research groups. Considering all the genetic studies were harmonized to the variants available in the catalog of GTEx results, Sharing the resulting full association statistics for the GWAS studies would certainly be useful.

*They are available now on zenodo. Initially, we had shared them via box link due to size limitations in zenodo, but more recently, we have been able to upload the full dataset to zenodo as well. https://zenodo.org/record/3629742#.XjCh9OF7m90*

*ACTION: we have updated the zenodo link in the paper’s analysis repository https://github.com/hakyimlab/gtex-gwas-analysis*

-	In this regard, from the text it is unclear why the 27 traits that are excluded (due to low overlap with GTEx variants) would present significantly deflated imputed p-values? If there is an obvious explanation (that I am missing), it might be a nice idea to discuss this in the supplementary text

*Imputation of Zscore worked reasonably well for most traits. But 27 of them, the older GWAS studies, showed significant deflation and we decided to exclude them from the analysis presented in this paper. However, the overall results remain substantively unchanged when we add them.*

*ACTION: We added the following clarification in the supplement: “The main reason to drop the 27 traits were to keep the consortium's multiple papers consistent. None of the conclusions in this paper changed when including all 114 traits.”*

-	A couple of minor typos. Line 13 in section 1.13.1 (OMIM) from Supp is missing an ending "in step 4,we mapped OMIM traits to OMIM genes using the". Also, in page 10 there is a missing reference in the sentence "the LD blocks are defined by analyzing empirical patterns of LD observed in 1000 Genomes [?]"

*ACTION: fixed*

Finally, I would like to apologize for the delay in sharing my review. No one is having an easy time, and I am sorry if my late response has been a source of anxiety or any problem.

**Second round of review**

**Reviewer 1**

The authors have satisfactorily responded to all my comments and made the necessary changes to the manuscript. The revised version of the manuscript appears to be good.
